# Comparative effectiveness and safety of biologics and targeted small-molecule therapies plus stable background therapy in systemic lupus erythematosus: a systematic review and network meta-analysis

**DOI:** 10.3389/fimmu.2026.1739946

**Published:** 2026-05-01

**Authors:** Wenjuan Li, Yanhong Zhao, Siyan Shu, Ran Li, Yu Huang, Yuanyuan Gong, Guanhua Zhao, Yuxin Fan, Jianchang He

**Affiliations:** 1 School of Chinese Materia Medica, Yunnan University of Chinese Medicine, Kunming, China; 2 Research Center of Clinical Pharmacology, The First Affiliated Hospital of Yunnan University of Chinese Medicine, Kunming, China; 3 School of Pharmaceutical Sciences and Yunnan Key Laboratory of Pharmacology for Natural Products, Kunming Medical University, Kunming, China; 4 Research Center for Early Clinical Trials of Drugs (Vaccines), The Affiliated Anning First People’s Hospital, Kunming University of Science and Technology, Kunming, China; 5 Monash University, Faculty of Pharmacy and Pharmaceutical Sciences, Melbourne, Australia; 6 Department of Clinical Pharmacy, 920th Hospital of Joint Logistics Support Force, Kunming, China

**Keywords:** biologics, efficacy, safety, SLE, targeted small molecule drugs

## Abstract

**Objective:**

To compare the efficacy and safety of biologics and targeted small molecule drugs plus stable background therapy for the systemic lupus erythematosus (SLE).

**Methods:**

A systematic search was conducted across PubMed, EMBASE and Cochrane Library for eligible randomized controlled trials (RCTs), and a network meta-analysis (NMA) was performed to investigate the efficacy and safety of biological agents and targeted small molecule drugs added to stable background therapy in SLE. The evaluation indicators included the rates of SLE Responder Index (SRI-4) response, BILAG-based Composite Lupus Assessment (BICLA) response, Cutaneous Lupus Erythematosus Disease Area and Severity Index-50 (CLASI-50), Lupus Low Disease Activity State (LLDAS), adverse events (AEs), serious adverse events (SAEs) and infection-related adverse events.

**Results:**

A total of 32 studies were included, involving 17,121 patients. For SRI-4 response, Telitacicept was superior to Belimumab and Ustekinumab outperformed Epratuzumab. Upadacitinib demonstrated superior efficacy versus Baricitinib for both BICLA response and LLDAS attainment. Deucravacitinib and Anifrolumab were more effective for CLASI-50 achievement than Baricitinib. Anifrolumab, Iberdomide, and Telitacicept were associated with a higher incidence of AEs (e.g., upper respiratory tract infections, urinary tract infection, and herpes zoster) compared with other interventions, which may be related to their immunomodulatory mechanisms of action. Cenerimod was associated with the lowest risk of SAEs, and IL-2 showed the lowest risk of infection-related AEs.

**Conclusions:**

Telitacicept and Ustekinumab demonstrated superior efficacy for SRI-4 response; Upadacitinib superior for BICLA response and LLDAS achievement; Deucravacitinib and Anifrolumab showed advantages in CLASI-50 improvement, suggesting therapeutic potential for SLE with cutaneous manifestations. Although current findings indicate that these interventions have favorable efficacy and safety profiles, their long-term efficacy and safety still require further investigation and validation in the future.

**Systematic Review Registration:**

https://www.crd.york.ac.uk/PROSPERO/, identifier CRD42024594766.

## 1 Introduction

Systemic lupus erythematosus (SLE) is a multifactorial autoimmune disease characterized by autoantibody production and multisystem involvement, such as, mucocutaneous involvement, kidney failure, pulmonary hypertension, and cardiac failure (1). SLE affects an estimated 3.4 million people worldwide, with approximately 400,000 new cases diagnosed each year (2). It most commonly occurs among women during the childbearing age between 15–44 years (1). However, the pathogenesis of SLE remains unclear and is usually believed to be associated with genetic factors, epigenetic factors, environmental triggers, and hormonal factors (3). Despite significant advances in the diagnosis and management of SLE, the disease remains a major health burden.

Although glucocorticoids, antimalarials, and immunosuppressants remain the foundation of SLE therapy (4), their long-term use is constrained by adverse effects (e.g., infections and myelosuppression) (5) and incomplete disease control. In recent years, as the comprehension of SLE pathogenesis has advanced, cytokine-related dysregulations have been recognized as pivotal pathogenic factors (6), and treatment strategies have shifted from chronic steroids and high-dose chemotherapeutic regimens to targeted biologic therapies (7). Currently, a growing number of researches report the efficacy and safety of biologic agents and targeted small-molecule drugs added to stable background therapy in SLE. These agents target specific immune pathways implicated in SLE pathogenesis and have demonstrated promising efficacy and safety profiles in individual trials. However, direct head-to-head comparisons between biologics and targeted small molecules are lacking, and conventional meta-analyzes restricted to pairwise comparisons cannot integrate the full evidence base. Therefore, a comprehensive Network Meta-analysis (NMA) is necessary to simultaneously compare these interventions and generate a hierarchical ranking of their relative efficacy and safety. Notably, two previous NMAs (8, 9) separately comparing the efficacy and safety of different biological agents and evaluating the safety and effectiveness of various targeted small molecule drugs for SLE. Furthermore, although a recent study (10) also has compared the safety and efficacy of biologics, target therapy, and conventional therapies for SLE, the number of included studies, intervention measures, and related NMA analyzes were relatively limited.

Therefore, this NMAs was conducted to systematically and comprehensively evaluate the efficacy and safety profiles of biologic agents and targeted small-molecule drugs in SLE added to stable background therapy based on available RCTs, looking forward to provide further evidence and reference for clinical strategies and future researches.

## 2 Methods

### 2.1 Search strategy

A systematical search of the following electronic databases (EMBASE, PubMed, and the Cochrane Library) was conducted for literature published from inception to 31 December, 2025 in this study. The search terms utilized in this study included “Systemic Lupus Erythematosus”, “Biologics”, “Rituximab”, “Belimumab”, “Obinutuzumab”, “Anifrolumab”, “Voclosporin”, “Baricitinib”, “Deucravacitinib”, “Janus Kinase Inhibitors”, and “BTK Inhibitor”. Detailed search strategy was provided in **Supplementary Appendix 1**. The study protocol was pre-registered at the International Prospective Register of Systematic Reviews (PROSPERO) (registration number: CRD42024594766).

### 2.2 Inclusion and exclusion criteria

The inclusion criteria were as follows: (1) Adults diagnosed with SLE (≥ 18 years of age); (2) The patients included in the study administrated with biological agents or targeted small molecule drugs or a placebo or other interventions under stable background therapy (such as glucocorticoids, antimalarial agents, immunosuppressive medications, and non-steroidal anti-inflammatory drugs, administered individually or in combination); (3) At least one of the following outcomes was reported: (a) Systemic Lupus Erythematosus Responder Index (SRI-4) response rate based on the SELENA-SLEDAI score or the SLEDAI-2K score, (b) The British Isles Lupus Assessment Group (BILAG) based Composite Lupus Assessment response (BICLA), (c) CLASI-50, defined as a more than 50% improvement in the Cutaneous Lupus Erythematosus Disease Area and Severity Index (CLASI) activity, (d) Lupus Low Disease Activity State (LLDAS) response; (e) adverse events (AEs). Details were provided in **Supplementary Appendix 2**.

The exclusion criteria were as follows: (1) active severe lupus nephritis, active severe central nervous system lupus; (2) animal studies, case reports, or systematic reviews; (3) duplicate publications or incomplete data; (4) studies not reporting relevant outcomes.

### 2.3 Data extraction and quality assessment

Two researchers independently extracted related data, including (1) basic study characteristics (authors, publication year, study design, diagnostic criteria, interventions, treatment duration, and outcome indicators); (2) demographic information of included studies (sample size, age and gender distribution). For trials in which outcomes were presented only in graphical format, GetData Graph Digitizer (v2.24) was utilized to extract data.

The qualities of all included studies were assessed using the Cochrane Library’s recommended risk of bias evaluation tool (11). This tool mainly includes randomized sequence generation, allocation concealment, blinding, completeness of outcome data, selective reporting of results and the presence of other biases. Two investigators comprehensively evaluated the included literature according to established criteria, assigning a risk level of “low risk,” “high risk,” or “unknown risk” to each item. Furthermore, the Grading of Recommendations Assessment, Development and Evaluation (GRADE) tool was also used to assess the quality and strength of the evidences of main outcomes. Evidence was classified as high quality, moderate quality, low quality, very low quality. Any disagreements that arose were addressed through collaborative discussions between these two investigators or with the participation of a third investigator.

### 2.4 Statistical analysis

The treatment effects of several interventions were compared directly and indirectly by NMA. The dichotomous variables were expressed as relative risk (RR) and corresponding 95% confidence intervals (CIs). If the 95% CI of the RR did not contain 1, the differences were considered statistically significant. The Haldane-Anscombe continuity correction (adding 0.5 to all cells) was applied to handle zero cells. In the NMA, the network diagrams help visualize relationships between interventions, where the sizes of the points represent the number of studies or sample sizes, while the thicknesses of the lines indicate the quantity of the included literature regarding the two corresponding interventions. The thicker the line, the larger the number of studies on these two interventions. Meanwhile, the size of a point represents the sample size of the corresponding intervention, in other words, the larger the point, the greater the sample size of that intervention. When a closed loop exists, the Z-test is employed for the consistency evaluation. If *P* > 0.05, it suggests that the results demonstrate significant consistency. Conversely, it implies that there is inconsistency (12). To achieve a more precise quantification of this inconsistency, a node-splitting analysis was performed. Considering assumed clinical variability between the studies, all primary NMA analyzes were performed using a random-effects model. In this study, the surface under the cumulative ranking curve (SUCRA) was used to assess and rank the effectiveness of various interventions. Additionally, A hierarchical ranking diagram of efficacy and safety was then produced to visually depict the results. A higher SUCRA value indicates a better efficacy. Importantly, SUCRA values represent relative ranking probabilities rather than formal statistical tests of superiority; interpretation of small differences between interventions must account for overlapping confidence intervals and the overall uncertainty of effect estimates.

The STATA 15.0 (StataCorp LLC, College Station, TX, USA) software was used for statistical analysis of NMA, global inconsistency, local inconsistency, and comparison-adjusted funnel plot. Using R 4.4.1(R Foundation for Statistical Computing, Vienna, Austria) to conduct Egger’s linear regression test for publication bias, and to assess between-study heterogeneity using the I² statistic and Cochran’s Q test. For outcomes where Egger’s test indicated potential small-study effects (*P* < 0.10) or where the limited number of studies precluded reliable *P*-value estimation, leave-one-out sensitivity analyzes were performed by sequentially omitting each included study to verify the stability of intervention rankings.

## 3 Results

### 3.1 Description of included studies

**Figure 1:**
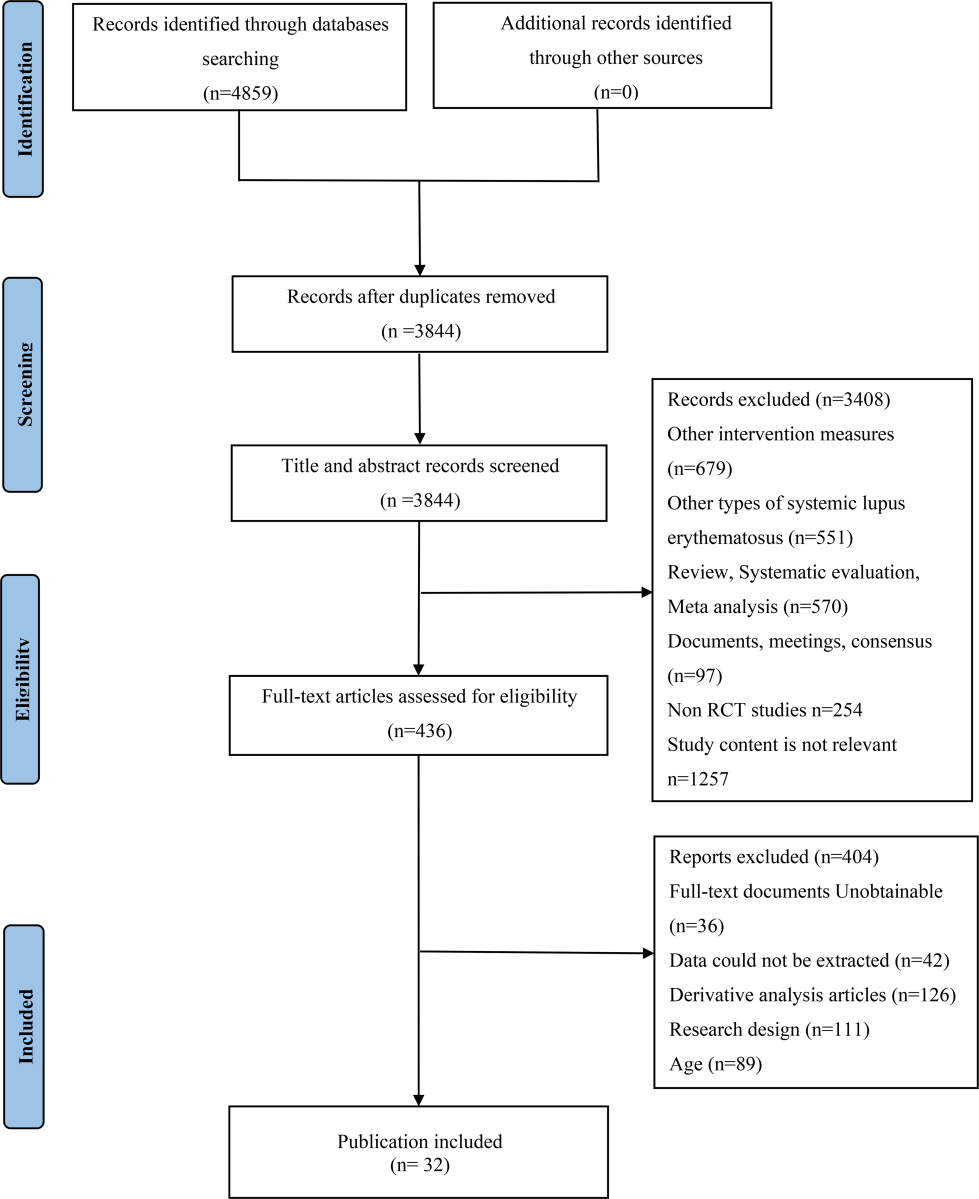
Flow chart of study search and selection.

**Figure 2:**
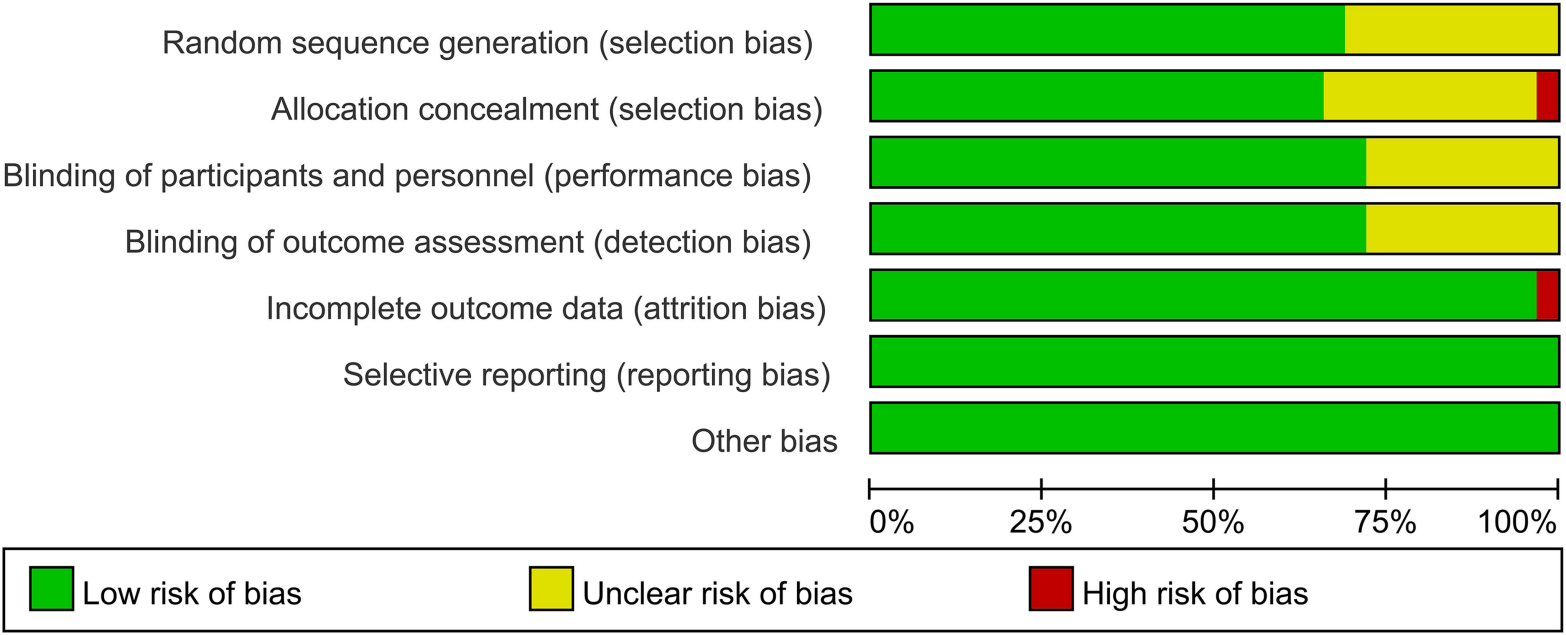
Risk of bias graph and summary of the included studies.

Of 4,859 records identified 1,015 duplicates were removed and 436 full-text articles were screened. Thirty-two publications (13–44) met the eligibility criteria, including 17,121 participants (predominantly women). The PRISMA flow diagram is presented in **Figure 1**. Baseline characteristics are provided in **Table 1**; data on background medications and ethnicity distributions are shown in **Supplementary Appendix 3**. Most trials adequately reported allocation concealment; the overall risk-of-bias assessments are summarized in **Figure 2**. A bias risk rating for each study was provided in **Supplementary Appendix 9**. According to the GRADE assessment, the overall quality of evidence was downgraded primarily due to the following three reasons: first, insufficient information in some studies to judge the risk of bias regarding allocation concealment or incomplete outcome data; second, the 95% confidence intervals of the effect measures crossed the line of no effect; and third, indirectness arising from the study network structure—all comparisons were mediated through placebo, lacking direct head-to-head evidence between different active treatments (further details were available in **Supplementary Appendix 10**).

**Table 1:** Baseline characteristics of patients included in the selected studies.

Study	Trial registration	Study design	Interventions	N	Age(years)	Female (n, %)	Disease duration (years), mean (SD)	SLEDAI-2K score (mean ± SD)	≥1 BILAG A or B score [N (%)]	SELENA-SLEDAI score	PGA score (mean ± SD)	Outcomes
Eric Morand, 2023 (13)	NCT03252587	multicenter RCT	deucravacitinib 3mg; deucravacitinib 6mg; deucravacitinib 12mg; placebo	363	40.2 ± 11.9; 40.9 ± 12.5; 39.0 ± 10.6; 40.1 ± 13.1	85(93.4); 88(94.6); 81(91.0); 80(88.9)	<3 years 24 ± 26.4; 27 ± 29.0; 32 ± 36.0; 26 ± 28.9; 3 to 6 years 13 ± 14.3; 19 ± 20.4; 13 ± 14.6; 16 ± 17.8; >6 years 54± 59.3; 47 ± 50.5; 44 ± 49.4; 48 ± 53.3	11.1 ± 3.2; 10.8 ± 3.2; 10.7 ± 3.0; 10.8 ± 3.1	BILAG-2004 A/B grades ≥1 A grade 51 (56.0)44 (47.3)51 (57.3)51 (56.7) No A grade or ≥2 B grades 40 (44.0)46 (49.5)37 (41.6)39 (43.3)	NR	1.80 ± 0.3; 1.84 ± 0.4; 1.86 ± 0.4; 1.82 ± 0.4	②③④⑤⑥
Eric F Morand, 2023 (15)	NCT03616912	multicenter RCT	Placebo; Baricitinib 4mg; Baricitinib 2mg	760	42.0 ± 12.0; 42.9 ± 12.4; 41.5 ± 12.9	237 (94); 238 (93); 237 (94)	9.4 ± 7.5; 9.2 ± 7.7; 8.8 ± 8.2	10.1 ± 3; 10.3 ± 3; 10.0 ± 3	≥1 BILAG A scores 154 (61)173 (68)154 (61) ≥2 BILAG B scores 81 (32)69 (27)85 (34)	NR	1.8 ± 0.5; 1.8 ± 0.4; 1.8 ± 0.5	②③④⑤⑥
Michelle Petri, 2023 (16)	NCT03616964	multicenter RCT	Placebo; Baricitinib 4mg; Baricitinib 2mg	775	43.5 ± 13.5; 42.8 ± 13.0; 42.2 ± 12.1	241 (94); 246 (94);245 (95)	9.0 ± 8.3; 8.7 ± 7.7; 8.5 ± 7.7	10.1 ± 3.2; 10.1 ± 3.4; 10.1 ± 3.0	≥1 BILAG A scores 172 (67)188 (72)172 (67) ≥2 BILAG B scores 73 (29)64 (25)76 (29)	NR	60.0 ± 14.6; 61.0 ± 12.5; 58.8 ± 14.6	②③④⑤⑥
Daniel J Wallace, 2018 (17)	NCT02708095	multicenter RCT	Placebo; Baricitinibb 4mg; Baricitinib 2mg	314	44.9 ± 12.8; 43.2 ± 11.0; 45.0 ± 12.4	99 (94.3); 96 (91.4); 99 (95.2)	9.7 ± 7.7; 11.8 ± 9.1; 11.5 ± 10.3	8.9 ± 2.9; 8.8 ± 3.4; 9.0 ± 3.3	≥1 A or ≥2 B BILAG scores 62 (59)56 (53)69 (66)	NR	49.5 ± 16.9; 48.8 ± 15.8; 51.7 ± 16.0	②⑤⑥
Joan T Merrill, 2024 (14)	NCT03978520	multicenter RCT	high dose ABBV-599; Upadacitinib 30mg; Placebo	205	42.7 ± 11.3; 42.5 ± 11.9; 41.7 ± 12.1	62 (91.2); 57 (91.9); 75 (100)	9.6 ± 7.5; 11.0 ± 6.9; 9.4 ± 7.3	8.9 ± 2.8; 9.0 ± 2.8; 9.1 ± 3.9	NR	NR	NR	②③④⑤
Richard A Furie, 2021 (18)	NCT02804763	multicenter RCT	Placebo;dapirolizumab pegol 6mg/kg;dapirolizumab pegol 24mg/kg;dapirolizumab pegol 45mg/kg	176	42.7 ± 12.5; 40.5 ± 11.7; 42.6 ± 10.5; 39.0 ± 13.1	39 (90.7); 40 (93.0); 39 (88.6); 42 (91.3)	median year (min–max) 5.4 (0.1–30.0); 5.0 (0.2–27.8); 5.1 (0.3–27.0); 8.2 (0.3–25.0)	10.7 ± 3.4; 11.4 ± 2.4; 9.9 ± 2.5; 11.1 ± 3.4	NR	NR	NR	②③
Daniel J Wallace, 2009 (19)	NCT00071487	multicenter RCT	Placebo; Belimumab 1.0mg/kg; Belimumab 4.0mg/kg; Belimumab 10.0mg/kg	449	42.2 ± 10.9; 42.0 ± 11.7; 42.6 ± 10.7; 41.8 ± 11.7	90.3; 93.9; 94.6; 94.6	8.1 ± 7.4; 8.5 ± 7.2; 10.1 ± 9.2; 8.5 ± 8.0;	NR	≥1 BILAG A or B score 90.3;95.6;96.4;97.5	9.5 ± 0.5; 9.9 ± 0.4; 9.4 ± 0.5; 9.5 ± 0.4	1.4 ± 0.05; 1.6 ± 0.05; 1.5 ± 0.05; 1.5 ± 0.05	⑥
Richard A Furie, 2019 (20)	NCT02446912	multicenter RCT	Placebo; Anifrolumab 300mg	364	41.0 ± 12.3; 42.0 ± 12.0	171 (93); 165 (92)	Median month (range) 79.5 (4–503); 88.0 (0–450)	11.5 ± 3.5; 11.3 ± 4.0	BILAG-2004 no A and at least two B items 84 (46);79 (44)	NR	NR	②③④⑥
Eric F Morand, 2020 (21)	NCT02446899	multicenter RCT	Placebo; Anifrolumab 300mg	362	41.1 ± 11.5; 43.1 ± 12.0	170 (93.4); 168 (93.3)	Median month (range) 78.0 (6–494); 94.5 (6–555)	11.5 ± 3.9; 11.4 ± 3.6	BILAG-2004 ≥1 A item 95 (52.2); 81 (45.0) No A items and ≥2 B items 78 (42.9); 91 (50.6)	NR	1.76 ± 0.40; 1.68 ± 0.41	②③④⑥
Sandra V Navarra, 2011 (22)	NCT00424476	multicenter RCT	Placebo; Belimumab 1mg/kg; Belimumab 10mg/kg	865	36.2 ± 11.8; 35.0 ± 10.6; 35.4 ± 10.8	270 (94); 271; (94); 280 (97)	5.9 ± 6.2; 5.0 ± 4.6; 5.0 ± 5.1	NR	BILAG 1A or 2B score 166 (58); 166 (58); 172 (59)	9.7 ± 3.6; 9.6 ± 3.8; 10.0 ± 3.9	1.4 ± 0.5; 1.4 ± 0.5; 1.4 ± 0.5	①⑥
Richard Furie 2011 (23)	NCT00410384	multicenter RCT	Placebo; Belimumab 1mg/kg; Belimumab 10mg/kg	819	40.0 ± 11.9; 40.0 ± 11.4; 40.5 ± 11.1	252 (91.6); 253 (93.4); 259 (94.9)	7.4 ± 6.7; 7.9 ± 7.1; 7.2 ± 7.5	NR	BILAG 1A or 2B score 187 (68.0); 173 (63.8); 160 (58.6)	9.8 ± 4.0; 9.7 ± 3.7; 9.5 ± 3.6	1.5 ± 0.5; 1.4 ± 0.5; 1.4 ± 0.5	①⑥
William Stohl, 2017 (24)	NCT01484496	multicenter RCT	Placebo; Belimumab 200mg	836	39.6 ± 12.61; 38.1 ± 12.10	268 (95.7); 521 (93.7)	median year (range) 4.6 (0–38); 4.3 (0–35)	NR	NR	10.3 ± 3.04; 10.5 ± 3.19	1.5 ± 0.45; 1.6 ± 0.43	①⑥
Fengchun Zhang, 2018 (25)	NCT01345253	multicenter RCT	Placebo; Belimumab10mg/kg	677	31.7 ± 9.18; 32.3 ± 9.65	210 (92.9); 419 (92.9)	5.97 ± 5.19; 6.07 ± 5.04	NR	BILAG 1A or 2B score 108 (47.8); 204 (45.2)	10.2 ± 4.11; 9.8 ± 3.83	NR	①⑥
Joan T Merrill, 2018a (26)	NCT01972568	multicenter RCT	Placebo; Atacicept 75mg; Atacicept 150mg	306	40 ± 13.0; 37 ± 11.2; 39 ± 11.6	90 (90); 93 (91.2); 97 (93.3)	6.79 ± 7.65; 6.77 ± 6.85; 6.93 ± 6.95	10 ± 2.8; 10 ± 3.3; 10 ± 3.0	BILAG 2004 1A or 2B score60 (60.0); 57 (55.9); 72 (69.2)	NR	1.50 ± 0.452; 1.42 ± 0.532; 1.46 ± 0.460	②
Ronald F van Vollenhoven, 2018 (27)	NCT02349061	multicenter RCT	Ustekinumab; Placebo	102	40.0 ± 12.0; 42.9 ± 11.3	58 (97); 35 (83)	9.7 ± 8.3; 9.5 ± 7.2	10.6 ± 3.3; 11.4 ± 4.5	≥1 BILAG domain A 27 (45); 22 (52) ≥2 BILAG domain B 28(47); 16 (38)	NR	4.9 ± 1.6; 4.9 ± 1.6	②③⑥
Richard Furie, 2017 (28)	NCT01438489	multicenter RCT	Placebo; Anifrolumab 300mg; Anifrolumab 1000mg	305	39.3 ± 12.9; 39.1 ± 11.9; 40.8 ± 11.6	93 (91.2); 93 (93.9); 99 (95.2)	months 90.6 ± 86.3; 95.9 ± 76.8; 100.1 ± 90.3	11.1 ± 4.4; 10.7 ± 3.7; 10.9 ± 4.1	NR	NR	1.77 ± 0.44; 1.86 ± 0.39; 1.86 ± 0.39	②③④⑥
Ellen Ginzler, 2022 (29)	NCT01632241	multicenter RCT	Belimumab 10mg/kg; Placebo	448	38.6 ± 11.1; 39.3 ± 12.2	290 (97.0); 144 (96.6)	7.3 ± 7.08; 6.9 ± 7.38	NR	BILAG organ domain involvement ≥1A or 2B 215 (71.9); 107 (71.8)	9.9 ± 3.52; 10.2 ± 2.90	NR	⑥
Munther Khamashta, 2016 (30)	NCT01283139	multicenter RCT	Placebo; Sifalimumab 200mg; Sifalimumab 600mg; Sifalimumab 1200mg	431	38.4 ± 12.3; 39.9 ± 11.4; 40.0 ± 11.3; 39.4 ± 12.1	101 (93.5); 103 (95.4); 97 (89.8); 97 (90.7)	months 90.4 ± 74.9; 103.9 ± 84.9; 98.6 ± 82.6; 100.6 ± 94.9	11.1 ± 4.1; 11.0 ± 4.0; 11.3 ± 4.6; 11.7 ± 4.7	NR	NR	1.83 ± 0.39; 1.81 ± 0.37; 1.73 ± 0.39; 1.77 ± 0.40	②④⑥
Ian N Bruce, 2021 (31)	NCT02962960	multicenter RCT	Placebo; Anifrolumab 150mg; Anifrolumab 300mg	36	47.8 ± 14.2; 46.3 ± 9.1; 41.5 ± 9.2	8 (89); 12 (86); 12 (92)	5.5 ± 6.1; 10.6 ± 5.8; 9.4 ± 5.6	9.3 ± 4.3; 10.6 ± 5.7; 8.6 ± 3.1	NR	NR	NR	④⑥
Di Wu, 2024 (32)	NCT02885610	multicenter RCT	Placebo; Telitacicept 240mg; Telitacicept 160mg; Telitacicept 80mg;	249	34.9 ± 9.6; 33.5 ± 9.8; 33.5 ± 10.3; 33.8 ± 8.9	58 (93.5); 59 (95.2); 61 (96.8); 57 (91.9)	8.79 ± 5.87; 6.64 ± 5.36; 6.67 ± 5.21; 6.47 ± 5.46	NR	at least two BILAG B and one BILAG A 35 (56.5); 38 (61.3); 40 (63.5); 37 (59.7)	NR	1.80 ± 0.40; 1.88 ± 0.48; 1.87 ± 0.43; 1.81 ± 0.46	①⑥
Viktoria Hermann, 2019 (33)	NCT02472795	multicenter RCT	Placebo; Cenerimod 0.5mg; Cenerimod 1mg; Cenerimod 2mg; Cenerimod 4mg	67	41.0 ± 9.5; 41.4 ± 13.2; 37.0 ± 6.4; 39.2 ± 11.8; 41.7 ± 8.1	16 (94.1); 11 (91.7); 12 (100); 12 (92.3); 10 (76.9)	6.25 ± 5.88; 5.59 ± 6.42; 7.31 ± 6.11; 6.29 ± 5.49; 3.01 ± 2.48	NR	NR	NR	NR	⑥
Daniel J. Wallace, 2023 (34)	NCT02975336	multicenter RCT	Placebo; Evobrutinib 25mg QD; Evobrutinib 75mg QD; Evobrutinib 50mg BID;	469	40.2 ± 12.5; 38.8 ± 12.5; 41.5 ± 12.5; 42.2 ± 11.8	110 (94.0); 112 (94.9); 111 (94.9); 112 (95.7)	median year 51.6; 61.3; 69.2; 54.6	NR	Moderate (at least two BILAG B and no BILAG A)56 (47.9); 48 (40.7); 49 (41.9); 50 (42.7) Severe (at least one BILAG A) 22 (18.8); 28 (23.7); 27 (23.1); 22 (18.8)	NR	NR	②
Joan T. Merrill, 2022 (35)	NCT03161483	multicenter RCT	Iberdomide 0.45mg; Iberdomide 0.30mg; Iberdomide 0.15mg; Placebo;	288	46.4 ± 11.2; 44.7 ± 13.7; 43.8 ± 13.0; 43.4 ± 13.3;	79 (98); 77 (94); 41 (98); 81 (98)	median year(range) 9.0 (0.5–31.7); 7.3 (0.5–35.8); 7.3 (0.9–35.7); 5.7 (0.5–35.8)	9.5 ± 2.8; 9.6 ± 2.7; 9.5 ± 2.8; 9.8 ± 3.6	NR	NR	1.7 ± 0.5; 1.7 ± 0.3; 1.7 ± 0.4; 1.7 ± 0.4	②④⑥
Tomomi Tsuru, 2016 (36)	NCT01449071	multicenter RCT	Placebo; epratuzumab 100mg Q2W; epratuzumab 400mg Q2W; epratuzumab 600mg QW; epratuzumab 1200mg Q2W	20	46.3 ± 13.6; 34.8 ± 10.8; 45.5 ± 5.1; 36.0 ± 7.8; 37.0 ± 9.4	4 (100); 4 (100); 3 (75.0); 4 (100); 3 (75.0)	12.1 ± 9.8; 14.8 ± 12.5; 7.7 ± 6.1; 9.7 ± 4.4; 14.8 ± 4.4	NR	NR	NR	NR	⑥
Megan E B Clowse, 2017a (37)	NCT01262365	multicenter RCT	Placebo; epratuzumab 1,200mg QOW; epratuzumab 600mg QW	741	41.2 ± 12.8; 42.2 ± 11.7; 42.2 ± 11.4;	237 (95.2); 228 (93.4); 226 (91.1)	median year (range) 5.8 (0–36); 7.3 (0–34); 6.1 (0–43)	10.7 ± 4.1; 9.9 ± 3.7; 10.2 ± 3.6	≥1 BILAG-2004 A grad 139 (55.8); 142 (58.2); 147 (59.3)	NR	55.5 ± 12.9; 55.7 ± 14.3; 56,5 ± 14,9	②③⑥
Megan E B Clowse, 2017b (37)	NCT01261793.	multicenter RCT	Placebo; epratuzumab 1,200mg QOW; epratuzumab 600mg QW	788	41.1 ± 11.8; 40.8 ± 11.5; 41.2 ± 12.7	245 (93.2); 247 (94.6); 245 (92.8)	median year (range) 5.7 (0–37); 5.0 (0–29); 4.8 (0–42)	10.1 ± 3.6; 10.1 ± 3.8; 10.2 ± 3.6	≥1 BILAG-2004 A grad 157 (59.7); 148 (56.7); 161 (61.0)	NR	56.2 ± 14.4; 57.2 ± 14.0; 57.3 ± 15.6	②③⑥
Saira Z Sheikh, 2021 (38)	NCT01705977	multicenter RCT	Belimumab 10mg/kg Q2W; Placebo	4003	40.4 ± 12.75; 40.8 ± 12.74	1848 (92·35); 1853 (92·56)	median year (IQR) 5.1 (1.6–10.6); 5.3 (1.8–11.2)	NR	NR	7.8 ± 4.72; 7.9 ± 4.51	NR	⑥
Jing He, 2020 (39)	NCT02465580	Single center RCT	IL-2; Placebo	60	31.58 ± 9.25; 29.83 ± 9.72	27(90); 29(97)	months 66.7 ± 57.4; 63.6 ± 59.9	NR	≥1 BLIAG A or 2B scores 21 (70); 21 (70)	NR	median (range) 2.3 (1.55–2.75); 2.2(1–2.3)	①⑥
Jens Y Humrich, 2022 (40)	NCT02955615	multicenter RCT	ILT-101; Placebo	100	41.7; 40.4	49 (98); 42 (84)	10.7 ± 8.2; 8.4 ± 7.1	NR	≥1 BILAG A 24 (48); 19 (38); ≥2 BILAG B 11(22); 16 (32)	10.8 ± 3.9; 10.3 ± 3.2	1.9 ± 0.4; 1.9 ± 0.5	①⑥
Joan T Merrill, 2018b (41)	NCT01395745	multicenter RCT	Blisibimod 200mg; Placebo	442	36.7 ± 10.98; 35.6 ± 10.78	92.7; 94.9	NR	NR	NR	13.4 ± 4.31; 13.5 ± 4.01	1.59 ± 0.475; 1.64 ± 0.475	①⑥
D A Isenberg, 2015 (42)	NCT01196091	multicenter RCT	Tabalumab 120 Q2W;Tabalumab 120 Q4W;Placebo	1138	40 ± 13; 40 ± 11; 39 ± 12	354 (92.9); 352 (93.1); 360 (95.0)	8 ± 7; 8 ± 8; 6 ± 7	10.6 ± 3.7; 10.7 ± 3.9; 10.8 ± 4.0	228 (59.8); 228 (60.3); 220 (58.2)	10.2 ± 3.5; 10.4 ± 3.6; 10.7 ± 3.9	46.3 ± 15.7; 46.1 ± 16.2; 47.1 ± 16.1	①⑥
Yoshiya Tanaka, 2024 (43)	NCT05278663	multicenter RCT	Placebo; E6742 100mg; E6742 200mg	26	38.9 ± 9.20; 33.6 ± 12.97; 40.4 ± 11.17	8(88.9); 8(100.0); 8(88.9)	7.7 ± 6.10; 4.5 ± 3.62; 8.2 ± 6.96	7.8 ± 2.91; 8.6 ± 4.69; 6.7 ± 2.83	NR	NR	1.3 ± 0.50; 1.0 ± 0.27; 1.1 ± 0.40	③⑥
Daniel J Wallace, 2017 (44)	NCT01405196	multicenter RCT	Placebo; PF-04236921 10mg; PF-04236921 50mg	137	42.3 ± 13.0; 39.9 ± 11.5; 38.3 ± 10.5	38 (84.4); 43 (95.6); 43 (93.6)	9.1 ± 6.9; 7.9 ± 8.1; 7.5 ± 6.0	9.5 ± 2.2; 9.6 ± 2.7; 9.0 ± 2.7	BILAG A in≥1 organ system20 (44.4); 19 (42.2); 16 (34.0) BILAG B in ≥2 organ systems 25 (55.6); 27 (60.0); 33 (70.2)	NR	1.6 ± 0.4; 1.7 ± 0.4; 1.6 ± 0.4	②③

N number of patients, NR not reported, IV intravenous, SC subcutaneous, SD standard deviation, BILAG British Isles Lupus Assessment Group index, SELENA-SLEDAI Safety of Estrogens in Lupus Erythematosus National Assessment version of the SLE Disease Activity Index, ①SRI-4^#^, Systemic Lupus Erythematosus Responder Index, Composite responder index based on improvement in disease activity (at least 4 point improvement in SELENA-SLEDAI) without worsening of the overall condition (no worsening in PGA) or the development of significant disease activity in new organ systems (no new BILAG A or >1 new BILAG B). ② SRI-4^##^, Systemic Lupus Erythematosus Responder Index, Composite responder index based on improvement in disease activity (at least 4 point improvement in SLEDAI-2K score) without worsening of the overall condition (no worsening in PGA) or the development of significant disease activity in new organ systems (no new BILAG A or >1 new BILAG B). ③ BICLA, BILAG-based Composite Lupus Assessment response, ④ CLASI-50, defined as a more than 50% improvement in the Cutaneous Lupus Erythematosus Disease Area and Severity Index (CLASI) activity, ⑤ LLDAS, Lupus Low Disease Activity State, ⑥ AE, adverse events.

### 3.2 Efficacy results

#### 3.2.1 SRI-4 response

The stratified analysis for SRI-4 response rate was performed based on the SELENA-SLEDAI score or the SLEDAI-2K score. A sensitivity analysis combining these indices is shown in **Supplementary Appendix 11**.

**Figure 3:**
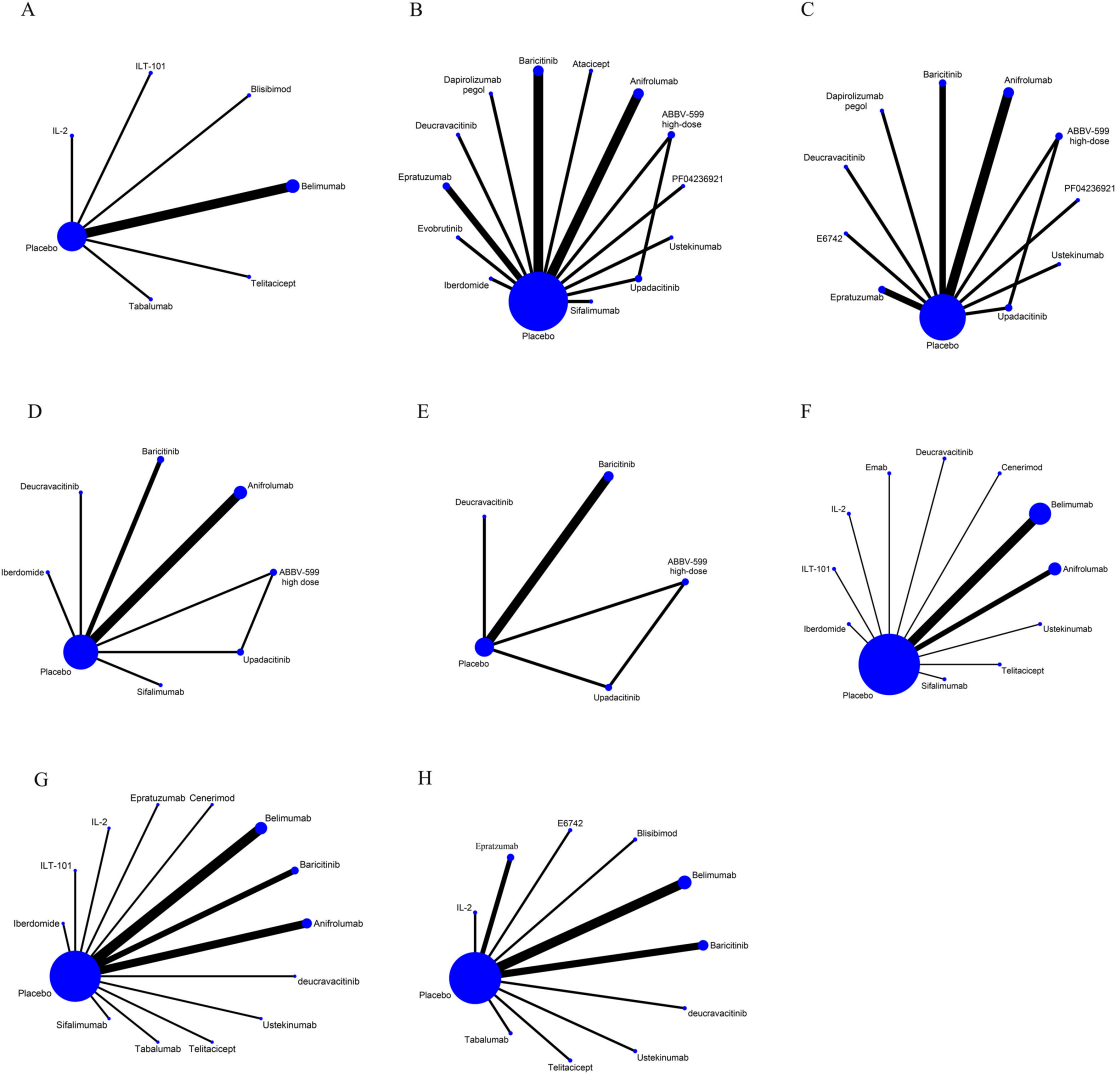
Network analysis for **(A)** SRI-4 response^#^, **(B)** SRI-4 response^##^, **(C)** BICLA response, **(D)** CLASI-50, **(E)** LLDAS, **(F)** AE, **(G)** SAEs, and **(H)** Infection related adverse reactions. The size of each node represents the number of participants, while the thickness of the line represents the number of studies directly comparing the two interventions. ^#^Composite responder index based on improvement in disease activity (at least 4 points improvement in SELENA-SLEDAI) without worsening of the overall condition (no worsening in PGA) or the development of significant disease activity in new organ systems (no new BILAG A or >1 new BILAG B). ^##^Composite responder index based on improvement in disease activity (at least 4 points improvement in SLEDAI-2K score) without worsening of the overall condition (no worsening in PGA) or the development of significant disease activity in new organ systems (no new BILAG A or >1 new BILAG B).

**Figure 4:**
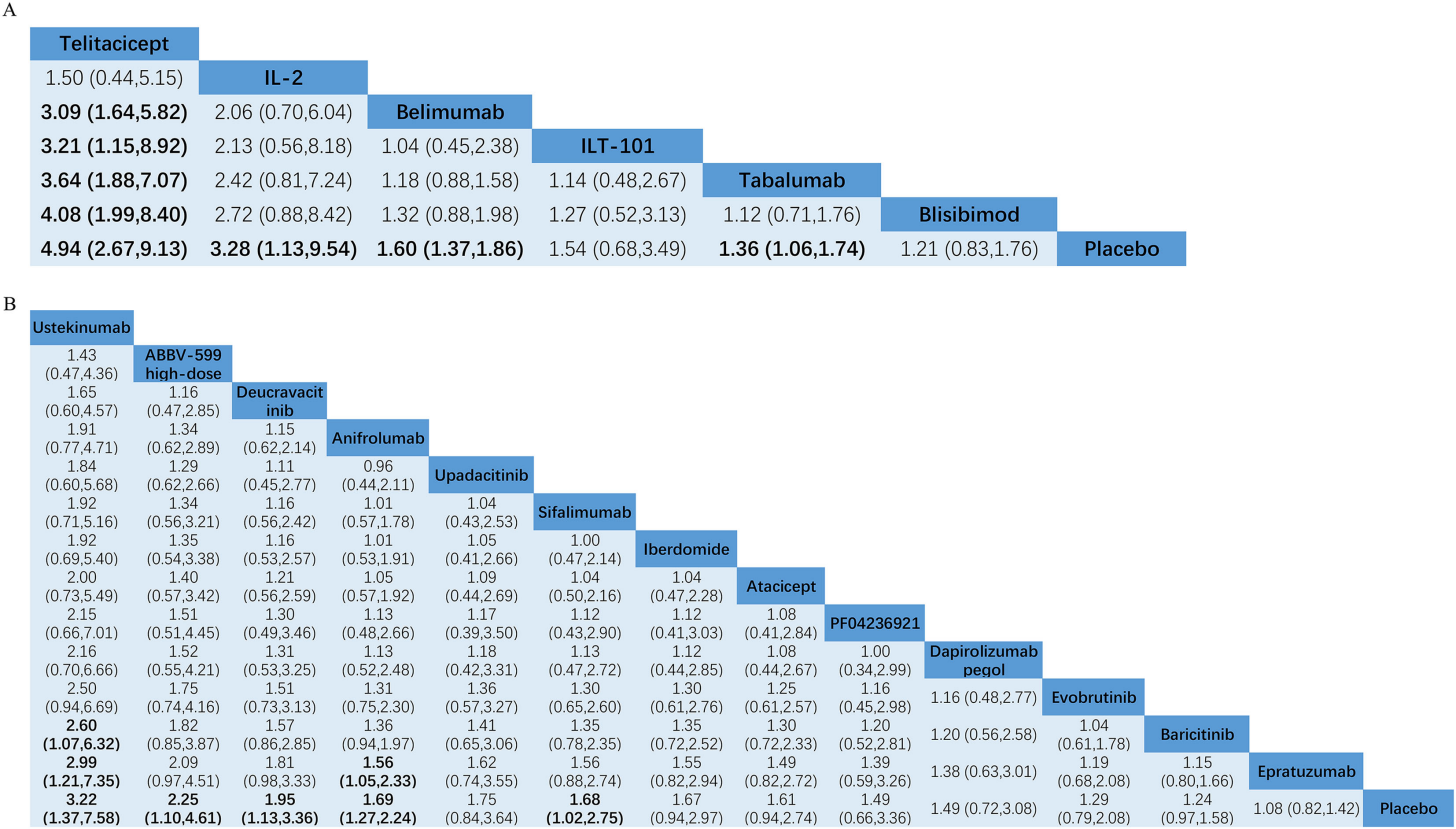
Results of network meta-analysis for **(A)** SRI-4 response^#^, **(B)** SRI-4 response^##^. estimates are presented as relative risk (RR) and 95% confidence intervals (CI; in parentheses). Comparisons should be read from left to right and the estimate is in the cell in common between the column-defining treatment and the row-defining treatment. If the 95% CI of the RR did not contain 1, the differences were considered statistically significant. Significant results are presented in bold. ^#^Composite responder index based on improvement in disease activity (at least 4 points improvement in SELENA-SLEDAI) without worsening of the overall condition (no worsening in PGA) or the development of significant disease activity in new organ systems (no new BILAG A or >1 new BILAG B). ^##^Composite responder index based on improvement in disease activity (at least 4 points improvement in SLEDAI-2K score) without worsening of the overall condition (no worsening in PGA) or the development of significant disease activity in new organ systems (no new BILAG A or >1 new BILAG B).

Of the included studies, 9 studies involving 5167 patients described the outcomes of SRI-4 response and comprised a total of 7 interventions based on SELENA-SLEDAI score. The network evidence map is presented in **Figure 3A**. The results of the NMA showed that Telitacicept, IL-2, Belimumab and Tabalumab had advantages in improving the SRI-4 response (**Figure 4A**). The effect of Telitacicept was significantly superior to that of Belimumab, ILT-101, Tabalumab, and Blisibimod, with the RRs with 95% CIs of 3.09 (1.64 to 5.82), 3.21 (1.15 to 8.92), 3.64 (1.88 to 7.07), and 4.08 (1.99 to 8.40), respectively. The top three drugs in the SUCRA ranking were Telitacicept (95.4%), IL-2 (82.0%), and Belimumab (56.6%) in sequence (**Supplementary Appendix 5**). In our subgroup analysis according to the administered doses, the results were consistent with those of the combined statistical analysis. The detailed results were shown in **Supplementary Appendices 6**, **7**.

Of the included studies, 17 studies involving 6852 patients reported the outcomes of SRI-4 response, including a total of 14 interventions based on SLEDAI-2K score. The network evidence map is presented in **Figure 3B**. The results of the NMA indicated that Ustekinumab, ABBV-599 high-dose, Deucravacitinib, Anifrolumab, and Sifalimumab exhibited superiority in enhancing the SRI-4 response (**Figure 4B**). Ustekinumab and Anifrolumab outperformed Epratuzumab, with RRs with 95% CIs of 2.99 (1.21 to 7.35) and 1.56 (1.05 to 2.33), respectively. Ustekinumab also showed significantly superior efficacy to Baricitinib, with the RRs with 95% CIs of 2.60 (1.07 to 6.32), respectively. The top three drugs in the SUCRA ranking were Ustekinumab (90.6%), ABBV-599 high-dose (77.1%), and Deucravacitinib (69.3%) in sequence (**Supplementary Appendix 5**). According the subgroup analysis for dosage, Ustekinumab had the highest SUCRA value (88.4%), followed by Deucravacitinib 3 mg (82.8%) and then ABBV-599 high-dose (75.8%). The detailed results were shown in **Supplementary Appendices 6**, **7**.

#### 3.2.2 BICLA response

Of all included studies, 13 studies involving 5075 patients reported the outcomes of the BICLA response, encompassing 11 interventions (**Figure 3C**). It was demonstrated that Upadacitinib, ABBV-599 high-dose, PF-04236921, Anifrolumab, and Deucravacitinib have significant advantages in improving the BICLA response (**Supplementary Appendix 4**). Upadacitinib, ABBV-599 high-dose, and Anifrolumab exhibited greater efficacy than Baricitinib, with RRs and 95% CIs of 2.95 (1.39 to 6.25), 2.44 (1.17 to 5.10), and 1.90 (1.35 to 2.67), respectively. According to the SUCRA ranking, the top three drugs were Upadacitinib (88.0%), ABBV-599 high-dose (78.5%), and PF-04236921 (71.7%) (**Supplementary Appendix 5**). Furthermore, as shown in the **Supplementary Appendices 6**, **7**, the subgroup analysis revealed that the SUCRA value of Upadacitinib 30 mg was the highest (85.9%), followed by PF-04236921–10 mg (79.3%), and then ABBV-599 high-dose (77.9%).

#### 3.2.3 CLASI-50

Ten studies, including 932 patients and 8 interventions, described the outcomes of CLASI-50, and the network evidence map is presented in **Figure 3D**. The main findings for NMA are shown in **Supplementary Appendix 4**. Deucravacitinib, Anifrolumab, and Sifalimumab exhibited superiority in enhancing the CLASI-50. Deucravacitinib and Anifrolumab were more effective than Baricitinib, with their RRs with 95% CIs of 8.93 (2.52 to 31.61) and 2.92 (1.48 to 5.75), respectively. According to the SUCRA ranking, Deucravacitinib (95.1%), Anifrolumab (66.1%), and Upadacitinib (64.8%) were likely to be more effective in improving CLASI-50 (Appendix 5). The subgroup analysis of doses showed that the SUCRA value for Deucravacitinib 3 mg was the highest (**Supplementary Appendices 6, 7**).

#### 3.2.4 LLDAS

Five studies involving 2417 patients described the outcomes of LLDAS, and the network evidence map is presented in **Figure 3E**. Significant advantages of Upadacitinib, Deucravacitinib, and ABBV-599 high-dose were observed in enhancing the LLDAS response (**Supplementary Appendix 4**). Upadacitinib and Deucravacitinib were more effective than Baricitinib, with RRs with 95% CIs of 2.73 (1.28 to 5.85) and 2.24 (1.12 to 4.51), respectively. The SUCRA rankings for the five interventions for LLDAS were as follows: Upadacitinib (88.0%), Deucravacitinib (75.3%), ABBV-599 high-dose (59.2%), Baricitinib (24.4%), and Placebo (3.1%) (**Supplementary Appendix 5**). Moreover, the subgroup analysis showed that Deucravacitinib 3mg had the highest SUCRA value (91.2%), followed by Upadacitinib (82.3%) (**Supplementary Appendices 6, 7**).

### 3.3 Safety results

#### 3.3.1 AE

Twenty studies (n=11,012; 12 interventions) reported AEs. Compared with placebo, Anifrolumab, Iberdomide, and Telitacicept were associated with a significantly higher risk of AEs, with their RRs and 95% CIs of 1.74 (1.26 to 2.40), 1.81 (1.03 to 3.15), and 2.47 (1.07 to 5.72), respectively (**Supplementary Appendix 4**). SUCRA rankings indicated that Cenerimod (89.4%), Belimumab (77.7%), and Epratuzumab (74.8%) had the most favorable safety profiles (**Supplementary Appendix 5**). Furthermore, as presented in **Supplementary Appendices 6**, **7**, Epratuzumab 100 mg had the highest SUCRA value (89.2%), followed by Cenerimod 4 mg (81.1%).

#### 3.3.2 SAEs

Twenty-two studies (n=9205; 14 interventions) reported SAEs. Compared with placebo, Belimumab was associated with a significantly lower risk of SAEs, with a RRs of 0.74 (95% CI, 0.57 to 0.97) (**Supplementary Appendix 4**). SUCRA rankings indicated that Cenerimod (86.6%), IL-2 (84.6%), and Deucravacitinib (64.4%) had the most favorable safety profiles (**Supplementary Appendix 5**). Furthermore, as presented in **Supplementary Appendices 6**, **7**, IL-2 had the highest SUCRA value (85.5%), followed by Belimumab 10 mg/kg IV (74.6%).

#### 3.3.3 Infection related AEs

Sixteen studies (n=11,978; 11 interventions) reported infection related AEs. Compared with placebo, none of the included interventions were associated with a significantly altered risk of Infection (**Supplementary Appendix 4**). SUCRA rankings indicated that IL-2 (89.3%), Epratuzumab (63.7%), and Ustekinumab (61.6%) had the most favorable safety profiles (**Supplementary Appendix 5**). Furthermore, as presented in **Supplementary Appendices 6**, **7**, IL-2 had the highest SUCRA value (89.1%), followed by Belimumab 200 mg (76.8%).

### 3.4 Assessment of inconsistency, heterogeneity, and publication bias

Inconsistency assessment was not feasible owing to the limited network structure. All analyzes were therefore performed under the consistency model. Heterogeneity tests revealed no substantial heterogeneity across studies (**Supplementary Appendix 8**). Publication bias and small-study effects were assessed using comparison-adjusted funnel plots and Egger regression tests. Most outcomes showed no significant evidence of publication bias (all *P* > 0.10). For SRI-4^#^ and LLDAS, as the limited number of studies, Egger tests was not performed correspondingly, while the sensitivity analyzes demonstrated that intervention rankings remained stable, supporting the robustness of the results (**Supplementary Appendix 8**).

## 4 Discussion

In this study, a total of 21 interventions were evaluated, including 8 small-molecule agents and 13 biologics. As far as we know, our study is the most comprehensive NMA assessing the efficacy and safety of combining biologic agents or targeted small-molecule drugs with stable background therapy for SLE. Our results demonstrated that Anifrolumab, Deucravacitinib, and ABBV-599 high-dose played significant roles in multiple efficacy indicators (SRI-4 response, BICLA response, CLASI-50, LLDAS) in the context of combined stable background therapy, with good safety profiles. According to the NMA ranking, Telitacicept and Ustekinumab were most effective for SRI-4 response, Upadacitinib for BICLA response and LLDAS achievement, with Deucravacitinib superior in CLASI-50 improvement. These findings may provide more valuable references for clinicians in the treatment of patients with SLE.

It is well acknowledged that the pathogenesis of SLE is quite complicated and involves almost all parts of the immune system. SLE is characterized by a breakdown of immune tolerance, primarily mediated by aberrant activation of self-antigen-targeted B lymphocytes and T lymphocytes (45). Both BLyS (B-cell-activating factor, BAFF) and APRIL (a proliferation-inducing ligand), members of the tumor necrosis factor (TNF) family, are critical for maintaining the B-cell pool and humoral immunity (46). Elevated BLyS levels may enhance B cell proliferation (47) and promote immunoglobulin (Ig) secretion (48), which may be associated with increased disease activity (49). Furthermore, it has been reported that cytokine dysregulation was associated with the occurrence and development of SLE, such as interleukin (IL)-12 and IL-23 (50, 51). IL-12 drives T helper 1 (Th1) and T follicular helper cell development and cytotoxic T-cell activity, while IL-23 expands pathogenic T helper 17 (Th17) cells and other IL-17-producing populations that drive tissue inflammation (27). Moreover, the type I interferon (IFN-I) pathway critically drives SLE pathogenesis by promoting plasma cell differentiation, myeloid dendritic cell activation, and elevated BAFF/APRIL expression, thereby accelerating disease progression (52–55). Additionally, IFNα was also reported to reduce lymphocyte counts in peripheral blood by promoting the migration of lymphocytes into lymph nodes, thereby leading to lymphopenia (56), and consequently affecting the progress of SLE (57).

According to our findings, it was demonstrated that Telitacicept is best for improving SRI-4 response, followed by IL-2 and then Belimumab. Telitacicept is a fusion protein that combines transmembrane activator and CAML interactor (TACI) with the Fc fragment of human IgG1 to target BLyS and APRIL, inhibits BLyS and APRIL binding to their B-cell ligands (46, 58), might thereby ameliorate SLE clinical manifestations, which is consistent with our findings. Belimumab is a human, IgG1λ monoclonal antibody that binds soluble human BlyS and inhibits its activity (59), subsequently improving clinical manifestations in SLE. Low-dose IL-2 promotes regulatory T cell (Treg) while suppressing pathogenic Th17 and Tfh populations, restoring immune tolerance in SLE (60, 61), which are required to be identified in future prospective studies. Ustekinumab and ABBV-599 high-dose showed superior efficacy on SRI-4 response relative to other comparators. Ustekinumab is a fully human monoclonal antibody that inhibits the p 40 subunit of IL-12 and IL-23 (27), thereby disrupting the IL-12 and IL-23 mediated signaling transduction and cytokine cascades, suppressing Th1/Th17-mediated immune responses, and ultimately attenuating tissue inflammation. The high-dose ABBV-599 regimen, which combines 60 mg of the Bruton’s tyrosine kinase (BTK) inhibitor Elsubrutinib with 30 mg of the Janus kinase (JAK) inhibitor Upadacitinib, was administered once daily (14). Elsubrutinib inhibits B-cell activation and immune complex-driven neutrophil activation (62), whereas Upadacitinib disrupts JAK-dependent signaling cascades—encompassing type I/II interferon receptor activation, T-cell hyperresponsiveness, and pro-inflammatory cytokine pathways (63, 64). This dual mechanism concurrently modulates divergent immunopathological pathways in SLE and thus potentially reducing disease activity. Additionally, SUCRA analysis indicated favorable efficacy rankings for both Upadacitinib and ABBV-599 high-dose regarding BICLA response and LLDAS, and Upadacitinib over the ABBV-599 high-dose. Notably, the addition of Elsubrutinib to Upadacitinib did not substantially increase the proportion of treatment responders for BICLA and LLDAS compared with patients receiving Upadacitinib alone (14), indicating that the clinical utility of BTK inhibitor-based combinations in SLE treatment requires further validation in subsequent large-scale studies. Additionally, both Deucravacitinib and Anifrolumab demonstrated superior efficacy compared with other comparators for CLASI-50. Deucravacitinib, a selective tyrosine kinase 2 (TYK2) inhibitor that binds the JH2 pseudokinase domain to block downstream signaling of IL-12, IL-23, and IFN, inhibits IFNα-2a-induced lymphopenia (57), which can alleviate inflammation and immune dysregulation, improving clinical manifestations of SLE. Anifrolumab, a fully humanized IFN-I receptor (IFNAR) inhibitor (65), targets IFNAR1 to block IFN-I signaling—reversing SLE-associated immune dysregulation and inhibiting the key pathogenic pathway in cutaneous lupus erythematosus (CLE), thereby supporting its emerging therapeutic potential for cutaneous manifestations (66, 67). Meanwhile, the oral administration method of Deucravacitinib has significant advantages in the long-term management of SLE.

We systematically evaluated the safety of 14 interventions by integrating data from both direct and indirect comparison studies, including Anifrolumab, Cenerimod, Iberdomide, Telitacicept, and Deucravacitinib. It was demonstrated that, apart from relatively higher incidence rates of AEs for Anifrolumab, Iberdomide, and Telitacicept, the safety profiles of the other interventions were comparable. Among them, Cenerimod, a potent orally active selective S1P1 receptor modulator (68), had the lowest risk of AEs and SAEs Cenerimod may become a promising strategy of SLE as the S1P1 receptor modulators can limit their migration toward inflammatory sites by inhibit lymphocyte egress from lymphoid organs (69). Consistent with the overall safety findings, no intervention was found to be associated with a statistically significant increase in the risk of infection related AEs compared with placebo in our analysis. Notably, a recent meta-analysis suggested that Anifrolumab was well tolerated and safe in the patients with SLE, but a higher risk of infections (mainly respiratory tract infections and herpes zoster) (70) was also observed. Therefore, physicians should closely monitor whether the patients show any signs or symptoms of infection when using Anifrolumab. For patients with a high risk of infection, it is recommended to conduct an infection risk assessment before the treatment and take appropriate preventive measures when available to reduce the possibility of infection.

Some limitations should be considered in our present study. Firstly, although the quality of the included studies was relatively high, the number of studies of some interventions was limited and that differed substantially across outcomes, which may limit the stability and robustness of network estimates and treatment rankings. In particular, the analysis of LLDAS was based on 5 studies. Secondly, the certainty of evidence for several main results were assessed as “Low” or “Very Low” according to the GRADE framework due to the limited number of included studies and the lack of direct comparisons of some interventions. Most included studies were placebo-controlled, with insufficient head-to-head comparisons for a comprehensive assessment of interventions. Thirdly, the patients with active severe lupus nephritis and severe CNS lupus were excluded in this present study, which limit applicability to a large and clinically important subset of SLE. Fourthly, as the limitation of the original data of included studies, further subgroup analyzes were not performed to assess the impacts of different background therapies. Therefore, our conclusions should be interpreted with due caution and these limitations should be considered within specific clinical settings, and subsequent RCTs with large-scale and high quality are required to further verified the present findings in future.

## 5 Conclusion

In conclusion, Telitacicept and ustekinumab demonstrated superior efficacy for SRI-4 response; Upadacitinib demonstrated superior efficacy for BICLA response and LLDAS achievement. Furthermore, Deucravacitinib and Anifrolumab showed significant advantages in improving CLASI-50 and may potentially serve as a promising treatment strategy for SLE patients with cutaneous manifestations. Moreover, although current findings indicate that these interventions have favorable efficacy and safety profiles, their long-term efficacy and safety still require further investigation and validation in the future. These findings will provide further evidence and reference for clinical strategies and future researches in patients with SLE.

## Data Availability

The original contributions presented in the study are included in the article/**Supplementary Material**. Further inquiries can be directed to the corresponding author.

## Supplementary material

Supplementary Figure S1 Results of network meta-analysis for BICLA response.

Supplementary Figure S2 Results of network meta-analysis for CLASI-50.

Supplementary Figure S3 Results of network meta-analysis for LLDAS.

Supplementary Figure S4 Results of network meta-analysis for AE.

Supplementary Figure S5 Results of network meta-analysis for SAE.

Supplementary Figure S6 Results of network meta-analysis for infection related adverse reactions.

Supplementary Figure S7 SUCRA ranking of SRI-4 response^#^ presented in bar chart.

Supplementary Figure S8 SUCRA ranking of SRI-4 response^##^ presented in bar chart.

Supplementary Figure S9 SUCRA ranking of BICLA response presented in bar chart.

Supplementary Figure S10 SUCRA ranking of CLASI-50 response presented in bar chart.

Supplementary Figure S11 SUCRA ranking of LLDAS response presented in bar chart.

Supplementary Figure S12 SUCRA ranking of AE response presented in bar chart.

Supplementary Figure S13 SUCRA ranking of SAE response presented in bar chart.

Supplementary Figure S14 SUCRA ranking of infection related adverse reactions response presented in bar chart.

Supplementary Figure S15 Results of network meta - analysis of treatment measures grouped by dosing for SRI-4 response^#^.

Supplementary Figure S16 Results of network meta - analysis of treatment measures grouped by dosing for SRI-4 response^##^.

Supplementary Figure S17 Results of network meta - analysis of treatment measures grouped by dosing for BICLA response.

Supplementary Figure S18 Results of network meta - analysis of treatment measures grouped by dosing for CLASI-50.

Supplementary Figure S19 Results of network meta - analysis of treatment measures grouped by dosing for LLDAS.

Supplementary Figure S20 Results of network meta - analysis of treatment measures grouped by dosing for AE.

Supplementary Figure S21 Results of network meta - analysis of treatment measures grouped by dosing for SAE.

Supplementary Figure S22 Results of network meta - analysis of treatment measures grouped by dosing for infection related adverse reactions.

Supplementary Figure S23 funnel plot. for **(A)** SRI-4 response^#^, **(B)** SRI-4 response^##^, **(C)** BICLA response, **(D)** CLASI-50, **(E)** LLDAS, **(F)** AE, **(G)** SAEs, and **(H)** infection related adverse reactions.

Supplementary Figure S24 Risk of bias summary of all included studies.

Supplementary Figure S25 Network meta-analysis results of sensitivity analysis for SRI-4 merger.

## References

[1] Ameer MA Chaudhry H Mushtaq J Khan OS Babar M Hashim T . An overview of systemic lupus erythematosus (SLE) pathogenesis, classification, and management. Cureus. (2022) 15:e30330. doi: 10.7759/cureus.30330 36407159 PMC9662848

[2] Tian J Zhang D Yao X Huang Y Lu Q . Global epidemiology of systemic lupus erythematosus: a comprehensive systematic analysis and modelling study. Ann Rheum Dis. (2023) 82:351–6. doi: 10.1136/ard-2022-223035 36241363 PMC9933169

[3] Parks CG De Souza Espindola Santos A Barbhaiya M Costenbader KH . Understanding the role of environmental factors in the development of systemic lupus erythematosus. Best Pract Res Clin Rheumatol. (2017) 31:306–20. doi: 10.1016/j.berh.2017.09.005 29224673 PMC5729939

[4] Merrill JT Ginzler EM Wallace DJ McKay JD Lisse JR Aranow C . Long-term safety profile of belimumab plus standard therapy in patients with systemic lupus erythematosus. Arthritis Rheum. (2012) 64:3364–73. doi: 10.1002/art.34564 22674457

[5] Al Hussaini M Hammouda EI Hammouda AE . Optimizing pharmacotherapy of systemic lupus erythematosus: the pharmacist role. Int J Clin Pharm. (2014) 36:684–92. doi: 10.1007/s11096-014-9966-1 24986265

[6] Idborg H Oke V . Cytokines as biomarkers in systemic lupus erythematosus: value for diagnosis and drug therapy. Int J Mol Sci. (2021) 22:11327. doi: 10.3390/ijms222111327 34768756 PMC8582965

[7] Lazar S Kahlenberg JM . Systemic lupus erythematosus: new diagnostic and therapeutic approaches. Annu Rev Med. (2023) 74:339–52. doi: 10.1146/annurev-med-043021-032611 35804480

[8] Borba HH Wiens A de Souza TT Correr CJ Pontarolo R . Efficacy and safety of biologic therapies for systemic lupus erythematosus treatment: systematic review and meta-analysis. BioDrugs. (2014) 28:211–28. doi: 10.1007/s40259-013-0074-x 24190520

[9] Wang S Ning W Tang H Mu C Huang X . Efficacy and safety study of targeted small-molecule drugs in the treatment of systemic lupus erythematosus. Arthritis Res Ther. (2024) 26:98. doi: 10.1186/s13075-024-03331-8 38730460 PMC11083747

[10] Ding Z Zhang H Huang F Liu Y Zhou Q Hu D . Efficacy and safety of biologics for systemic lupus erythematosus (SLE): a systematic review and network meta-analysis. Clin Rev Allergy Immunol. (2025) 68:70. doi: 10.1007/s12016-025-09082-x 40699272 PMC12287134

[11] Cumpston M Li T Page MJ Chandler J Welch VA Higgins JP . Updated guidance for trusted systematic reviews: a new edition of the Cochrane Handbook for Systematic Reviews of Interventions. Cochrane Database Syst Rev. (2019) 10:ED000142. doi: 10.1002/14651858.ed000142 31643080 PMC10284251

[12] Watt J Del Giovane C . Network meta-analysis. Methods Mol Biol. (2022) 2345:187–201. doi: 10.1007/978-1-0716-1566-9_12 34550592

[13] Morand E Pike M Merrill JT Van Vollenhoven R Werth VP Hobar C . Deucravacitinib, a tyrosine kinase 2 inhibitor, in systemic lupus erythematosus: a phase II, randomized, double-blind, placebo-controlled trial. Arthritis Rheumatol. (2023) 75:242–52. doi: 10.1136/annrheumdis-2022-eular.5020a PMC10100399 36369798

[14] Merrill JT Tanaka Y D’Cruz D Vila-Rivera K Siri D Zeng X . Efficacy and safety of upadacitinib or elsubrutinib alone or in combination for patients with systemic lupus erythematosus: a phase 2 randomized controlled trial. Arthritis Rheumatol. (2024) 76:1518–29. doi: 10.1002/art.42926 38923871

[15] Morand EF Vital EM Petri M van Vollenhoven R Wallace DJ Mosca M . Baricitinib for systemic lupus erythematosus: a double-blind, randomised, placebo-controlled, phase 3 trial (SLE-BRAVE-I). Lancet. (2023) 401:1001–10. doi: 10.1016/s0140-6736(22)02607-1 36848918

[16] Petri M Bruce IN Dörner T Tanaka Y Morand EF Kalunian KC . Baricitinib for systemic lupus erythematosus: a double-blind, randomised, placebo-controlled, phase 3 trial (SLE-BRAVE-II). Lancet. (2023) 401:1011–9. doi: 10.1016/s0140-6736(22)02546-6 36848919

[17] Wallace DJ Furie RA Tanaka Y Kalunian KC Mosca M Petri MA . Baricitinib for systemic lupus erythematosus: a double-blind, randomised, placebo-controlled, phase 2 trial. Lancet. (2018) 392:222–31. doi: 10.1016/s0140-6736(18)31363-1 30043749

[18] Furie RA Bruce IN Dörner T Leon MG Leszczyński P Urowitz M . Phase 2, randomized, placebo-controlled trial of dapirolizumab pegol in patients with moderate-to-severe active systemic lupus erythematosus. Rheumatol (Oxford). (2021) 60:5397–407. doi: 10.1093/rheumatology/keab381 33956056 PMC9194804

[19] Wallace DJ Stohl W Furie RA Lisse JR McKay JD Merrill JT . A phase II, randomized, double-blind, placebo-controlled, dose-ranging study of belimumab in patients with active systemic lupus erythematosus. Arthritis Rheum. (2009) 61:1168–78. doi: 10.1002/art.24699 19714604 PMC2758229

[20] Furie RA Morand EF Bruce IN Manzi S Kalunian KC Vital EM . Type I interferon inhibitor anifrolumab in active systemic lupus erythematosus (TULIP-1): a randomised, controlled, phase 3 trial. Lancet Rheumatol. (2019) 1:e208–19. doi: 10.1016/s2665-9913(19)30076-1 38229377

[21] Morand EF Furie R Tanaka Y Bruce IN Askanase AD Richez C . Trial of anifrolumab in active systemic lupus erythematosus. N Engl J Med. (2020) 382:211–21. doi: 10.1056/nejmoa1912196 31851795

[22] Navarra SV Guzmán RM Gallacher AE Hall S Levy RA Jimenez RE . Efficacy and safety of belimumab in patients with active systemic lupus erythematosus: a randomised, placebo-controlled, phase 3 trial. Lancet. (2011) 377:721–31. doi: 10.1016/s0140-6736(10)61354-2 21296403

[23] Furie R Petri M Zamani O Cervera R Wallace DJ Tegzová D . A phase III, randomized, placebo-controlled study of belimumab, a monoclonal antibody that inhibits B lymphocyte stimulator, in patients with systemic lupus erythematosus. Arthritis Rheum. (2011) 63:3918–30. doi: 10.1002/art.30613 22127708 PMC5007058

[24] Stohl W Schwarting A Okada M Scheinberg M Doria A Hammer AE . Efficacy and safety of subcutaneous belimumab in systemic lupus erythematosus: a fifty-two-week randomized, double-blind, placebo-controlled study. Arthritis Rheumatol. (2017) 69:1016–27. doi: 10.1002/art.40049 28118533 PMC5434872

[25] Zhang F Bae SC Bass D Chu M Egginton S Gordon D . A pivotal phase III, randomised, placebo-controlled study of belimumab in patients with systemic lupus erythematosus located in China, Japan and South Korea. Ann Rheum Dis. (2018) 77:355–63. doi: 10.1136/annrheumdis-2017-211631 29295825 PMC5867402

[26] Merrill JT Wallace DJ Wax S Kao A Fraser PA Chang P . Efficacy and safety of atacicept in patients with systemic lupus erythematosus: results of a twenty-four-week, multicenter, randomized, double-blind, placebo-controlled, parallel-arm, phase IIb study. Arthritis Rheumatol. (2018) 70:266–76. doi: 10.1002/art.40360 29073347 PMC6099253

[27] Van Vollenhoven RF Hahn BH Tsokos GC Wagner CL Lipsky P Touma Z . Efficacy and safety of ustekinumab, an IL-12 and IL-23 inhibitor, in patients with active systemic lupus erythematosus: results of a multicentre, double-blind, phase 2, randomised, controlled study. Lancet. (2018) 392:1330–9. doi: 10.1016/s0140-6736(18)32167-6 30249507

[28] Furie R Khamashta M Merrill JT Werth VP Kalunian K Brohawn P . Anifrolumab, an anti-interferon-α receptor monoclonal antibody, in moderate-to-severe systemic lupus erythematosus. Arthritis Rheumatol. (2017) 69:376–86. doi: 10.1016/j.berh.2017.10.005 28130918 PMC5299497

[29] Ginzler E Guedes Barbosa LS D’Cruz D Furie R Maksimowicz-McKinnon K Oates J . Phase III/IV, randomized, fifty-two-week study of the efficacy and safety of belimumab in patients of Black African ancestry with systemic lupus erythematosus. Arthritis Rheumatol. (2022) 74:112–23. doi: 10.1016/j.rdc.2014.05.004 34164944 PMC9300099

[30] Khamashta M Merrill JT Werth VP Furie R Kalunian K Illei GG . Sifalimumab, an anti-interferon-α monoclonal antibody, in moderate to severe systemic lupus erythematosus: a randomised, double-blind, placebo-controlled study. Ann Rheum Dis. (2016) 75:1909–16. doi: 10.1136/annrheumdis-2015-208562 27009916 PMC5099191

[31] Bruce IN Nami A Schwetje E Pierson ME Rouse T Chia YL . Pharmacokinetics, pharmacodynamics, and safety of subcutaneous anifrolumab in patients with systemic lupus erythematosus, active skin disease, and high type I interferon gene signature: a multicentre, randomised, double-blind, placebo-controlled, phase 2 study. Lancet Rheumatol. (2021) 3:e101–10. doi: 10.1016/s2665-9913(20)30342-8 38279367

[32] Wu D Li J Xu D Merrill JT van Vollenhoven RF Liu Y . Telitacicept in patients with active systemic lupus erythematosus: results of a phase 2b, randomised, double-blind, placebo-controlled trial. Ann Rheum Dis. (2024) 83:475–87. doi: 10.1136/ard-2023-224854 38129117 PMC10958275

[33] Hermann V Batalov A Smakotina S Juif PE Cornelisse P . First use of cenerimod, a selective S1P receptor modulator, for the treatment of SLE: a double-blind, randomised, placebo-controlled, proof-of-concept study. Lupus Sci Med. (2019) 6:e000354. doi: 10.1136/lupus-2019-000354 31798918 PMC6861098

[34] Wallace DJ Dörner T Pisetsky DS Sanchez-Guerrero J Patel AC Parsons-Rich D . Efficacy and safety of the Bruton’s tyrosine kinase inhibitor evobrutinib in systemic lupus erythematosus: results of a phase II, randomized, double-blind, placebo-controlled dose-ranging trial. ACR Open Rheumatol. (2023) 5:38–48. doi: 10.1002/acr2.11511, PMID: 36530019 PMC9837396

[35] Merrill JT Werth VP Furie R van Vollenhoven R Dörner T Petronijevic M . Phase 2 trial of iberdomide in systemic lupus erythematosus. N Engl J Med. (2022) 386:1034–45. doi: 10.1056/nejmoa2106535 35294813

[36] Tsuru T Tanaka Y Kishimoto M Saito K Yoshizawa S Takasaki Y . Safety, pharmacokinetics, and pharmacodynamics of epratuzumab in Japanese patients with moderate-to-severe systemic lupus erythematosus: results from a phase 1/2 randomized study. Mod Rheumatol. (2016) 26:87–93. doi: 10.3109/14397595.2015.1079292 26382733

[37] Clowse ME Wallace DJ Furie RA Petri MA Pike MC Leszczyński P . Efficacy and safety of epratuzumab in moderately to severely active systemic lupus erythematosus: results from two phase III randomized, double-blind, placebo-controlled trials. Arthritis Rheumatol. (2017) 69:362–75. doi: 10.1002/art.39856 27598855 PMC5299488

[38] Sheikh SZ Scheinberg MA Wei JC Tegzova D Stohl W de Toledo RA . Mortality and adverse events of special interest with intravenous belimumab for adults with active, autoantibody-positive systemic lupus erythematosus (BASE): a multicentre, double-blind, randomised, placebo-controlled, phase 4 trial. Lancet Rheumatol. (2021) 3:e122–30. doi: 10.1016/s2665-9913(20)30355-6 38279368

[39] He J Zhang R Shao M Zhao X Miao M Chen J . Efficacy and safety of low-dose IL-2 in the treatment of systemic lupus erythematosus: a randomised, double-blind, placebo-controlled trial. Ann Rheum Dis. (2020) 79:141–9. doi: 10.1136/annrheumdis-2019-215396 31537547 PMC6937406

[40] Humrich JY Cacoub P Rosenzwajg M Pitoiset F Pham HP Guidoux J . Low-dose interleukin-2 therapy in active systemic lupus erythematosus (LUPIL-2): a multicentre, double-blind, randomised and placebo-controlled phase II trial. Ann Rheum Dis. (2022) 81:1685–94. doi: 10.1136/ard-2022-222501 35973803

[41] Merrill JT Shanahan WR Scheinberg M Kalunian KC Wofsy D Martin RS . Phase III trial results with blisibimod, a selective inhibitor of B-cell activating factor, in subjects with systemic lupus erythematosus (SLE): results from a randomised, double-blind, placebo-controlled trial. Ann Rheum Dis. (2018) 77:883–9. doi: 10.1136/annrheumdis-2018-213032 29563108

[42] Isenberg DA Petri M Kalunian K Tanaka Y Urowitz MB Hoffman RW . Efficacy and safety of subcutaneous tabalumab in patients with systemic lupus erythematosus: results from ILLUMINATE-1, a 52-week, phase III, multicentre, randomised, double-blind, placebo-controlled study. Ann Rheum Dis. (2016) 75:323–31. doi: 10.1136/annrheumdis-2015-207653 26338095

[43] Tanaka Y Kumanogoh A Atsumi T Ishii T Tago F Aoki M . Safety, pharmacokinetics, biomarker response and efficacy of E6742: a dual antagonist of Toll-like receptors 7 and 8, in a first in patient, randomised, double-blind, phase I/II study in systemic lupus erythematosus. RMD Open. (2024) 10:e004701. doi: 10.1136/rmdopen-2024-004701 39289029 PMC11409405

[44] Wallace DJ Strand V Merrill JT Popa S Spindler AJ Eimon A . Efficacy and safety of an interleukin 6 monoclonal antibody for the treatment of systemic lupus erythematosus: a phase II dose-ranging randomised controlled trial. Ann Rheum Dis. (2017) 76:534–42. doi: 10.1136/annrheumdis-2016-209668 27672124 PMC5446001

[45] Kiriakidou M Ching CL . Systemic lupus erythematosus. Ann Intern Med. (2020) 172:ITC81–96. doi: 10.7326/0003-4819-159-7-201310010-01004 32479157

[46] Shi F Xue R Zhou X Shen P Wang S Yang Y . Telitacicept as a BLyS/APRIL dual inhibitor for autoimmune disease. Immunopharmacol Immunotoxicol. (2021) 43:666–73. doi: 10.1080/08923973.2021.1973493 34519594

[47] Do RK Hatada E Lee H Tourigny MR Hilbert D Chen-Kiang S . Attenuation of apoptosis underlies B lymphocyte stimulator enhancement of humoral immune response. J Exp Med. (2000) 192:953–64. doi: 10.1084/jem.192.7.953 11015437 PMC2193312

[48] Litinskiy MB Nardelli B Hilbert DM He B Schaffer A Casali P . DCs induce CD40-independent immunoglobulin class switching through BLyS and APRIL. Nat Immunol. (2002) 3:822–9. doi: 10.1038/s41590-026-02446-1 12154359 PMC4621779

[49] Petri M Stohl W Chatham W McCune WJ Chevrier M Ryel J . Association of plasma B lymphocyte stimulator levels and disease activity in systemic lupus erythematosus. Arthritis Rheum. (2008) 58:2453–9. doi: 10.1002/art.23678 18668552

[50] Dai H He F Tsokos GC Kyttaris VC . IL-23 limits the production of IL-2 and promotes autoimmunity in lupus. J Immunol. (2017) 199:903–10. doi: 10.4049/jimmunol.1700418 28646040 PMC5526729

[51] Guimarães PM Scavuzzi BM Stadtlober NP Franchi Santos LFDR Lozovoy MAB Iriyoda TMV . Cytokines in systemic lupus erythematosus: far beyond Th1/Th2 dualism lupus: cytokine profiles. Immunol Cell Biol. (2017) 95:824–31. doi: 10.1038/icb.2017.53, PMID: 28649995

[52] Lauwerys BR Ducreux J Houssiau FA . Type I interferon blockade in systemic lupus erythematosus: where do we stand? Rheumatol (Oxford). (2014) 53:1369–76. doi: 10.1093/rheumatology/ket403 24344319

[53] Crow MK Olferiev M Kirou KA . Targeting of type I interferon in systemic autoimmune diseases. Transl Res. (2015) 165:296–305. doi: 10.1016/j.trsl.2014.10.005 25468480 PMC4306610

[54] Crow MK . Type I interferon in the pathogenesis of lupus. J Immunol. (2014) 192:5459–68. doi: 10.4049/jimmunol.1002795 24907379 PMC4083591

[55] Rönnblom L Alm GV Eloranta ML . The type I interferon system in the development of lupus. Semin Immunol. (2011) 23:113–21. doi: 10.1016/j.smim.2011.01.009, PMID: 21292501

[56] Catlett IM Aras U Hansen L Liu Y Bei D Girgis IG . First-in-human study of deucravacitinib: A selective, potent, allosteric small-molecule inhibitor of tyrosine kinase 2. Clin Transl Sci. (2023) 16:151–64. doi: 10.1111/cts.13435 36325947 PMC9841305

[57] Yang Z Yu W Lu Y . Circulating lymphocyte subpopulations in patients with systemic lupus erythematosus and their correlation with disease activity. Clin Exp Med. (2023) 23:4757–63. doi: 10.1007/s10238-023-01237-4 37907622

[58] Yao X Ren Y Zhao Q Chen X Jiang J Liu D . Pharmacokinetics analysis based on target-mediated drug distribution for RC18, a novel BLyS/APRIL fusion protein to treat systemic lupus erythematosus and rheumatoid arthritis. Eur J Pharm Sci. (2021) 159:105704. doi: 10.1016/j.ejps.2021.105704 33440243

[59] Blair HA Duggan ST . Belimumab: A review in systemic lupus erythematosus. Drugs. (2018) 78:355–66. doi: 10.1007/s40265-018-0872-z 29396833

[60] He J Zhang X Wei Y Sun X Chen Y Deng J . Low-dose interleukin-2 treatment selectively modulates CD4(+) T cell subsets in patients with systemic lupus erythematosus. Nat Med. (2016) 22:991–3. doi: 10.1038/nm.4148 27500725

[61] Von Spee-Mayer C Siegert E Abdirama D Rose A Klaus A Alexander T . Low-dose interleukin-2 selectively corrects regulatory T cell defects in patients with systemic lupus erythematosus. Ann Rheum Dis. (2016) 75:1407–15. doi: 10.1136/annrheumdis-2015-207776 26324847

[62] Ding Q Zhou Y Feng Y Sun L Zhang T . Bruton’s tyrosine kinase: A promising target for treating systemic lupus erythematosus. Int Immunopharmacol. (2024) 142:113040. doi: 10.1016/j.intimp.2024.113040 39216117

[63] Tanaka Y . Systemic lupus erythematosus. Best Pract Res Clin Rheumatol. (2022) 36:101814. doi: 10.1016/j.berh.2022.101814 36702700

[64] Dörner T Tanaka Y Dow ER Koch AE Silk M Ross Terres JA . Mechanism of action of baricitinib and identification of biomarkers and key immune pathways in patients with active systemic lupus erythematosus. Ann Rheum Dis. (2022) 81:1267–72. doi: 10.1136/annrheumdis-2022-222335, PMID: 35609978 PMC9380497

[65] Peng L Oganesyan V Wu H Dall’Acqua WF Damschroder MM . Molecular basis for antagonistic activity of anifrolumab, an anti-interferon-α receptor 1 antibody. Mabs. (2015) 7:428–39. doi: 10.1080/19420862.2015.1007810 25606664 PMC4622752

[66] Patel J Borucki R Werth VP . An update on the pathogenesis of cutaneous lupus erythematosus and its role in clinical practice. Curr Rheumatol Rep. (2020) 22:69. doi: 10.1007/s11926-020-00946-z 32845411

[67] Riggs JM Hanna RN Rajan B Zerrouki K Karnell JL Sagar D . Characterisation of anifrolumab, a fully human anti-interferon receptor antagonist antibody for the treatment of systemic lupus erythematosus. Lupus Sci Med. (2018) 5:e000261. doi: 10.1136/lupus-2018-000261 29644082 PMC5890856

[68] Piali L Birker-Robaczewska M Lescop C Froidevaux S Schmitz N Morrison K . Cenerimod, a novel selective S1P receptor modulator with unique signaling properties. Pharmacol Res Perspect. (2017) 5:e00370. doi: 10.1002/prp2.370 29226621 PMC5723703

[69] Hla T Brinkmann V . Sphingosine 1-phosphate (S1P): Physiology and the effects of S1P receptor modulation. Neurology. (2011) 76:S3–8. doi: 10.1212/wnl.0b013e31820d5ec1 21339489

[70] Liu Z Cheng R Liu Y . Evaluation of anifrolumab safety in systemic lupus erythematosus: A meta-analysis and systematic review. Front Immunol. (2022) 13:996662. doi: 10.3389/fimmu.2022.996662 36211347 PMC9537685

