# Supplementary material for: Comparative effectiveness and safety of biologics and targeted small-molecule therapies plus stable background therapy in systemic lupus erythematosus: a systematic review and network meta-analysis
Source: Front Immunol. 2026 May 1;17:1739946. doi: 10.3389/fimmu.2026.1739946 (PMC13188964; doi:10.3389/fimmu.2026.1739946)
Supplement: Supplementary Figure S1 — Results of network meta-analysis for BICLA response. [file DataSheet1.pdf]

supplementary material

Appendix 1 The literature search strategy

Table S1 Search strategies for PubMed

Databases	Search number	Query	number
PubMed			
	1	((((((systemic lupus erythematosus MeSH Terms]) OR (Lupus Erythematosus Disseminatus MeSH Terms])) OR (Systemic Lupus Erythematosus MeSH Terms])) OR (Libman-Sacks Disease [MeSH Terms])) OR (Libman Sacks Disease MeSH Terms])) OR (Disease Libman-Sacks MeSH Terms])) OR (Lupus Erythematosus, Systemic MeSH Terms])	72107
	2	((Biologic Product[MeSH Terms]) OR (Product, Biologic[MeSH Terms]) OR (Products, Biological[MeSH Terms]) OR (Biologic Products MeSH Terms]) OR (Biological Product[MeSH Terms]) OR (Product, Biological[MeSH Terms]) OR (Natural Products[MeSH Terms]) OR (Natural Product[MeSH Terms]) OR (Product, Natural[MeSH Terms]) OR (Biopharmaceuticals[MeSH Terms]) OR (Biopharmaceutical[MeSH Terms]) OR (Biological[MeSH Terms]) OR (Biologic[MeSH Terms]) OR (Biological Drug[MeSH Terms]) OR (Drug, Biological[MeSH Terms]) OR (Biologic Drugs[MeSH Terms]) OR (Drugs, Biologic[MeSH Terms]) OR (Biological Drugs[MeSH Terms]) OR (Drugs, Biological[MeSH Terms]) OR (Biological Medicines[MeSH Terms]) OR (Medicines, Biological[MeSH Terms]) OR (Biologicals[MeSH Terms]) OR (Biologic Medicines[MeSH Terms]) OR (Medicines, Biologic[MeSH Terms]) OR (Biologic Pharmaceuticals[MeSH Terms]) OR (Pharmaceuticals, Biologic[MeSH Terms]) OR (Biologics[MeSH Terms]) OR (Biologic Drug[MeSH Terms]) OR (Drug, Biologic[MeSH Terms]) OR (Biological Medicine[MeSH Terms]) OR (Medicine, Biological[MeSH Terms])) OR ((((((((((biological agents[MeSH Terms]) OR (Biological Therapies[MeSH	5786003

3	Terms])) OR (Therapies, Biological[MeSH Terms])) OR (Therapy, Biological[MeSH Terms])) OR (Biologic Therapy[MeSH Terms])) OR (Biologic Therapies[MeSH Terms])) OR (Therapies, Biologic[MeSH Terms])) OR (Therapy, Biologic[MeSH Terms])) OR (Biotherapy[MeSH Terms])) OR (Biotherapies[MeSH Terms])) ((((((((((((((((((((((((((((TNF Receptor Type II-IgG Fusion Protein[MeSH Terms]) OR (TNF Receptor Type II IgG Fusion Protein Enbrel[MeSH Terms])) OR (Recombinant Human Dimeric TNF Receptor Type II-IgG Fusion Protein[MeSH Terms])) OR (TNFR-Fc Fusion Protein[MeSH Terms])) OR (Enbrel[MeSH Terms])) OR (Etanercept[MeSH Terms])) OR ((((((((Adalimumab[MeSH Terms]) OR (Humira[MeSH Terms])) OR (Amjevita[MeSH Terms])) OR (Cyltezo[MeSH Terms])) OR (D2E7 Antibody[MeSH Terms])) OR (Antibody, D2E7[MeSH Terms])) OR (Adalimumab-atto[MeSH Terms])) OR (Adalimumab-adbm[MeSH Terms]))) OR (Fenebrutinib[All Fields])) OR (cenerimod[All Fields])) OR (iberdomide[All Fields])) OR ((Certolizumab pegol[MeSH Terms]) OR (CDP 870[MeSH Terms]))) OR (((((((((CD20 Antibody, Rituximab[MeSH Terms]) OR (Rituximab CD20 Antibody[MeSH Terms])) OR (Rituxan[MeSH Terms])) OR (IDEC-C2B8 Antibody[MeSH Terms])) OR (IDEC C2B8 Antibody[MeSH Terms])) OR (IDEC-C2B8[MeSH Terms])) OR (IDEC C2B8[MeSH Terms])) OR (Mabthera[MeSH Terms])) OR (GP2013[MeSH Terms]))) OR (Ocrelizumab[All Fields])) OR (dapirolizumab[All Fields])) OR (anifrolumab[All Fields])) OR ((ustekinumab[MeSH Terms]) OR (CNTO 1275[MeSH Terms]))) OR (Epratuzumab[All Fields])) OR (abetimus[All Fields])) OR ((Edratide[All Fields]) OR (hCDR1 peptide[All Fields]))) OR (Belimumab[All Fields])) OR (Atacicept[All Fields])) OR (Evobrutinib[All Fields])) OR (((Abatacept[MeSH Terms]) OR (Orencia[MeSH Terms])) OR (BMS 188667[MeSH Terms]))) OR (Efalizumab[All Fields])) OR	75232

4	((((Sirolimus[MeSH Terms]) OR (Rapamycin[MeSH Terms])) OR (Rapamune[MeSH Terms])) OR (AY 22-989[MeSH Terms]))) OR (Infliximab[MeSH Terms])) OR (blisibimod[MeSH Terms])) OR (Sifalimumab[MeSH Terms])) OR (Mezagitamab[MeSH Terms]) ((Inhibitors, Janus Kinase[MeSH Terms]) OR (((((((((Kinase Inhibitors, Janus[Title/Abstract]) OR (Kinase Inhibitors, Janus[Title/Abstract])) OR (Inhibitor, Janus Kinase[Title/Abstract]) OR (Kinase Inhibitor, Janus[Title/Abstract]) OR (JAK Inhibitor[Title/Abstract]) OR (Inhibitor, JAK[Title/Abstract]) OR (JAK Inhibitors[Title/Abstract]) OR (Inhibitors, JAK[Title/Abstract])) OR (Tofacitinib[Title/Abstract])) OR (Solcitinib[Title/Abstract])) OR (Filgotinib[Title/Abstract])) OR (Baricitinib[Title/Abstract])) OR (Deucravacitinib[Title/Abstract])) OR (Brepocitinib[Title/Abstract]))) OR (BTK inhibitor[All Fields] OR (ibrutinib[All Fields]) OR (Orelabrutinib[All Fields]))	15704
5	((Stable background **therapy**) OR (Stable treatment)) OR (standard treatment) OR (standard of care) OR (standard of care)	1548973
6	((Randomized controlled trial) OR (RCT)) OR (Clinical trial)	1579403
7	（(#1) AND (#2 OR #3 OR #4 OR #5)） AND (#6)	1433

Table S2 Search strategies for Embase

Databases	Search number	Query	number
Embase	1	'libman-sacks disease' OR 'libman sacks disease'/exp OR 'disease libman-sacks' OR 'systemic lupus erythematosus'/exp	160760
	2	'product, biologic' OR 'products, biological' OR 'product, biological' OR 'natural products'/exp OR 'natural product'/exp OR 'product, natural' OR 'biopharmaceuticals' OR 'biopharmaceutical'/exp OR 'biological drug' OR 'drug, biological' OR 'biologic drugs' OR 'drugs, biologic' OR 'biological drugs' OR 'drugs, biological' OR 'biological medicines' OR 'medicines,	3325246

	biological' OR 'biologic medicines' OR 'medicines, biologic' OR 'biologic pharmaceuticals' OR 'pharmaceuticals, biologic' OR 'biologic drug' OR 'drug, biologic' OR 'biological medicine' OR 'medicine, biological' OR 'biological product'/exp OR 'therapies, biological' OR 'therapy, biological' OR 'therapies, biologic' OR 'therapy, biologic' OR 'biotherapy'/exp OR 'biological therapy'/exp	
3	'tnf receptor type ii-igg fusion protein' OR 'tnf receptor type ii igg fusion protein enbrel' OR 'recombinant human dimeric tnf receptor type ii-igg fusion protein' OR 'tnfr-fc fusion protein' OR 'etanercept'/exp OR 'd2e7 antibody' OR 'antibody, d2e7' OR 'adalimumab'/exp OR 'fenebrutinib'/exp OR 'cenerimod'/exp OR 'iberdomide'/exp OR 'certolizumab pegol'/exp OR 'cd20 antibody, rituximab' OR 'rituximab cd20 antibody' OR 'idec-c2b8 antibody' OR 'idec c2b8 antibody' OR 'rituximab'/exp OR 'ocrelizumab'/exp OR 'dapirolizumab' OR 'anifrolumab'/exp OR 'ustekinumab'/exp OR 'epratuzumab'/exp OR 'abetimus'/exp OR 'edratide'/exp OR 'hcdr1 peptide' OR 'belimumab'/exp OR 'atacicept'/exp OR 'evobrutinib'/exp OR 'abatacept'/exp OR 'efalizumab'/exp OR 'sirolimus'/exp OR 'ay 22-989' OR 'infliximab'/exp OR 'blisibimod'/exp OR 'sifalimumab'/exp OR 'mezagitamab'/exp	321793
4	''inhibitors, janus kinase' OR 'kinase inhibitors, janus' OR 'inhibitor, janus kinase' OR 'kinase inhibitor, janus' OR 'jak inhibitor'/exp OR 'inhibitor, jak' OR 'jak inhibitors' OR 'inhibitors, jak' OR 'tofacitinib'/exp OR 'solcitinib'/exp OR 'filgotinib'/exp OR 'baricitinib'/exp OR 'deucravacitinib'/exp OR 'brepocitinib'/exp OR 'btk inhibitor'/exp OR 'ibrutinib'/exp OR 'orelabrutinib'/exp	71180
5	(('stable background therapy') OR ('stable treatment')) OR ('standard treatment') OR ('standard of care')	215993
6	(('randomized controlled trial') OR ('rct')) OR ('clinical trial')	3168180
7	('human'/de OR 'humans':ab) AND 'adult'/de NOT 'animals'/de	11121245

	8	(#1AND (#2OR #3 OR #4 OR #5)) AND #6 AND #7	2453

Table S3 Search strategies for the Cochrane library

Databases	Search number	Query	number
The Cochrane library	#1	systemic lupus erythematosus(MeSH descriptor: [Lupus Erythematosus, Systemic] explode all trees)	1874
	#2	(Lupus Erythematosus Disseminatus):ti,ab,kw OR (Systemic Lupus Erythematosus):ti,ab,kw OR (Libman-Sacks Disease):ti,ab,kw OR (Libman Sacks Disease):ti,ab,kw OR (Disease Libman-Sacks):ti,ab,kw	3403
	#3	MeSH descriptor: [Biological Products] explode all trees	41865
	#4	(Biologic Product):ti,ab,kw OR (Product, Biologic):ti,ab,kw OR (Products, Biological):ti,ab,kw OR (Biologic Products):ti,ab,kw OR (Biological Product):ti,ab,kw OR (Product, Biological):ti,ab,kw OR (Natural Products):ti,ab,kw OR (Natural Product):ti,ab,kw OR (Product, Natural):ti,ab,kw OR (Biopharmaceuticals):ti,ab,kw OR (Biopharmaceutical):ti,ab,kw OR (Biological):ti,ab,kw OR (Biologic):ti,ab,kw OR (Biological Drug):ti,ab,kw OR (Drug, Biological):ti,ab,kw OR (Biologic Drugs):ti,ab,kw OR (Drugs, Biologic):ti,ab,kw OR (Biological Drugs):ti,ab,kw OR (Drugs, Biological):ti,ab,kw OR (Biological Medicines):ti,ab,kw OR (Medicines, Biological):ti,ab,kw OR (Biologicals):ti,ab,kw OR (Biologic Medicines):ti,ab,kw OR (Medicines, Biologic):ti,ab,kw OR (Biologic Pharmaceuticals):ti,ab,kw OR (Pharmaceuticals, Biologic):ti,ab,kw OR (Biologics):ti,ab,kw OR (Biologic Drug):ti,ab,kw OR (Drug, Biologic):ti,ab,kw OR (Biological Medicine):ti,ab,kw OR (Medicine, Biological):ti,ab,kw OR (biological agents):ti,ab,kw OR (Biological Therapies):ti,ab,kw OR (Therapies, Biological):ti,ab,kw OR (Therapy, Biological):ti,ab,kw OR (Biologic Therapy):ti,ab,kw OR (Biologic	98452

	Therapies):ti,ab,kw OR (Therapies, Biologic):ti,ab,kw OR (Therapy, Biologic):ti,ab,kw OR (Biotherapy):ti,ab,kw OR (Biotherapies):ti,ab,kw OR (TNF Receptor Type II-IgG Fusion Protein):ti,ab,kw OR (TNF Receptor Type II IgG Fusion Protein Enbrel):ti,ab,kw OR (Recombinant Human Dimeric TNF Receptor Type II-IgG Fusion Protein):ti,ab,kw OR (TNFR-Fc Fusion Protein):ti,ab,kw OR (Enbrel):ti,ab,kw OR (Etanercept) OR (Adalimumab):ti,ab,kw OR (Humira):ti,ab,kw OR (Amjevita):ti,ab,kw OR (Cyltezo):ti,ab,kw OR (D2E7 Antibody):ti,ab,kw OR (Antibody, D2E7):ti,ab,kw OR (Adalimumab-atto):ti,ab,kw OR (Adalimumab-adbm):ti,ab,kw OR (Fenebrutinib):ti,ab,kw OR (cenerimod):ti,ab,kw OR (iberdomide):ti,ab,kw OR (Certolizumab pegol):ti,ab,kw OR (CDP 870):ti,ab,kw OR (CD20 Antibody, Rituximab):ti,ab,kw OR (Rituximab CD20 Antibody):ti,ab,kw OR (Rituxan):ti,ab,kw OR (IDEC-C2B8 Antibody):ti,ab,kw OR (IDEC C2B8 Antibody):ti,ab,kw OR (IDEC-C2B8):ti,ab,kw OR (IDEC C2B8):ti,ab,kw OR (Mabthera):ti,ab,kw OR (GP2013):ti,ab,kw OR (Ocrelizumab):ti,ab,kw OR (dapirolizumab):ti,ab,kw OR (anifrolumab):ti,ab,kw OR (ustekinumab):ti,ab,kw OR (CNTO 1275):ti,ab,kw OR (Epratuzumab):ti,ab,kw OR (abetimus):ti,ab,kw OR (Edratide):ti,ab,kw OR (hCDR1 peptide):ti,ab,kw OR (Belimumab):ti,ab,kw OR (Atacicept):ti,ab,kw OR (Evobrutinib):ti,ab,kw OR (Abatacept):ti,ab,kw OR (Orencia):ti,ab,kw OR (BMS 188667):ti,ab,kw OR (Efalizumab):ti,ab,kw OR (Sirolimus):ti,ab,kw OR (Rapamycin):ti,ab,kw OR (Rapamune):ti,ab,kw OR (AY 22 989):ti,ab,kw OR (Infliximab):ti,ab,kw OR (blisibimod):ti,ab,kw OR (Sifalimumab):ti,ab,kw OR (Mezagitamab):ti,ab,kw	
#5	MeSH descriptor: [Janus Kinase Inhibitors] explode all trees	356
#6	(Inhibitors, Janus Kinase):ti,ab,kw OR (Kinase Inhibitors, Janus):ti,ab,kw OR (Kinase Inhibitors, Janus):ti,ab,kw OR (Inhibitor, Janus Kinase):ti,ab,kw OR (Kinase Inhibitor, Janus):ti,ab,kw OR (JAK Inhibitor):ti,ab,kw OR (Inhibitor, JAK):ti,ab,kw OR (JAK Inhibitors):ti,ab,kw OR (Inhibitors, JAK):ti,ab,kw	4251

	OR (Tofacitinib):ti,ab,kw OR (Solcitinib):ti,ab,kw OR (Filgotinib):ti,ab,kw OR (Baricitinib):ti,ab,kw OR (Deucravacitinib):ti,ab,kw OR (Brepocitinib):ti,ab,kw	
#7	MeSH descriptor: [Molecular Targeted Therapy] explode all trees	451
#8	(BTK inhibitor):ti,ab,kw OR (ibrutinib):ti,ab,kw OR (Orelabrutinib):ti,ab,kw	2541
#9	(Stable background therapy):ti,ab,kw OR (Stable treatment):ti,ab,kw OR (standard treatment):ti,ab,kw	204798
#10	(Randomized controlled trial):ti,ab,kw OR (RCT):ti,ab,kw OR (Clinical trial):ti,ab,kw	997574
#11	((#1 OR #2) AND (#3 OR #4 OR #5 OR #6 OR #7 OR #8 OR #9)) AND (#10)	976

Appendix 2 Description of Efficacy Measures

Table S4 Description of Efficacy Measures.

Measure	Definition	Reference
Systemic Lupus Erythematosus Responder Index-4 (SRI-4)	Composite responder index based on improvement in disease activity (at least 4 point improvement in SELENA-SLEDAI or SLEDAI-2K score) without worsening of the overall condition (no worsening in PGA) or the development of significant disease activity in new organ systems (no new BILAG A or >1 new BILAG B).	(1), (2), (3)
BILAG-based Composite Lupus Assessment response (BICLA)	BILAG-based Combined Lupus Assessment (BICLA) response was defined as improvement in all baseline BILAG A and B scores (and ≤1 new BILAG B score) without worsening in PhGA (<0.3-point increase) or SLEDAI-2K score.	(4)
Cutaneous Lupus Erythematosus Disease Area and Severity Index 50 (CLASI-50)	Cutaneous Lupus Erythematosus Disease Area and Severity Index (CLASI) is an indicator to evaluate the severity of cutaneous lupus erythematosus, and the score ranges from 0 to 70, with a higher score indicating more severe condition and higher activity, and a 50% reduction in the CLASI-A score called CLASI-50	(5)
Lupus Low Disease Activity State (LLDAS)	A state of low disease activity based on SLEDAI score, absence of SLE disease activity in major organ systems and new disease activity, Physician’s Global Assessment, and concomitant medication usage.	(6)

Reference

1. Furie RA, Petri MA, Wallace DJ, Ginzler EM, Merrill JT, Stohl W, et al. Novel evidence-based systemic lupus erythematosus responder index. Arthritis Rheum 2009; 15;61(9):1143-51.

2. American College of Rheumatology Ad Hoc Committee on Systemic Lupus Erythematosus Response Criteria. The American College of Rheumatology response criteria for systemic lupus erythematosus clinical trials: measures of overall disease activity. Arthritis Rheum 2004; 50(11):3418-26.

3. Luijten KM, Tekstra J, Bijlsma JW, Bijl M. The Systemic Lupus Erythematosus Responder Index (SRI); a new

SLE disease activity assessment. Autoimmun Rev 2012; 11(5):326-9.

4. Wallace DJ, Kalunian K, Petri MA, Strand V, Houssiau FA, Pike M, et al. Efficacy and safety of epratuzumab in patients with moderate/severe active systemic lupus erythematosus: results from EMBLEM, a phase IIb, randomised, double-blind, placebo-controlled, multicentre study. Ann Rheum Dis 2014; 73(1):183-90.

5. Albrecht J, Taylor L, Berlin JA, Dulay S, Ang G, Fakharzadeh S, et al. The CLASI (Cutaneous Lupus Erythematosus Disease Area and Severity Index): an outcome instrument for cutaneous lupus erythematosus. J Invest Dermatol 2005; 125(5):889-94.

6. Franklyn K, Lau CS, Navarra SV, Louthrenoo W, Lateef A, Hamijoyo L, et al. Definition and initial validation of a Lupus Low Disease Activity State (LLDAS). Ann Rheum Dis 2016; 75(9):1615-21.

Appendix 3

Table S5: Medication Profiles and Ethnic Information of Patients in Included Studies

Study	Interventions	Baseline treatment for SLE						Race, n (%)				
		Glucocorticoid use, n (%)	Mean prednisone dose(or equivalent), Mean± SD mg/day	≥10 mg/day prednisone or equivalent, n (%)	Antimalarial agent, n (%)	Immunosuppressant agent, n (%)	Non-steroidal anti-inflammatory drug, n (%)	American Indian or Alaska Native	Asian	Black or African American	White	Other
Eric Morand, 2023	deucravacitinib 3mg;deucravacitinib,6mg; deucravacitinib,12mg; placebo	74(81.3)	NR	45(49.5)	81 (89.0)	53 (58.2)	NR	3 (3.3)	9 (9.9)	10(11.0)	62(68.1)	7 (7.7)
		73(78.5)		46(49.5)	84 (90.3)	43 (46.2)		5 (5.4)	15(16.1)	8 (8.6)	55(59.1)	10 (10.8)
		71(79.8)		43(48.3)	75 (84.3)	46 (51.7)		2 (2.2)	10(11.2)	9 (10.1)	57(64.0)	11 (12.4)
		74(82.2)		47(52.2)	75 (83.3)	46 (51.1)		4 (4.4)	10(11.1)	6 (6.7)	60(66.7)	10 (11.1)
Eric F Morand, 2023	Placebo, baricitinib 4mg, baricitinib 2mg	195 (77)	9.8±5	112 (44)	213 (84)	150 (59)	63 (25)	12 (5)	33 (13)	36 (14)	168(67)	NR
		194 (76)	10.4±7	102 (40)	189 (74)	152 (60)	67 (26)	15 (6)	39 (16)	23 (9)	172 (69)	
		187 (74)	10.1±6	105 (42)	206 (82)	141 (56)	68 (27)	7 (3)	34 (14)	30 (12)	177 (71)	

Michelle Petri, 2023	Placebo; baricitinib 4mg; baricitinib 2mg	207 (81)	8.8±5.0	101 (39)	209 (82)	140 (55)	51 (20)	14 (6)	71 (28)	17 (7)	145 (58)	NR
		210 (80)	9.6±5.9	113 (43)	213 (82)	133 (51)	64 (25)	12 (5)	66 (26)	23 (9)	152 (59)	
		207 (80)	9.8±5.7	104 (40)	213 (83)	133 (52)	70 (27)	13 (5)	70 (28)	26 (10)	140 (55)	
Daniel J Wallace, 2018	Placebo; baricitinib 4mg; baricitinib 2mg	77 (73)	7.9±4.6	NR	75 (71)	45 (43)	27 (26)	9 (8.6)	20(19.0)	5 (4.8)	71(67.6)	NR
		79 (75)	8.7±5.8		71 (68)	47 (45)	29 (28)	6 (5.8)	20(19.2)	9 (8.7)	68(65.4)	
		74 (71)	10.5±17.4		76 (73)	50 (48)	32 (31)	10 (9.7)	20(19.4)	7 (6.8)	65(63.1)	
Joan T Merrill, 2024	upadacitinib+elsubrutinib (ABBV-599 high-dose);Upadacitinib 30mg; Placebo	52(76.5)	6.7±6.4	12(23.1)	62 (91.2)	26 (38.2)	NR	NR	14 (20.6)	4 (5.9)	45(66.2)	3 (4.4)
		42(67.7)	6.2±6.1	11(26.2)	51 (82.3)	25 (40.3)			13(21.0)	9 (14.5)	34(54.8)	0
		56(74.7)	7.9±7.1	16(28.6)	67 (89.3)	28 (37.3)			23(30.7)	4 (5.3)	43(57.3)	3 (4.0)
Richard A Furie, 2021	Placebo;dapirolizumab pegol 6mg/kg;dapirolizumab pegol 24mg/kg;dapirolizumab pegol 45mg/kg;	38(88.4)	median (min–max) 10.0 (0.0–40.0) 10.0 (0.0–25.0) 10.0 (0.0–20.0)	27(62.8)	29 (67.4)	22 (48.9)	NR	2 (4.7)	1 (2.3)	1 (2.3)	25(58.1)	14 (32.6)
		40(93.0)		28(65.1)	30 (69.8)	25 (55.6)		1 (2.3)	0	4 (9.3)	24(55.8)	14 (32.6)
		39(88.6)		24(54.5)	33 (75.0)	25 (55.6)		1 (2.3)	1 (2.3)	1 (2.3)	32(72.7)	9 (20.5)
		36(78.3)		24(52.2)	28 (60.9)	26 (55.3)		1 (2.2)	0	5 (10.9)	27(58.7)	13 (28.3)

			10.0 (0.0–<br>30.0)									
Daniel J<br>Wallace,<br>2009	Placebo; Belimumab<br>1mg/kg; Belimumab<br>4mg/kg;Belimumab<br>10mg/kg	82(72.6)<br>78(68.4)<br>73(65.8)<br>74(66.7)	NR	NR	84(74.3)<br>80(70.2)<br>72(64.9)<br>77(69.4)	55(48.7) 52(45.6)<br>59(53.2) 58(52.3)	NR	23(20.4)<br>24(21.1)<br>31(27.9)<br>28(25.2)	NR	NR	NR	NR
Richard A<br>Furie,<br>2019	Placebo;Anifrolumab<br>300mg	153 (83)<br>150 (83)	11.9±7.7<br>12.8±12.0	102 (55)<br>103 (57)	134 (73)<br>124 (69)	AZA 34(18) 32(18)<br>MTX 38(21) 22(12)<br>MMF 22(12) 31(17)	35 (19)<br>31 (17)	23 (13)<br>29 (16)	5 (3)<br>11 (6)	NR	137 (74)<br>125 (69)	18 (10)<br>15 (8)
Eric F<br>Morand,<br>2020	Placebo;Anifrolumab<br>300mg	151 (83.0)<br>141 (78.3)	NR	NR	133 (73.1)<br>119 (66.1)	86 (47.3)<br>88 (48.9)	NR	NR	30(16.5)<br>30(16.7)	25(13.7)<br>17 (9.4)	107(58.8)<br>110(61.1)	20 (11.0)<br>23 (12.8)
Sandra V<br>Navarra,<br>2011	Placebo; Belimumab<br>1mg/kg; Belimumab<br>10mg/kg	276 (96)<br>276 (96)<br>278 (96)	11.9±7.9<br>12.9±8.6<br>13.2±9.5	NR	201 (70)<br>195 (68)<br>185 (64)	122 (43)<br>120 (42)<br>123 (42)	NR	NR	NR	NR	NR	NR

Richard Furie, 2011	Placebo; Belimumab 1mg/kg; Belimumab 10mg/kg	212 (77.1)<br>211(77.9)<br>200(73.3)	9.4±8.9<br>8.7±7.6<br>8.4±7.9	NR	180 (65.5)<br>171 (63.1)<br>168 (61.5)	154 (56.0)<br>153 (56.5)<br>148 (54.2)	NR	NR	11 (4.0)<br>6 (2.2)<br>11 (4.0)	39(14.2)<br>40(14.8)<br>39(14.3)	188(68.4)<br>192(70.8)<br>189(69.2)	NR
William Stohl, 2017	Placebo; Belimumab 200mg SC	31(11.1)<br>59(10.6)	NR	NR	16 (5.7)<br>44 (7.9)	7 (2.5)<br>10 (1.8)	NR	NR	NR	NR	NR	NR
Fengchun Zhang, 2018	Placebo; Belimumab 10mg/kg	223(98.7)<br>443(98.2)	NR	NR	157 (69.5)<br>320 (71.0)	146 (64.6)<br>292 (64.7)	NR	NR	NR	NR	NR	NR
Joan T Merrill, 2018a	Placebo;Atacicept 75mg;Atacicept 150mg	NR	9.40±7.503;10 .18±8.898;9.4 1±7.417	NR	78 (78.0)<br>75 (73.5)<br>80 (76.9)	AZA 20 (20.0) 20 (19.6) 21 (20.2) MTX 18 (18.0) 12 (11.8) 13 (12.5) Mycophenolate 16 (16.0) 16 (15.7) 18 (17.3)	NR	4 (4.0)<br>3 (2.9)<br>4 (3.8)	7 (7.0)<br>15(14.7)<br>14(13.5)	5 (5.0)<br>6 (5.9)<br>9 (8.7)	78(78.0)<br>72(70.6)<br>66(63.5)	5 (5.0)<br>6 (5.9)<br>11 (10.6)
Ronald F van	Ustekinumab;Placebo	51 (85)<br>34 (81)	9.3±4.7;<br>10.5±5.2	NR	44 (73)<br>26 (62)	MMF8(13)7(17) AZAor 6-	NR	NR	8 (13)<br>6 (14)	4 (7)<br>3 (7)	42 (70)<br>28 (67)	6 (10)<br>5 (12)

Vollenhoven, 2018						MP12(20)9(21)MTX12(20)6(14)						
Richard Furie, 2017	Placebo;Anifrolumab 300mg;Anifrolumab 1000mg	88(86.3)<br>79(79.8)<br>91(87.5)	12.8±8.1<br>11.3±6.4<br>12.5±7.8	NR	75 (73.5)<br>76 (76.8)<br>68 (65.4)	AZA19(18.6)23(23.2)21(20.2)MTX16(15.7)19(19.2)25(24.0)Mycophenolate 11(10.8)11(11.1)11(10.6)	NR	0 (0.0)<br>4 (4.0)<br>1 (1.0)	13(12.7)<br>3 (3.0)<br>6 (5.8)	12(11.8)<br>19(19.2)<br>10 (9.6)	41(40.2)<br>35(35.4)<br>51(49.0)	36 (35.3)<br>38 (38.4)<br>36 (34.6)
Ellen Ginzler, 2022	Belimumab 10mg/kg;<br>Placebo	246(82.3)<br>127(85.2)	NR	NR	237 (79.3)<br>124 (83.2)	167 (55.9)<br>88 (59.1)	62 (20.7)<br>20 (13.4)	NR	NR	293(98.0)<br>143 (96.0)	NR	NR
Munther Khamashta, 2016	Placebo;Sifalimumab 200mg;Sifalimumab 600mg;Sifalimumab 1200mg	93(86.1)<br>96(88.9)<br>87(80.6)<br>92(86.0)	11.1±5.5<br>10.9±5.5<br>10.8±5.7<br>11.6±5.8	NR	77 (71.3)<br>77 (71.3)<br>83 (76.9)<br>79 (73.8)	AZA28(25.9)31(28.7)31(28.7)21(19.6)MTX14(13.0)17(15.7)17(15.7)16(15.0)Mycophenolate13(12.0)11 (10.2)5(4.6)12 (11.2)	NR	8 (7.4)<br>4 (3.7)<br>3 (2.8)<br>5 (4.7)	19(17.6)<br>14(13.0)<br>16(14.8)<br>16(15.0)	7 (6.5)<br>8 (7.4)<br>7 (6.5)<br>11(10.3)	63(58.3)<br>67(62.0)<br>67(62.0)<br>56(52.3)	11 (10.2)<br>15 (13.9)<br>15 (13.9)<br>19 (17.8)
Ian N Bruce, 2021	Placebo;Anifrolumab 150mg;Anifrolumab 300mg	7 (78) 12 (86) 11 (85)	10.3±7.6;<br>7.0±3.9;<br>9.6±6.9;	7 (78) 6 (43) 6 (46)	7 (78) 12 (86) 8 (62)	AZA1(11)4(29)2(15)MTX1(11)2(14)3(23)MPA1(11)2(14)1(8)	NR	NR	NR	NR	NR	NR

Di Wu, 2024	Placebo; Telitacicept 240mg; Telitacicept 160mg; Telitacicept 80mg;	NR	16.07±11.61;18.59±13.14;14.20±9.42;18.71±13.05	NR	NR	NR	NR	NR	NR	NR	NR	NR
Viktoria Hermann, 2019	Placebo; Cenerimod 0.5mg; Cenerimod 1mg; Cenerimod 2mg; Cenerimod 4mg;	NR	NR	NR	NR	NR	NR	NR	NR	NR	15(88.2)<br>12 (100)<br>12 (100)<br>13 (100)<br>13 (100)	NR
Daniel J. Wallace, 2023	Placebo;Evobrutinib 25mg QD;Evobrutinib 75mg QD;Evobrutinib 50mg BID;	NR	8.69±6.1<br>9.00±7.1<br>8.74±6.9<br>8.94±6.4	NR	90 (76.9)<br>88 (74.6)<br>95 (81.2)<br>89 (76.1)	AZA20(17.1)17(14.4)20(17.1)23(19.7)MTX14(120)11(9.3)25(21.4)15(12.8)MMFor MPS14(120)23(19.5)9(7.7)15(12.8)LEF0(0.0)1(0.8)3(2.6)1(0.9)TAC0(0.0)1(0.8)0(0.0)1(0.9)CSA0(0.0	NR	NR	23 (19.7)<br>17 (14.4)<br>21 (17.9)<br>13 (11.1)	10 (8.5)<br>12 (10.2)<br>11 (9.4)<br>12 (10.3)	66 (56.4)<br>73 (61.9)<br>68 (58.1)<br>83 (70.9)	18 (15.4)<br>16 (13.6)<br>17 (14.5)<br>9 (7.7)

)0(0.0)1(0.9)0 (0.0)

Joan T. Merrill, 2022	Iberdomide, 0.45mg<br>Iberdomide, 0.30mg;<br>Iberdomide 0.15mg;<br>Placebo;	58 (72)<br>64 (78)<br>31 (74)<br>64 (77)	NR	32 (40)<br>30 (37)<br>17 (40)<br>31 (37)	50 (62)<br>63 (77)<br>28 (67)<br>66 (80)	37 (46)<br>36 (44)<br>22 (52)<br>34 (41)	NR	NR	NR	5 (6)<br>6 (7)<br>3 (7)<br>7 (8)	60 (74)<br>59 (72)<br>29 (69)<br>60 (72)	16 (20)<br>17 (21)<br>10 (24)<br>16 (19)
Tomomi Tsuru, 2016	Placebo;Epratuzumab 100mg Q2W; Epratuzumab 400mg Q2W; Epratuzumab 600mg QW;Epratuzumab 1200mg Q2W	NR	18.1±12.5<br>12.5±6.5<br>15.4±10.3<br>11.9±5.5<br>13.1±11.8	NR	0 (0)<br>0 (0)<br>0 (0)<br>0 (0)<br>0 (0)	0 (0)<br>1 (25.0)<br>1 (25.0)<br>2 (50.0)<br>0 0	NR	NR	NR	NR	NR	NR
Megan E B Clowse, 2017a	Placebo;epratuzumab 1,200mg QOW;epratuzumab 600mg QW;	248(99.6)<br>241(98.8)<br>247(99.6)	NR	NR	175 (70.3)<br>181 (74.2)<br>181 (73.0)	116 (46.6)<br>123 (50.4)<br>112 (45.2)	NR	NR	26(10.4)<br>22 (9.0)<br>18 (7.3)	26(10.4)<br>32(13.1)<br>33(13.3)	187(75.1)<br>178(73.0)<br>188(75.8)	NR

Megan E B Clowse, 2017b	Placebo;epratuzumab 1,200mg QOW; epratuzumab 600mg QW;	256(97.3);<br>257(98.5);<br>263(99.6)	NR	NR	162 (61.6)<br>160 (61.3)<br>179 (67.8	121 (46.0)<br>113 (43.3)<br>129 (48.9)	NR	NR	7 (2.7) 7 (2.7) 12 (4.5)	25 (9.5)<br>29(11.1)<br>34(12.9)	204(77.6)<br>198(75.9)<br>193(73.1)	NR
Saira Z Sheikh, 2021	Belimumab; Placebo	NR	NR	NR	NR	NR	NR	NR	NR	NR	NR	NR
Jing He, 2020	IL-2; Placebo	NR	12.5 (0–50)<br>15 (5–50)	NR	29 (96.67)<br>28 (93.33)	CTX4(13.33)0(0)AZA1(3.33)4(13.33)CSA0(0)5(16.67)MMF9(30.00)8(26.67)TAC1(3.33)1(3.33)LEF3(10.00)1(3.33)THD1(3.33)0(0)MTX1(3.33)1(3.33)	NR	NR	NR	NR	NR	NR
Jens Y Humrich, 2022	ILT 101; Placebo	NR	10.9±4.2<br>11.2±6.7	NR	38 (76)<br>39 (78)	25 (50)<br>20 (40)	NR	NR	1 (2)<br>1 (2)	2 (4)<br>0 (0)	35 (70)<br>39 (78)	NR
Joan T Merrill,	Blisibimod 200mg QW; Placebo	NR	15.6±8.58;15.6±9.75	NR	150 (61.2)<br>122 (62.2)	104 (42.4)<br>82 (41.8)	NR	NR	67 (27.3)<br>60(30.6)	10 (4.1)<br>4 (2.0)	167 (68.2)<br>132(67.3)	NR

2018b												
D A Isenberg, 2015	Tabalumab 120mg Q2W;<br>Tabalumab120mg Q4W;<br>Placebo	300(78.7)<br>296(78.3)<br>295(77.8)	11.9±7.4<br>12.1±7.8<br>12.1±7.9	NR	241 (63.3)<br>241 (63.8)<br>243 (64.1)	158 (41.5)<br>176 (46.6)<br>164 (43.3)	NR	65 (17.1)<br>55 (14.6)<br>67 (17.7)	68(17.8)<br>61(16.1)<br>66(17.4)	40(10.5)<br>41(10.8)<br>39(10.3)	204(53.5)<br>218(57.7)<br>205(54.1)	NR
Yoshiya Tanaka, 2024	Placebo; E6742 100mg; E6742 200mg	NR	4.8±3.45<br>5.6±2.15<br>6.2±2.99	1 (11.1)<br>0<br>0	8 (88.9)<br>7 (87.5)<br>6 (66.7)	4 (44.4)<br>4 (50.0)<br>4 (44.4)	NR	NR	NR	NR	NR	NR
Daniel J Wallace, 2017	Placebo;PF04236921 10mg; PF04236921 50 mg	31(68.9)<br>32(71.1)<br>36(76.6)	NR	NR	34 (75.6)<br>35 (77.8)<br>34 (72.3)	20 (44.4)<br>18 (40.0)<br>21 (44.7)	NR	NR	1 (2.2)<br>1 (2.2)<br>0 (0.0)	4 (8.9)<br>3 (6.7)<br>8 (17.0)	33(73.3)<br>37(82.2)<br>36(76.6)	7 (15.6)<br>4 (8.9)<br>3 (6.4)

Abbreviations: AZA: azathioprine, MTX: methotrexate, MMF: mycophenolate mofetil, Mycophenolate Sodium: MPS, mycophenolic acid: MPA, 6-mercaptopurine: 6-MP, leflunomide: LEF, Tacrolimus: TAC, Cyclosporin: CSA, Cyclophosphamide: CTX, Thalidomide: THD, NR: not reported.

Appendix 4 Results of network meta-analysis

**Upadacitinib**										
1.21 (0.61,2.40)	**ABBV-599 high-dose**									
1.37 (0.46,4.12)	1.14 (0.38,3.38)	**PF04236921**								
1.55 (0.72,3.35)	1.29 (0.61,2.73)	1.13 (0.47,2.71)	**Anifrolumab**							
1.76 (0.72,4.31)	1.46 (0.60,3.53)	1.28 (0.48,3.44)	1.13 (0.63,2.05)	**Deucravacitinib**						
1.84 (0.67,5.06)	1.53 (0.56,4.14)	1.35 (0.45,4.00)	1.19 (0.56,2.52)	1.05 (0.43,2.54)	**Dapirolizumab pegol**					
1.92 (0.30,12.34)	1.59 (0.25,10.16)	1.40 (0.21,9.42)	1.23 (0.22,7.01)	1.09 (0.18,6.58)	1.04 (0.16,6.66)	**E6742**				
**2.95 (1.39,6.25)**	**2.44 (1.17,5.10)**	2.15 (0.91,5.07)	**1.90 (1.35,2.67)**	1.68 (0.94,2.97)	1.60 (0.76,3.34)	1.54 (0.27,8.68)	**Baricitinib**			
**2.97 (1.40,6.31)**	**2.46 (1.17,5.15)**	2.17 (0.92,5.12)	**1.91 (1.35,2.70)**	1.69 (0.95,3.00)	1.61 (0.77,3.37)	1.55 (0.27,8.75)	1.01 (0.74,1.37)	**Epratuzumab**		
**3.11 (1.04,9.37)**	2.58 (0.87,7.68)	2.27 (0.70,7.37)	2.01 (0.84,4.80)	1.77 (0.66,4.76)	1.69 (0.57,5.03)	1.63 (0.24,10.96)	1.06 (0.45,2.50)	1.05 (0.44,2.49)	**Ustekinumab**	
**3.35 (1.63,6.90)**	**2.78 (1.37,5.62)**	**2.45 (1.07,5.62)**	**2.16 (1.66,2.81)**	**1.91 (1.12,3.25)**	1.82 (0.90,3.68)	1.75 (0.31,9.75)	1.14 (0.92,1.41)	1.13 (0.90,1.41)	1.08 (0.47,2.48)	**Placebo**

Figure S1 Results of network meta-analysis for BICLA response

**Deucravacitinib**							
3.06 (0.87,10.81)	**Anifrolumab**						
2.76 (0.32,23.60)	0.90 (0.14,5.81)	**Upadacitinib**					
3.55 (0.86,14.58)	1.16 (0.46,2.93)	1.29 (0.18,9.21)	**Sifalimumab**				
4.60 (0.65,32.61)	1.50 (0.29,7.77)	1.67 (0.27,10.33)	1.30 (0.22,7.55)	**ABBV-599 high-dose**			
**5.42 (1.06,27.70)**	1.77 (0.52,6.08)	1.97 (0.23,16.55)	1.53 (0.38,6.13)	1.18 (0.17,8.22)	**Iberdomide**		
**8.28 (2.57,26.62)**	**2.70 (1.68,4.36)**	3.00 (0.50,18.17)	**2.33 (1.05,5.17)**	1.80 (0.37,8.68)	1.53 (0.49,4.76)	**Placebo**	
**8.93 (2.52,31.61)**	**2.92 (1.48,5.75)**	3.24 (0.50,20.89)	2.52 (0.99,6.38)	1.94 (0.37,10.07)	1.65 (0.48,5.67)	1.08 (0.67,1.75)	**Baricitinib**

Figure S2 Results of network meta-analysis for CLASI-50

**Upadacitinib**				
1.22 (0.46,3.26)	**Deucravacitinib**			
1.52 (0.76,3.04)	1.25 (0.47,3.31)	**ABBV-599 high-dose**		
**2.73 (1.28,5.85)**	**2.24 (1.12,4.51)**	1.80 (0.85,3.82)	**Baricitinib**	
**3.17 (1.53,6.55)**	**2.60 (1.34,5.04)**	**2.09 (1.02,4.28)**	1.16 (0.93,1.45)	**Placebo**

Figure S3 Results of network meta-analysis for LLDAS

Cenerimod	Belimumab	Epratuzumab	Placebo	Deucravacitinib	Sifalimumab	Anifrolumab	Ustekinumab	Iberdomide	IL-2	ILT-101	Telitacicept
Cenerimod	1.82 (0.59,5.60)	0.85 (0.03,23.90)	1.97 (0.65,6.03)	2.07 (0.54,7.86)	2.34 (0.64,8.57)	**3.43 (1.07,10.97)**	3.57 (0.86,14.86)	**3.56 (1.02,12.41)**	3.84 (0.82,17.95)	**4.66 (1.13,19.29)**	**4.88 (1.21,19.72)**
0.55 (0.18,1.69)	Belimumab	0.47 (0.02,10.87)	1.08 (0.95,1.24)	1.14 (0.54,2.39)	1.29 (0.66,2.53)	**1.89 (1.33,2.67)**	1.96 (0.80,4.82)	**1.96 (1.10,3.47)**	2.11 (0.72,6.17)	**2.56 (1.06,6.22)**	**2.68 (1.15,6.27)**
1.18 (0.04,33.41)	2.15 (0.09,50.28)	Epratuzumab	2.33 (0.10,54.38)	2.45 (0.10,62.00)	2.77 (0.11,69.13)	4.06 (0.17,96.11)	4.22 (0.16,111.14)	4.21 (0.17,103.08)	4.54 (0.16,125.96)	5.51 (0.21,144.86)	5.77 (0.22,150.08)
0.51 (0.17,1.55)	0.92 (0.80,1.06)	0.43 (0.02,9.99)	Placebo	1.05 (0.51,2.18)	1.19 (0.61,2.30)	**1.74 (1.26,2.40)**	1.81 (0.74,4.39)	**1.81 (1.03,3.15)**	1.94 (0.67,5.64)	2.36 (0.98,5.68)	**2.47 (1.07,5.72)**
0.48 (0.13,1.84)	0.88 (0.42,1.85)	0.41 (0.02,10.36)	0.95 (0.46,1.98)	Deucravacitinib	1.13 (0.42,3.03)	1.66 (0.75,3.68)	1.72 (0.55,5.44)	1.72 (0.69,4.31)	1.85 (0.51,6.74)	2.25 (0.72,7.05)	2.36 (0.78,7.17)
0.43 (0.12,1.56)	0.78 (0.40,1.52)	0.36 (0.01,9.01)	0.84 (0.44,1.63)	0.88 (0.33,2.36)	Sifalimumab	1.46 (0.70,3.05)	1.52 (0.50,4.60)	1.52 (0.64,3.60)	1.64 (0.47,5.73)	1.99 (0.66,5.96)	2.08 (0.72,6.05)
**0.29 (0.09,0.93)**	**0.53 (0.37,0.75)**	0.25 (0.01,5.84)	**0.58 (0.42,0.79)**	0.60 (0.27,1.34)	0.68 (0.33,1.42)	Anifrolumab	1.04 (0.40,2.67)	1.04 (0.55,1.97)	1.12 (0.37,3.40)	1.36 (0.53,3.46)	1.42 (0.58,3.49)
0.28 (0.07,1.17)	0.51 (0.21,1.25)	0.24 (0.01,6.25)	0.55 (0.23,1.34)	0.58 (0.18,1.83)	0.66 (0.22,1.99)	0.96 (0.37,2.47)	Ustekinumab	1.00 (0.35,2.85)	1.08 (0.27,4.30)	1.31 (0.38,4.55)	1.37 (0.40,4.64)
**0.28 (0.08,0.98)**	**0.51 (0.29,0.91)**	0.24 (0.01,5.81)	**0.55 (0.32,0.97)**	0.58 (0.23,1.46)	0.66 (0.28,1.56)	0.96 (0.51,1.83)	1.00 (0.35,2.86)	Iberdomide	1.08 (0.32,3.58)	1.31 (0.46,3.70)	1.37 (0.50,3.75)
0.26 (0.06,1.22)	0.47 (0.16,1.39)	0.22 (0.01,6.12)	0.51 (0.18,1.49)	0.54 (0.15,1.96)	0.61 (0.17,2.14)	0.89 (0.29,2.72)	0.93 (0.23,3.72)	0.93 (0.28,3.09)	IL-2	1.22 (0.31,4.83)	1.27 (0.33,4.93)
**0.21 (0.05,0.89)**	**0.39 (0.16,0.95)**	0.18 (0.01,4.77)	0.42 (0.18,1.02)	0.44 (0.14,1.39)	0.50 (0.17,1.50)	0.74 (0.29,1.87)	0.76 (0.22,2.66)	0.76 (0.27,2.16)	0.82 (0.21,3.27)	ILT-101	1.05 (0.31,3.52)
**0.20 (0.05,0.83)**	**0.37 (0.16,0.87)**	0.17 (0.01,4.51)	**0.40 (0.17,0.94)**	0.42 (0.14,1.29)	0.48 (0.17,1.40)	0.70 (0.29,1.73)	0.73 (0.22,2.48)	0.73 (0.27,2.00)	0.79 (0.20,3.05)	0.96 (0.28,3.21)	Telitacicept

Figure S4 Results of network meta-analysis for AE

**Cenerimod**													
0.80 (0.01,69.44)	**IL-2**												
0.17 (0.01,4.99)	0.21 (0.01,4.94)	**deucravacitinib**											
0.15 (0.01,4.12)	0.19 (0.01,4.05)	0.90 (0.35,2.32)	**Anifrolumab**										
0.14 (0.01,3.82)	0.18 (0.01,3.75)	0.85 (0.34,2.07)	0.94 (0.58,1.53)	**Belimumab**									
0.14 (0.00,4.31)	0.17 (0.01,4.28)	0.82 (0.22,3.13)	0.92 (0.31,2.76)	0.98 (0.34,2.81)	**Iberdomide**								
0.13 (0.00,3.76)	0.16 (0.01,3.73)	0.75 (0.22,2.56)	0.83 (0.32,2.20)	0.89 (0.35,2.22)	0.91 (0.24,3.51)	**Telitacicept**							
0.12 (0.00,4.39)	0.15 (0.01,4.40)	0.73 (0.14,3.86)	0.81 (0.18,3.59)	0.86 (0.20,3.69)	0.88 (0.15,5.13)	0.97 (0.18,5.21)	**Ustekinumab**						
0.09 (0.00,9.83)	0.12 (0.00,10.40)	0.55 (0.02,16.88)	0.61 (0.02,17.27)	0.65 (0.02,18.11)	0.67 (0.02,21.43)	0.73 (0.02,22.66)	0.76 (0.02,27.91)	**Epratuzumab**					
0.11 (0.00,2.97)	0.13 (0.01,2.93)	0.64 (0.23,1.74)	0.71 (0.37,1.38)	0.75 (0.42,1.36)	0.77 (0.24,2.45)	0.85 (0.31,2.37)	0.87 (0.19,4.02)	1.16 (0.04,33.17)	**Tabalumab**				
0.11 (0.00,2.81)	0.13 (0.01,2.76)	0.63 (0.27,1.48)	0.70 (0.47,1.05)	**0.74 (0.57,0.97)**	0.76 (0.27,2.13)	0.84 (0.35,2.02)	0.86 (0.21,3.61)	1.14 (0.04,31.41)	0.99 (0.58,1.67)	**Placebo**			
0.10 (0.00,2.88)	0.13 (0.01,2.85)	0.60 (0.20,1.80)	0.67 (0.30,1.48)	0.71 (0.34,1.48)	0.73 (0.21,2.50)	0.80 (0.26,2.45)	0.82 (0.17,4.03)	1.09 (0.04,32.18)	0.94 (0.40,2.24)	0.96 (0.48,1.90)	**Sifalimumab**		
0.07 (0.00,3.02)	0.09 (0.00,3.07)	0.41 (0.05,3.22)	0.46 (0.07,3.11)	0.49 (0.07,3.22)	0.50 (0.06,4.21)	0.55 (0.07,4.34)	0.56 (0.05,5.95)	0.75 (0.02,33.55)	0.64 (0.09,4.51)	0.65 (0.10,4.25)	0.68 (0.09,5.02)	**ILT-101**	
0.07 (0.00,1.85)	0.09 (0.00,1.82)	0.40 (0.15,1.05)	**0.45 (0.25,0.81)**	**0.48 (0.29,0.79)**	0.49 (0.16,1.48)	0.54 (0.20,1.43)	0.55 (0.12,2.45)	0.73 (0.03,20.63)	0.63 (0.32,1.24)	0.64 (0.42,0.98)	0.67 (0.30,1.50)	0.98 (0.14,6.68)	**Baricitinib**

Figure S5 Results of network meta-analysis for SAE

**IL-2**										
0.34 (0.06,1.91)	**Emab**									
0.36 (0.05,2.41)	1.07 (0.45,2.55)	**Ustekinumab**								
0.31 (0.06,1.75)	0.92 (0.65,1.31)	0.86 (0.37,2.02)	**Belimumab**							
0.30 (0.05,1.79)	0.89 (0.50,1.58)	0.83 (0.32,2.19)	0.97 (0.56,1.68)	**Tabalumab**						
0.30 (0.05,1.75)	0.88 (0.52,1.49)	0.82 (0.32,2.11)	0.96 (0.58,1.58)	0.99 (0.51,1.94)	**Blisibimod**					
0.30 (0.05,1.64)	0.88 (0.67,1.15)	0.82 (0.36,1.87)	0.95 (0.76,1.19)	0.98 (0.59,1.62)	0.99 (0.63,1.55)	**Placebo**				
0.27 (0.02,3.07)	0.80 (0.14,4.59)	0.75 (0.11,5.06)	0.87 (0.15,4.94)	0.90 (0.15,5.41)	0.91 (0.15,5.38)	0.92 (0.16,5.13)	**E6742**			
0.22 (0.01,3.68)	0.66 (0.07,6.18)	0.62 (0.06,6.58)	0.72 (0.08,6.66)	0.74 (0.08,7.20)	0.75 (0.08,7.19)	0.76 (0.08,6.94)	0.82 (0.05,13.65)	**deucravacitinib**		
0.28 (0.05,1.55)	0.82 (0.57,1.18)	0.76 (0.32,1.80)	0.89 (0.64,1.23)	0.92 (0.52,1.60)	0.93 (0.56,1.54)	0.93 (0.73,1.19)	1.02 (0.18,5.79)	1.23 (0.13,11.48)	**Baricitinib**	
0.20 (0.03,1.26)	0.60 (0.29,1.21)	0.56 (0.19,1.60)	0.65 (0.32,1.29)	0.67 (0.29,1.53)	0.68 (0.31,1.49)	0.68 (0.35,1.31)	0.74 (0.12,4.70)	0.90 (0.09,9.11)	0.73 (0.36,1.47)	**Telitacicept**

Figure S6 Results of network meta-analysis for infection related AEs

Notes: #: Composite responder index based on improvement in disease activity (at least 4 point improvement in SELENA-SLEDAI) without worsening of the overall condition (no worsening in PGA) or the development of significant disease activity in new organ systems (no new BILAG A or >1 new BILAG B). ##: Composite responder index based on improvement in disease activity (at least 4 point improvement in SLEDAI-2K score) without worsening of the overall condition (no worsening in PGA) or the development of significant disease

activity in new organ systems (no new BILAG A or >1 new BILAG B). Estimates are presented as relative risk (RR) and 95% confidence intervals (CI; in parentheses). Comparisons should be read from left to right and the estimate is in the cell in common between the column-defining treatment and the row-defining treatment. If the 95% CI of the RR did not contain 1, the differences were considered statistically significant. Significant results are presented in bold.

Appendix 5 Treatment Relative Ranking

Table S6: SUCRA ranking table of SRI-4 response # as the outcome indicator.

Treatment	SUCRA (%)	PrBest	MeanRank
Telitacicept	95.4	73.2	1.3
IL-2	82.0	26.2	2.1
Belimumab	56.6	0	3.6
ILT-101	46.0	0.6	4.2
Tabalumab	37.8	0	4.7
Blisibimod	26.6	0	5.4
Placebo	5.6	0	6.7

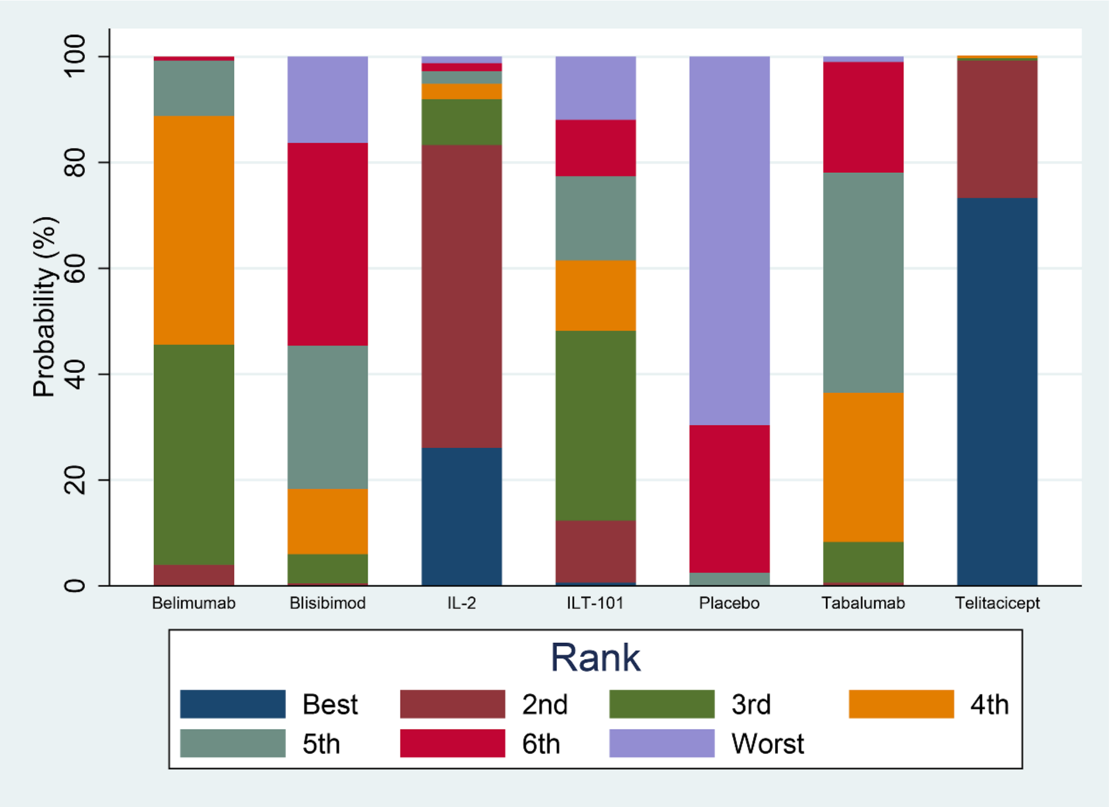

Figure S7 SUCRA ranking of SRI-4 response # presented in bar chart

Table S7: SUCRA ranking table of SRI-4 response ## as the outcome indicator.

Treatment	SUCRA (%)	PrBest	MeanRank
Ustekinumab	90.6	59.7	2.2
ABBV-599 high-dose	77.1	17.2	4
Deucravacitinib	69.3	6	5

Anifrolumab	59	0.4	6.3
Upadacitinib	57.9	3.9	6.5
Sifalimumab	57.5	2	6.5
Iberdomide	56.7	2.4	6.6
Atacicept	52.7	1.5	7.1
PF04236921	47.1	4.2	7.9
Dapirolizumab pegol	45.9	2.7	8
Evobrutinib	33.7	0.1	9.6
Baricitinib	28.8	0	10.3
Epratuzumab	15.9	0	11.9
Placebo	7.6	0	13

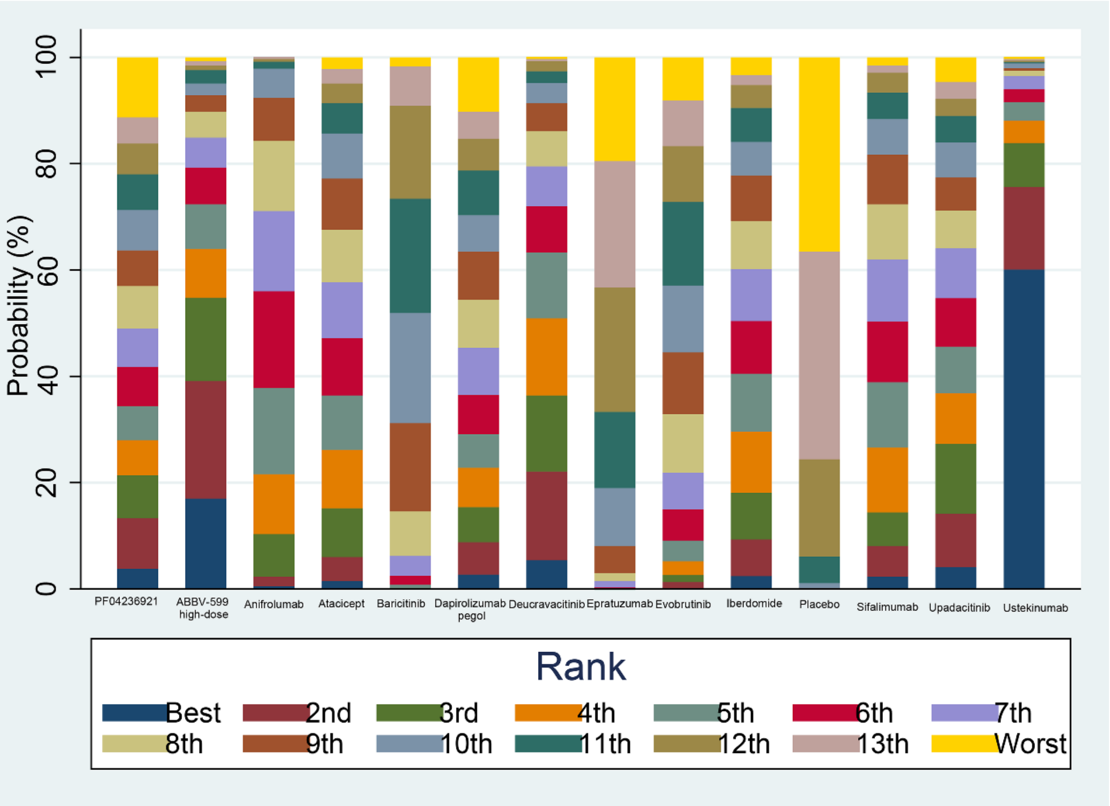

Figure S8 SUCRA ranking of SRI-4 response ## presented in bar chart

Table S8: SUCRA ranking table of BICLA response rate as the outcome indicator.

Treatment	SUCRA (%)	PrBest	MeanRank
Upadacitinib	88.0	41.3	2.2
ABBV-599 high-dose	78.5	15	3.1

PF04236921	71.7	16.6	3.8
Anifrolumab	66.3	1.4	4.4
Deucravacitinib	57.5	1.9	5.3
Dapirolizumab pegol	54.8	3.4	5.5
E6742	51.8	20.1	5.8
Baricitinib	24.5	0	8.5
Epratuzumab	23.8	0	8.6
Ustekinumab	22.7	0.3	8.7
Placebo	10.3	0	10

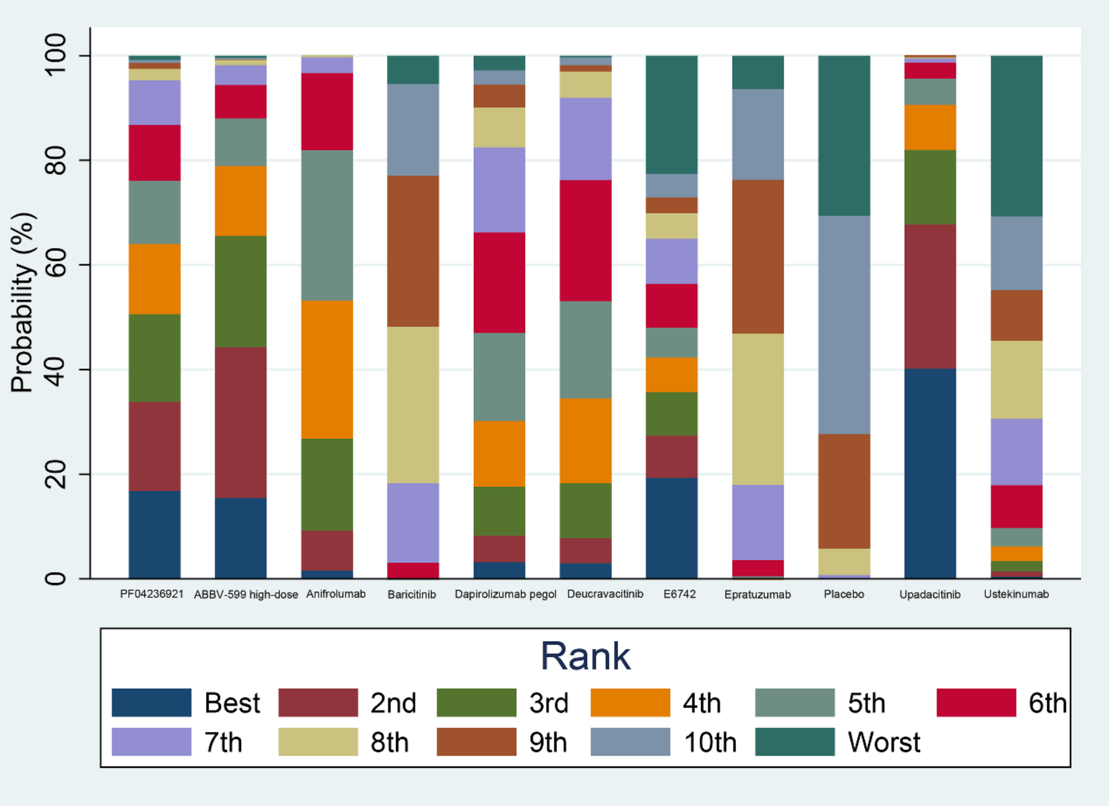

Figure S9 SUCRA ranking of BICLA response presented in bar chart

Table S9: SUCRA ranking table of CLASI-50 response rate as the outcome indicator.

Treatment	SUCRA (%)	PrBest	MeanRank
Deucravacitinib	95.1	77.1	1.3
Anifrolumab	66.1	1.5	3.4
Upadacitinib	64.8	15.5	3.5
Sifalimumab	58.9	1.7	3.9

ABBV-599 high-dose	45.7	3.2	4.8
Iberdomide	38.7	0.9	5.3
Placebo	17.4	0	6.8
Baricitinib	13.3	0	7.1

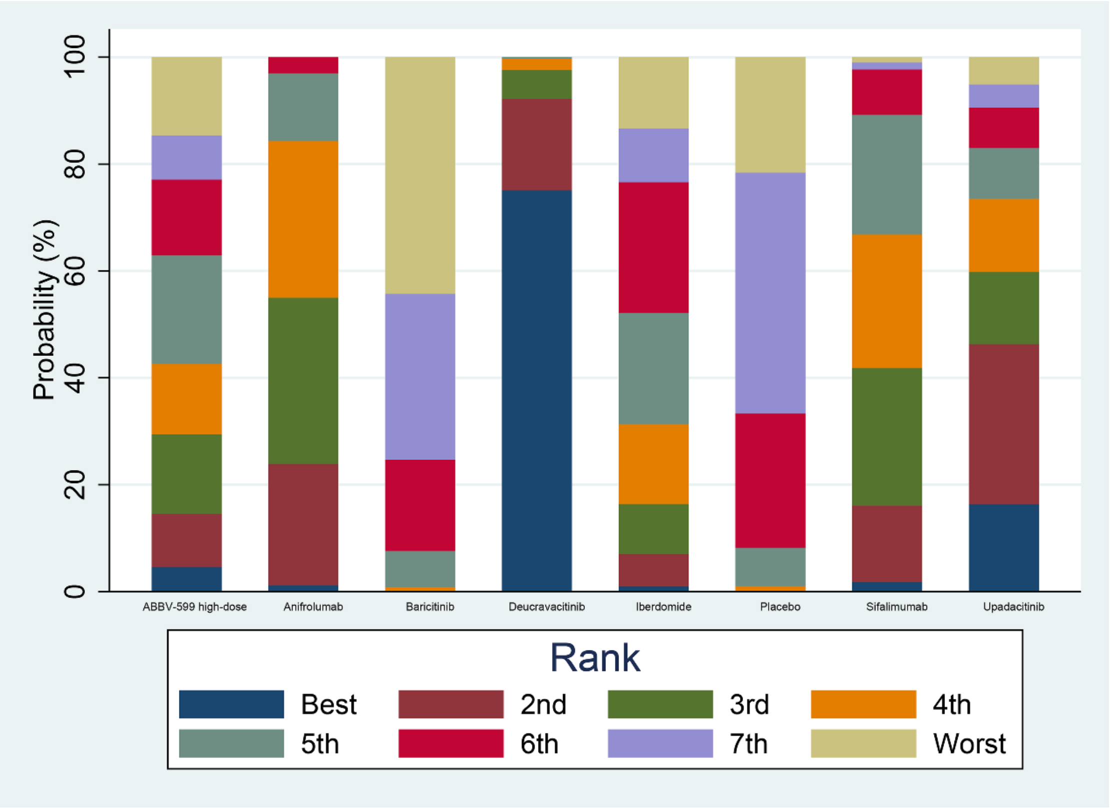

Figure S10 SUCRA ranking of CLASI-50 response presented in bar chart

Table S10: SUCRA ranking table of LLDAS response rate as the outcome indicator.

Treatment	SUCRA (%)	PrBest	MeanRank
Upadacitinib	88.0	59.9	1.5
Deucravacitinib	75.3	33.3	2
ABBV-599 high-dose	59.2	6.8	2.6
Baricitinib	24.4	0	4
Placebo	3.1	0	4.9

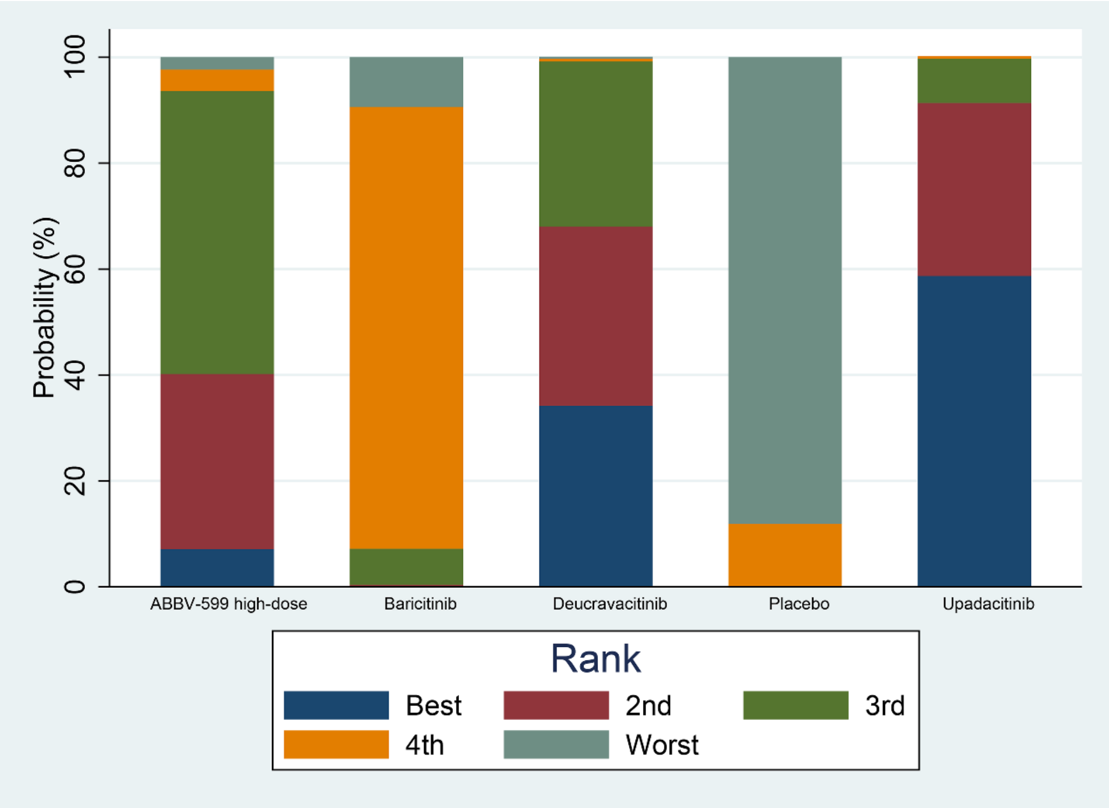

Figure S11 SUCRA ranking of LLDAS response presented in bar chart

Table S11: SUCRA ranking table of AE response rate as the outcome indicator.

Treatment	SUCRA (%)	PrBest	MeanRank
Cenerimod	89.4	39.3	2.2
Belimumab	77.7	1.9	3.4
Epratuzumab	74.8	51.1	3.8
Placebo	68.3	0.2	4.5
Deucravacitinib	64.8	4.2	4.9
Sifalimumab	57.1	1.6	5.7
Anifrolumab	33.4	0	8.3
Ustekinumab	33	0.5	8.4
Iberdomide	31.7	0.1	8.5
IL-2	31.2	0.9	8.6
ILT-101	20.4	0.2	9.8
Telitacicept	18.1	0.1	10

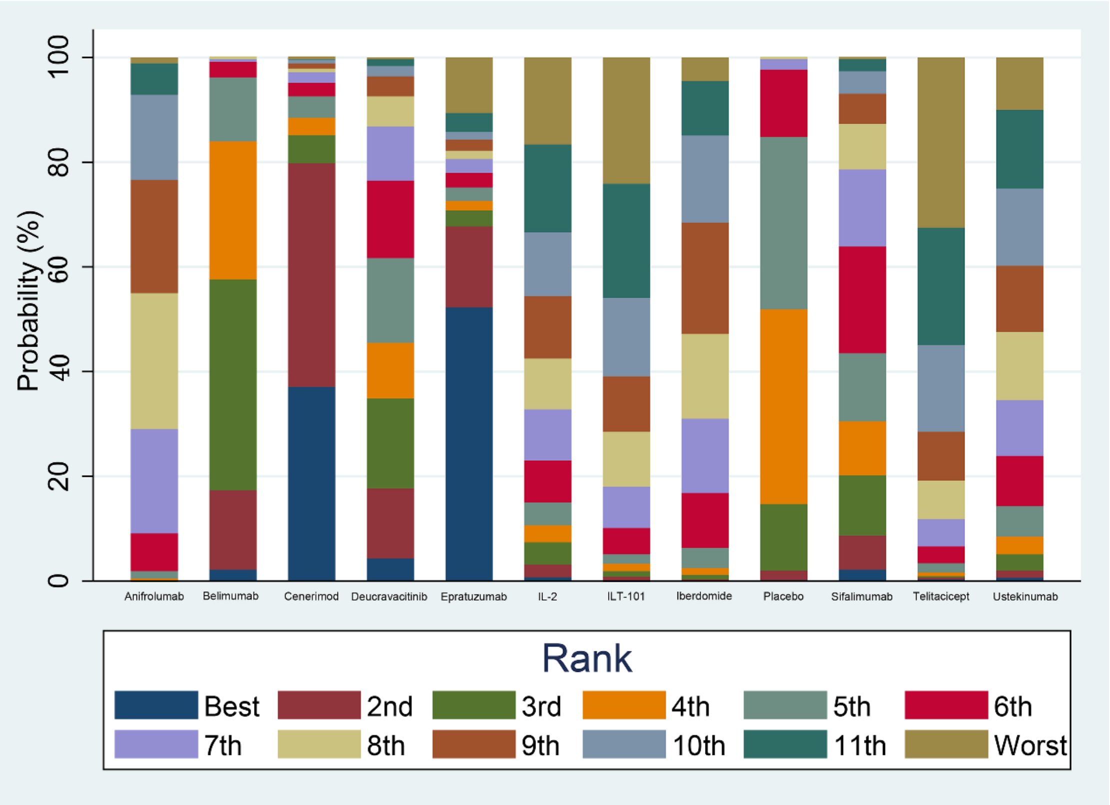

Figure S12 SUCRA ranking of AE response presented in bar chart

Table S12: SUCRA ranking table of SAE response rate as the outcome indicator.

Treatment	SUCRA	PrBest	MeanRank
Cenerimod	86.6	48.3	2.7
IL-2	84.6	41	3
deucravacitinib	64.4	0.9	5.6
Anifrolumab	62.6	0.1	5.9
Belimumab	59.4	0.1	6.3
Iberdomide	54.1	1.1	7
Telitacicept	48.4	0.4	7.7
Ustekinumab	47.6	1.3	7.8
Epratuzumab	40.7	6	8.7
Tabalumab	39	0	8.9
Placebo	36.1	0	9.3
Sifalimumab	35.6	0	9.4
ILT-101	27.2	0.8	10.5

Baricitinib	13.9	0	12.2

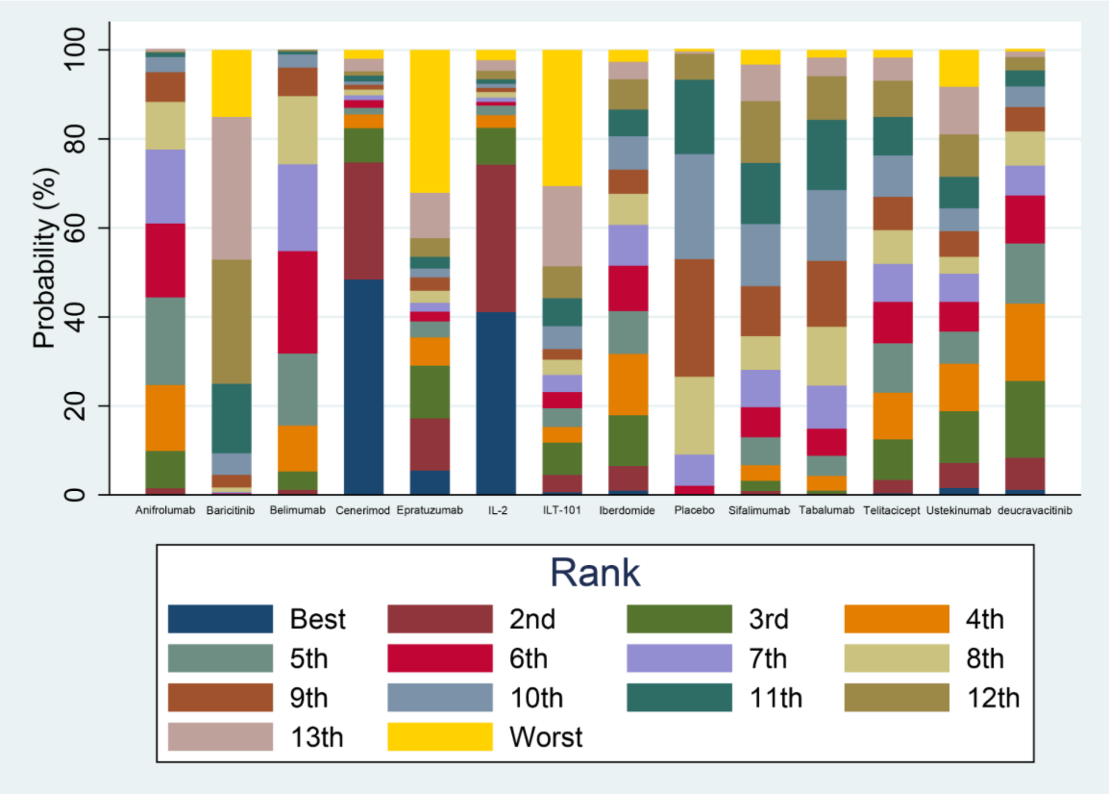

Figure S13 SUCRA ranking of SAE response presented in bar chart

Table S13: SUCRA ranking table of Infection related AEs as the outcome indicator.

Treatment	SUCRA	PrBest	MeanRank
IL-2	89.3	67.8	2.1
Epratuzumab	63.7	1.2	4.6
Ustekinumab	61.6	6.7	4.8
Belimumab	54.3	0.4	5.6
Tabalumab	48.4	1.4	6.2
Blisibimod	48	1	6.2
Placebo	45.6	0	6.4
E6742	45.2	10	6.5
deucravacitinib	38.8	11.1	7.1
Baricitinib	36.3	0	7.4
Telitacicept	18.8	0.2	9.1

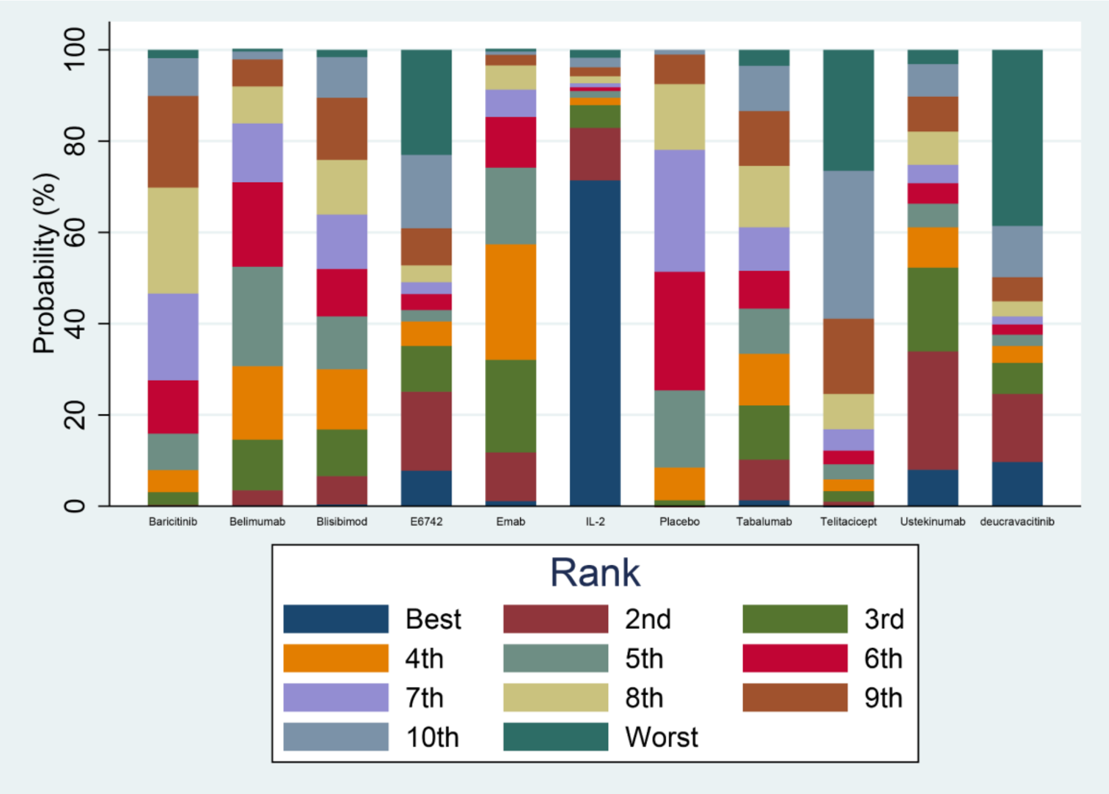

Figure S14 SUCRA ranking of Infection related AEs presented in bar chart

Notes: #: Composite responder index based on improvement in disease activity (at least 4 point improvement in SELENA-SLEDAI) without worsening of the overall condition (no worsening in PGA) or the development of significant disease activity in new organ systems (no new BILAG A or >1 new BILAG B). ##: Composite responder index based on improvement in disease activity (at least 4 point improvement in SLEDAI-2K score) without worsening of the overall condition (no worsening in PGA) or the development of significant disease activity in new organ systems (no new BILAG A or >1 new BILAG B).

Appendix 6 Treatment Relative Ranking in dose subgroup analyses

Table S14: SUCRA Rankings for SRI-4 response # in dose subgroup analyses

Treatment	SUCRA (%)	PrBest	MeanRank
Telitacicept240mgQW	94.5	59	1.6
Telitacicept80mgQW	87.1	18.8	2.4
Telitacicept160mgQW	82.6	8.5	2.9
IL-2	74.6	13.4	3.8
Belimumab10mg/kg	52.9	0	6.2
Belimumab200mg	52.4	0	6.2
ILT-101	40.1	0.3	7.6
Belimumab1mg/kg	31.4	0	8.5
Tabalumab120mgQ4W	30.7	0	8.6
Tabalumab120mgQ2W	29.9	0	8.7
Blisibimod200mgQW	20.4	0	9.8
Placebo	3.4	0	11.6

Table S15: SUCRA Rankings for SRI-4 Response ## in dose subgroup analyses

Treatment	SUCRA (%)	PrBest	MeanRank
Ustekinumab	88.4	36.5	4.2
Deucravacitinib3mg	82.8	11.7	5.8
ABBV-599high-dose	75.8	8.6	7.8
Iberdomide0.45mg	75.8	6.7	7.8
Dapirolizumabpegol6mg/kg	72.2	11.9	8.8
PF0423692110mg	70.3	12	9.3
Sifalimumab1200mg	62.5	1	11.5
Anifrolumab300mg	62	0.1	11.7
Atacicept75mg	60.2	1.2	12.1
Upadacitinib30mg	59.1	1.9	12.5
Sifalimumab200mg	57.9	0.7	12.8

Deucravacitinib6mg	57.8	0.7	12.8
Deucravacitinib12mg	57.4	0.7	12.9
Iberdomide0.15mg	56.3	2.5	13.2
Dapirolizumabpegol45mg/kg	53.4	2.2	14
Sifalimumab600mg	51.9	0.4	14.5
Evobrutinib25mg	48.9	0.3	15.3
Atacicept150mg	47.3	0.4	15.7
Anifrolumab1000mg	45.8	0.1	16.2
Baricitinib4mg	42.8	0	17
Evobrutinib75mg	36.4	0.1	18.8
Iberdomide0.30mg	35.1	0	19.2
PF0423692150mg	26.1	0.2	21.7
Evobrutinib50mg	25.8	0	21.8
Epratuzumab1200mg	23.6	0	22.4
Baricitinib2mg	22.3	0	22.7
Dapirolizumabpegol24mg/kg	20.2	0	23.3
Epratuzumab600mg	18.3	0	23.9
Placebo	13.4	0	25.2

Table S16: SUCRA Rankings for BICLA response in dose subgroup analyses

Treatment	SUCRA (%)	PrBest	MeanRank
Upadacitinib30mg	85.9	21.9	3.7
PF0423692110mg	79.3	19	4.9
ABBV-599high-dose	77.9	6.5	5.2
Deucravacitinib3mg	77.2	6.2	5.3
Anifrolumab300mg	70.1	0.5	6.7
E6742200mg	65.8	29.3	7.5
Dapirolizumabpegol24mg/kg	62.1	3.7	8.2
PF0423692150mg	60.2	3.8	8.6

Dapirolizumabpegol45mg/kg	56.3	2.1	9.3
Anifrolumab1000mg	49.2	0	10.6
Deucravacitinib12mg	48.9	0.1	10.7
Dapirolizumabpegol6mg/kg	47.6	0.8	11
Deucravacitinib6mg	47.6	0.1	11
E6742100mg	36.7	5.9	13
Baricitinib4mg	30.5	0	14.2
Epratuzumab1200mg	25.4	0	15.2
Ustekinumab	24.6	0.2	15.3
Epratuzumab600mg	22.9	0	15.6
Baricitinib2mg	19.7	0	16.3
Placebo	12	0	17.7

Table S17: SUCRA Rankings for CLASI-50 response in dose subgroup analyses

Treatment	SUCRA (%)	PrBest	MeanRank
Deucravacitinib3mg	93.1	50.7	2.1
Deucravacitinib12mg	86.7	16.9	3.1
Deucravacitinib6mg	79.7	6.3	4.3
Anifrolumab150mg	60	9.2	7.4
Upadacitinib30mg	58.6	7.1	7.6
Sifalimumab1200mg	58.4	1.3	7.7
Anifrolumab300mg	56.9	0	7.9
Sifalimumab200mg	56.1	0.8	8
Anifrolumab1000mg	55.2	0.8	8.2
Iberdomide0.15mg	54.4	4.6	8.3
Iberdomide0.45mg	47.8	1.2	9.3
ABBV-599high-dose	41.3	1.2	10.4
Sifalimumab600mg	39.9	0.1	10.6
Placebo	17.5	0	14.2

Baricitinib4mg	16.3	0	14.4
Baricitinib2mg	14.2	0	14.7
Iberdomide0.30mg	14	0	14.8

Table S18: SUCRA Rankings for LLDAS response in dose subgroup analyses

Treatment	SUCRA (%)	PrBest	MeanRank
Deucravacitinib3mg	91.2	57.5	1.6
Upadacitinib30mg	82.3	34.5	2.2
Deucravacitinib12mg	63	3.4	3.6
ABBV-599high-dose	58.4	3.4	3.9
Deucravacitinib6mg	55.1	1.1	4.1
Baricitinib4mg	30.5	0	5.9
Baricitinib2mg	13.5	0	7.1
Placebo	6	0	7.6

Table S19: SUCRA Rankings for AE response rate in dose subgroup analyses

Treatment	SUCRA (%)	PrBest	MeanRank
Epratuzumab100mg	89.2	47.9	4.2
Cenerimod4mg	81.1	8.5	6.7
Cenerimod0.5mg	78	6.1	7.6
Cenerimod1mg	77.2	5.5	7.8
Epratuzumab1200mg	76.7	14.5	8
Cenerimod2mg	73	3.3	9.1
Belimumab200mg	72.6	0.2	9.2
Deucravacitinib12mg	70.8	1.1	9.8
Belimumab4mg/kg	69.5	2.2	10.1
Belimumab10mg/kg	65	0	11.5
Deucravacitinib6mg	61.3	0.4	12.6
Placebo	60.2	0	12.9

Sifalimumab1200mg	59.7	0.2	13.1
Belimumab1mg/kg	58.5	0	13.4
Epratuzumab400mg	51	5.4	15.7
Epratuzumab600mg	49.9	4.7	16
Sifalimumab600mg	45.6	0.1	17.3
Sifalimumab200mg	45.5	0	17.3
Anifrolumab150mg	39.9	0	19
Iberdomide0.15mg	39.4	0	19.2
Ustekinumab	31.1	0	21.7
Anifrolumab300mg	29.4	0	22.2
Anifrolumab1000mg	29.1	0	22.3
IL-2	29	0	22.3
Telitacicept80mg	28.5	0	22.4
Iberdomide0.45mg	28.4	0	22.5
Deucravacitinib3mg	27.8	0	22.7
Iberdomide0.30mg	27.1	0	22.9
ILT-101	20.6	0	24.8
Telitacicept160mg	20.6	0	24.8
Telitacicept240mg	14.4	0	26.7

Table S20: SUCRA Rankings for SAE response rate in dose subgroup analyses

Treatment	SUCRA	PrBest	MeanRank
IL-2	85.5	35	6.2
Belimumab10mg/kgIV	74.6	0.1	10.1
Iberdomide0.30mg	66.8	1.2	12.9
deucravacitinib3mg	66	0.4	13.2
Belimumab200mgSC	65.9	0	13.3
deucravacitinib12mg	64.6	0.3	13.7
Cenerimod2mg	64.5	11.4	13.8

Belimumab4.0mg/kg	64	0.1	14
Cenerimod4mg	63.8	11.5	14
Anifrolumab300mg	63.2	0	14.3
Cenerimod0.5mg	62.5	10.9	14.5
Cenerimod1mg	62.3	9.4	14.6
Anifrolumab150mg	60.8	0.1	15.1
deucravacitinib6mg	60.2	0.1	15.3
Telitacicept80mg	54.8	0.2	17.3
Telitacicept240mg	54.7	0.2	17.3
Belimumab10mg/kg	52.4	0	18.1
Sifalimumab200mg	52.4	0	18.2
Anifrolumab1,000mg	51.9	0	18.3
Iberdomide0.15mg	50.4	0.4	18.8
Tabalumab120Q2W	50.3	0	18.9
Ustekinumab	49.2	0.6	19.3
Iberdomide0.45mg	48.7	0.1	19.5
Epratuzumab400mg	48.3	8.1	19.6
Epratuzumab100mg	47.5	8.4	19.9
Belimumab1.0mg/kg	43.5	0	21.3
Telitacicept160mg	43.3	0	21.4
Placebo	38.9	0	23
Sifalimumab1200mg	33.6	0	24.9
Tabalumab120Q4W	32.3	0	25.4
Belimumab1mg/kg	31.9	0	25.5
Sifalimumab600mg	31.3	0	25.7
ILT-101	30.7	0.3	25.9
Epratuzumab1200mg	21.5	0.6	29.3
Epratuzumab600mg	21.1	0.4	29.4
Baricitinib4mg	19.6	0	29.9

Baricitinib2mg	16.8	0	30.9

Table S21: SUCRA Rankings for Infection related AEs in dose subgroup analyses

Treatment	SUCRA	PrBest	MeanRank
IL-2	89.1	50.2	3.5
Belimumab200mg	76.8	3.1	6.3
Emab1200mg	72.1	0.2	7.4
Ustekinumab	64.1	2.9	9.3
Belimumab10mg/kg	60.9	0	10
Emab600mg	58.8	0	10.5
Tabalumab120mgQ2W	58	0.8	10.7
Blisibimod200mgQW	52.7	0.2	11.9
Placebo	52.2	0	12
deucravacitinib3mg	51.3	12.8	12.2
Belimumab10.0mg/kg	51	0.4	12.3
E6742200mg	50.9	8.3	12.3
deucravacitinib12mg	50.7	12.8	12.3
Tabalumab120mgQ4W	49.6	0.2	12.6
Baricitinib2mg	45.7	0	13.5
Belimumab1.0mg/kg	44.6	0.2	13.8
Baricitinib4mg	44.5	0	13.8
E6742100mg	44.3	5.4	13.8
Belimumab1mg/kg	42.4	0	14.2
Telitacicept80mg	37.9	0.4	15.3
deucravacitinib6mg	30.2	1.8	17.1
Telitacicept160mg	27.1	0.2	17.8
Belimumab4.0mg/kg	26	0	18
Telitacicept240mg	19.3	0.1	19.6

Notes: #: Composite responder index based on improvement in disease activity (at least 4 point improvement in SELENA-SLEDAI) without worsening of the overall condition (no worsening in PGA) or the development of significant disease activity in new organ systems (no new BILAG A or >1 new BILAG B). ##: Composite responder index based on improvement in disease activity (at least 4 point improvement in SLEDAI-2K score) without worsening of the overall condition (no worsening in PGA) or the development of significant disease activity in new organ systems (no new BILAG A or >1 new BILAG B).

Appendix 7 Results of network meta-analyses in dose subgroups

**Telitacicept240mgQW**											
1.28 (0.58,2.85)	**Telitacicept80mgQW**										
1.46 (0.66,3.20)	1.14 (0.53,2.44)	**Telitacicept 160mgQW**									
1.86 (0.50,7.00)	1.45 (0.39,5.39)	1.28 (0.35,4.70)	**IL-2**								
**3.66 (1.63,8.20)**	**2.85 (1.30,6.25)**	**2.51 (1.16,5.43)**	1.96 (0.66,5.80)	**Belimumab10mg/kg**							
**3.61 (1.56,8.33)**	**2.82 (1.25,6.35)**	**2.48 (1.11,5.52)**	1.94 (0.64,5.85)	0.99 (0.70,1.40)	**Belimumab200mg**						
**3.98 (1.28,12.34)**	**3.10 (1.02,9.47)**	2.73 (0.90,8.26)	2.13 (0.56,8.18)	1.09 (0.47,2.52)	1.10 (0.46,2.62)	**ILT-101**					
**4.43 (1.96,10.03)**	**3.46 (1.56,7.64)**	**3.04 (1.39,6.64)**	2.38 (0.80,7.08)	1.21 (0.97,1.52)	1.23 (0.85,1.78)	1.11 (0.48,2.60)	**Belimumab1mg/kg**				
**4.50 (1.95,10.38)**	**3.51 (1.56,7.92)**	**3.09 (1.39,6.88)**	2.42 (0.80,7.29)	1.23 (0.87,1.74)	1.25 (0.83,1.88)	1.13 (0.48,2.70)	1.02 (0.70,1.47)	**Tabalumab120mgQ4W**			
**4.52 (1.96,10.43)**	**3.53 (1.57,7.95)**	**3.10 (1.39,6.91)**	2.43 (0.80,7.33)	1.24 (0.87,1.75)	1.25 (0.83,1.89)	1.14 (0.48,2.71)	1.02 (0.71,1.47)	1.00 (0.76,1.34)	**Tabalumab120mgQ2W**		
**5.06 (2.12,12.08)**	**3.95 (1.69,9.22)**	**3.47 (1.50,8.02)**	2.72 (0.88,8.42)	1.38 (0.91,2.11)	1.40 (0.87,2.26)	1.27 (0.52,3.13)	1.14 (0.74,1.78)	1.12 (0.70,1.81)	1.12 (0.70,1.80)	**Blisibimod200mgQW**	
**6.12 (2.79,13.40)**	**4.77 (2.23,10.20)**	**4.20 (1.99,8.86)**	**3.28 (1.13,9.54)**	**1.67 (1.38,2.03)**	**1.69 (1.27,2.27)**	1.54 (0.68,3.49)	**1.38 (1.10,1.73)**	**1.36 (1.02,1.81)**	**1.35 (1.01,1.80)**	1.21 (0.83,1.76)	**Placebo**

Figure S15. Results of network meta - analysis of treatment measures grouped by dosing for SRI-4 response #

**Ustekinumab**																												
1.27 (0.43,3.76)	**Deucravacitinib3mg**																											
1.43 (0.46,4.43)	1.13 (0.42,3.00)	**ABBV-599 high-dose**																										
1.45 (0.48,4.38)	1.15 (0.44,2.95)	1.02 (0.38,2.76)	**Iberdomide 0.45mg**																									
1.46 (0.41,5.18)	1.15 (0.37,3.57)	1.02 (0.32,3.32)	1.01 (0.32,3.16)	**Dapirolizumab pegol 6mg/kg**																								
1.49 (0.41,5.38)	1.17 (0.37,3.72)	1.04 (0.31,3.45)	1.02 (0.32,3.30)	1.02 (0.27,3.83)	**PF04236921 10mg**																							
1.80 (0.62,5.16)	1.42 (0.58,3.45)	1.26 (0.49,3.24)	1.24 (0.50,3.07)	1.23 (0.41,3.70)	1.21 (0.39,3.73)	**Sifalimumab1200mg**																						
1.85 (0.74,4.64)	1.46 (0.71,3.01)	1.30 (0.59,2.86)	1.27 (0.60,2.69)	1.27 (0.48,3.35)	1.25 (0.46,3.38)	1.03 (0.52,2.03)	**Anifrolumab300mg**																					
1.84 (0.64,5.34)	1.45 (0.59,3.58)	1.29 (0.50,3.35)	1.27 (0.51,3.18)	1.26 (0.42,3.83)	1.24 (0.40,3.85)	1.03 (0.43,2.43)	1.00 (0.50,1.98)	**Atacicept75mg**																				
1.84 (0.59,5.77)	1.45 (0.54,3.92)	1.29 (0.62,2.70)	1.27 (0.46,3.47)	1.26 (0.38,4.12)	1.24 (0.37,4.15)	1.02 (0.39,2.67)	0.99 (0.44,2.23)	1.00 (0.38,2.63)	**Upadacitinib30mg**																			
1.91 (0.66,5.48)	1.51 (0.62,3.66)	1.34 (0.52,3.44)	1.31 (0.53,3.26)	1.31 (0.43,3.93)	1.29 (0.42,3.96)	1.06 (0.58,1.95)	1.03 (0.53,2.02)	1.04 (0.44,2.45)	1.04 (0.40,2.70)	**Sifalimumab 200mg**																		
1.88 (0.64,5.57)	1.48 (0.78,2.81)	1.32 (0.50,3.51)	1.30 (0.50,3.33)	1.29 (0.42,3.99)	1.27 (0.40,4.02)	1.05 (0.43,2.55)	1.02 (0.50,2.09)	1.02 (0.42,2.51)	1.02 (0.38,2.76)	0.99 (0.41,2.39)	**Deucravacitinib 6mg**																	
1.89 (0.64,5.62)	1.49 (0.78,2.84)	1.33 (0.50,3.54)	1.30 (0.51,3.36)	1.30 (0.42,4.02)	1.27 (0.40,4.05)	1.05 (0.43,2.57)	1.02 (0.50,2.11)	1.03 (0.42,2.53)	1.03 (0.38,2.78)	0.99 (0.41,2.41)	1.00 (0.53,1.90)	**Deucravacitinib 12mg**																
1.90 (0.58,6.19)	1.50 (0.53,4.22)	1.33 (0.45,3.93)	1.31 (0.59,2.89)	1.30 (0.38,4.41)	1.28 (0.37,4.44)	1.06 (0.39,2.88)	1.03 (0.44,2.42)	1.03 (0.38,2.83)	1.03 (0.35,3.09)	1.00 (0.37,2.71)	1.01 (0.36,2.83)	1.00 (0.36,2.83)	**Iberdomide0.15mg**															
1.98 (0.57,6.83)	1.56 (0.52,4.70)	1.38 (0.44,4.36)	1.36 (0.44,4.16)	1.35 (0.54,3.40)	1.33 (0.36,4.88)	1.10 (0.38,3.21)	1.07 (0.42,2.73)	1.07 (0.36,3.16)	1.07 (0.34,3.42)	1.04 (0.36,3.02)	1.05 (0.35,3.15)	1.04 (0.35,3.15)	1.04 (0.31,3.43)	**Dapirolizumab pegol 45mg/kg**														
2.06 (0.72,5.91)	1.62 (0.67,3.95)	1.44 (0.56,3.70)	1.42 (0.57,3.51)	1.41 (0.47,4.23)	1.39 (0.45,4.27)	1.15 (0.63,2.10)	1.11 (0.57,2.18)	1.12 (0.47,2.64)	1.12 (0.43,2.91)	1.08 (0.59,1.97)	1.09 (0.45,2.65)	1.09 (0.45,2.65)	1.08 (0.40,2.94)	1.04 (0.36,3.04)	**Sifalimumab 600mg**													
2.15 (0.76,6.12)	1.70 (0.70,4.08)	1.51 (0.59,3.83)	1.48 (0.60,3.63)	1.47 (0.49,4.39)	1.45 (0.47,4.42)	1.20 (0.52,2.77)	1.16 (0.60,2.25)	1.17 (0.50,2.73)	1.17 (0.45,3.01)	1.13 (0.49,2.60)	1.14 (0.48,2.74)	1.14 (0.47,2.74)	1.13 (0.42,3.05)	1.09 (0.38,3.15)	1.04 (0.45,2.41)	**Evobrutinib25mg**												
2.17 (0.75,6.26)	1.71 (0.70,4.19)	1.52 (0.59,3.93)	1.49 (0.60,3.73)	1.48 (0.49,4.49)	1.46 (0.47,4.52)	1.21 (0.51,2.85)	1.17 (0.59,2.32)	1.18 (0.64,2.17)	1.18 (0.45,3.09)	1.14 (0.48,2.67)	1.15 (0.47,2.81)	1.15 (0.47,2.81)	1.14 (0.42,3.12)	1.10 (0.37,3.22)	1.05 (0.45,2.48)	1.01 (0.43,2.35)	**Atacicept 150mg**											
2.21 (0.79,6.18)	1.74 (0.74,4.11)	1.55 (0.62,3.86)	1.52 (0.63,3.65)	1.51 (0.52,4.43)	1.49 (0.50,4.47)	1.23 (0.54,2.78)	1.19 (0.69,2.08)	1.20 (0.52,2.74)	1.20 (0.48,3.04)	1.16 (0.51,2.61)	1.17 (0.50,2.76)	1.17 (0.50,2.75)	1.16 (0.44,3.07)	1.12 (0.39,3.18)	1.07 (0.48,2.42)	1.03 (0.46,2.29)	1.02 (0.45,2.32)	**Anifrolumab1000mg**										
2.31 (0.93,5.75)	1.82 (0.89,3.72)	1.62 (0.74,3.53)	1.59 (0.76,3.32)	1.58 (0.60,4.15)	1.56 (0.58,4.19)	1.29 (0.66,2.50)	1.25 (0.83,1.89)	1.25 (0.64,2.47)	1.26 (0.57,2.79)	1.21 (0.62,2.35)	1.23 (0.60,2.49)	1.22 (0.60,2.50)	1.22 (0.52,2.84)	1.17 (0.46,2.96)	1.12 (0.58,2.17)	1.07 (0.56,2.05)	1.07 (0.54,2.09)	1.05 (0.56,1.94)	**Baricitinib4mg**									
2.52 (0.89,7.16)	1.99 (0.83,4.78)	1.76 (0.69,4.48)	1.73 (0.71,4.25)	1.72 (0.58,5.13)	1.70 (0.56,5.17)	1.40 (0.61,3.24)	1.36 (0.71,2.63)	1.37 (0.59,3.19)	1.37 (0.53,3.53)	1.32 (0.57,3.04)	1.34 (0.56,3.20)	1.33 (0.55,3.20)	1.33 (0.49,3.56)	1.27 (0.44,3.68)	1.22 (0.53,2.82)	1.17 (0.66,2.09)	1.16 (0.50,2.70)	1.14 (0.51,2.54)	1.09 (0.57,2.08)	**Evobrutinib75mg**								
2.57 (0.85,7.74)	2.02 (0.78,5.22)	1.80 (0.66,4.88)	1.77 (0.90,3.47)	1.76 (0.56,5.54)	1.73 (0.54,5.58)	1.43 (0.57,3.56)	1.39 (0.66,2.94)	1.39 (0.55,3.50)	1.40 (0.51,3.84)	1.34 (0.54,3.34)	1.36 (0.53,3.51)	1.36 (0.53,3.50)	1.35 (0.61,2.99)	1.30 (0.42,3.98)	1.25 (0.50,3.09)	1.19 (0.49,2.93)	1.18 (0.47,2.96)	1.16 (0.48,2.80)	1.11 (0.53,2.33)	1.02 (0.41,2.50)	**Iberdomide 0.30mg**							
3.11 (0.86,11.28)	2.45 (0.77,7.80)	2.18 (0.66,7.23)	2.14 (0.66,6.91)	2.13 (0.57,8.02)	2.10 (0.78,5.60)	1.73 (0.56,5.35)	1.68 (0.62,4.58)	1.69 (0.54,5.25)	1.69 (0.51,5.67)	1.63 (0.53,5.02)	1.65 (0.52,5.24)	1.64 (0.52,5.23)	1.64 (0.47,5.68)	1.57 (0.43,5.78)	1.51 (0.49,4.65)	1.45 (0.47,4.42)	1.44 (0.46,4.45)	1.41 (0.47,4.23)	1.35 (0.50,3.63)	1.24 (0.40,3.77)	1.21 (0.38,3.92)	**PF04236921 50mg**						
**2.89 (1.02,8.23)**	2.28 (0.95,5.50)	2.03 (0.80,5.16)	1.99 (0.81,4.89)	1.98 (0.67,5.90)	1.95 (0.64,5.95)	1.61 (0.70,3.73)	1.57 (0.81,3.03)	1.57 (0.67,3.67)	1.57 (0.61,4.06)	1.52 (0.66,3.50)	1.54 (0.64,3.69)	1.53 (0.64,3.69)	1.52 (0.57,4.10)	1.47 (0.51,4.23)	1.41 (0.61,3.24)	1.35 (0.75,2.41)	1.34 (0.57,3.11)	1.31 (0.59,2.92)	1.25 (0.66,2.39)	1.15 (0.64,2.05)	1.13 (0.46,2.77)	0.93 (0.30,2.84)	**Evobrutinib50mg**					
**2.89 (1.15,7.28)**	**2.28 (1.10,4.73)**	2.02 (0.91,4.48)	1.99 (0.94,4.22)	1.98 (0.75,5.25)	1.95 (0.71,5.31)	1.61 (0.81,3.18)	**1.56 (1.00,2.43)**	1.57 (0.78,3.14)	1.57 (0.70,3.53)	1.51 (0.77,2.98)	1.53 (0.74,3.17)	1.53 (0.74,3.17)	1.52 (0.64,3.60)	1.46 (0.57,3.75)	1.40 (0.71,2.76)	1.34 (0.69,2.61)	1.33 (0.67,2.66)	1.31 (0.69,2.47)	1.25 (0.82,1.91)	1.15 (0.59,2.23)	1.13 (0.53,2.39)	0.93 (0.34,2.53)	1.00 (0.51,1.94)	**Epratuzumab 1200mg**				
**2.91 (1.17,7.24)**	**2.30 (1.13,4.68)**	2.04 (0.94,4.45)	2.01 (0.96,4.18)	2.00 (0.76,5.22)	1.96 (0.73,5.28)	1.62 (0.84,3.15)	**1.58 (1.04,2.38)**	1.58 (0.81,3.11)	1.59 (0.72,3.51)	1.53 (0.79,2.95)	1.55 (0.76,3.14)	1.54 (0.76,3.14)	1.53 (0.66,3.57)	1.47 (0.58,3.73)	1.42 (0.73,2.73)	1.36 (0.71,2.58)	1.34 (0.69,2.63)	1.32 (0.71,2.44)	1.26 (0.95,1.67)	1.16 (0.61,2.20)	1.14 (0.54,2.37)	0.94 (0.35,2.52)	1.01 (0.53,1.92)	1.01 (0.66,1.54)	**Baricitinib 2mg**			
3.37 (0.98,11.61)	2.66 (0.89,7.98)	2.36 (0.75,7.41)	2.32 (0.76,7.07)	2.31 (0.92,5.78)	2.27 (0.62,8.30)	1.88 (0.65,5.46)	1.82 (0.72,4.63)	1.83 (0.62,5.36)	1.83 (0.58,5.81)	1.77 (0.61,5.13)	1.79 (0.60,5.36)	1.78 (0.59,5.35)	1.77 (0.54,5.83)	1.71 (0.71,4.12)	1.64 (0.56,4.75)	1.57 (0.54,4.51)	1.56 (0.53,4.55)	1.53 (0.54,4.31)	1.46 (0.58,3.68)	1.34 (0.47,3.85)	1.31 (0.43,4.01)	1.08 (0.30,3.96)	1.16 (0.40,3.35)	1.17 (0.46,2.98)	1.16 (0.46,2.91)	**Dapirolizumab pegol 24mg/kg**		
**3.08 (1.22,7.77)**	**2.43 (1.17,5.05)**	2.16 (0.97,4.79)	2.12 (1.00,4.50)	2.11 (0.80,5.60)	2.08 (0.76,5.66)	1.72 (0.87,3.39)	**1.67 (1.07,2.60)**	1.67 (0.84,3.35)	1.68 (0.75,3.77)	1.62 (0.82,3.19)	1.64 (0.79,3.38)	1.63 (0.79,3.38)	1.62 (0.69,3.84)	1.56 (0.61,4.00)	1.50 (0.76,2.95)	1.43 (0.74,2.79)	1.42 (0.71,2.84)	1.40 (0.74,2.64)	1.33 (0.87,2.04)	1.22 (0.63,2.38)	1.20 (0.56,2.56)	0.99 (0.36,2.70)	1.07 (0.55,2.07)	1.07 (0.78,1.47)	1.06 (0.69,1.61)	0.91 (0.36,2.33)	**Epratuzumab 600mg**	
**3.22 (1.35,7.66)**	**2.54 (1.32,4.89)**	**2.25 (1.09,4.67)**	**2.21 (1.12,4.38)**	2.20 (0.88,5.54)	2.17 (0.84,5.61)	1.79 (0.98,3.27)	**1.74 (1.28,2.36)**	1.75 (0.94,3.24)	1.75 (0.83,3.69)	1.69 (0.93,3.07)	1.71 (0.89,3.28)	1.70 (0.88,3.28)	1.69 (0.76,3.77)	1.63 (0.67,3.95)	1.56 (0.86,2.84)	1.50 (0.83,2.68)	1.48 (0.81,2.74)	1.46 (0.84,2.53)	**1.39 (1.05,1.84)**	1.28 (0.71,2.29)	1.25 (0.63,2.49)	1.03 (0.40,2.68)	1.11 (0.62,1.99)	1.11 (0.81,1.53)	1.10 (0.84,1.45)	0.95 (0.40,2.30)	1.04 (0.76,1.43)	**Placebo**

Figure S16. Results of network meta - analysis of treatment measures grouped by dosing for SRI-4 response [##]

**Upadacitinib30mg**																			
1.12 (0.34,3.72)	**PF04236921 10mg**																		
1.21 (0.61,2.40)	1.07 (0.33,3.52)	**ABBV-599 high-dose**																	
1.29 (0.49,3.34)	1.14 (0.36,3.58)	1.06 (0.41,2.74)	**Deucravacitinib 3mg**																
1.49 (0.69,3.22)	1.33 (0.49,3.58)	1.23 (0.58,2.63)	1.16 (0.59,2.30)	**Anifrolumab 300mg**															
1.26 (0.14,10.94)	1.12 (0.12,10.64)	1.04 (0.12,9.02)	0.98 (0.12,8.27)	0.84 (0.11,6.61)	**E6742 200mg**														
1.66 (0.54,5.08)	1.47 (0.41,5.32)	1.37 (0.45,4.16)	1.29 (0.45,3.73)	1.11 (0.45,2.73)	1.32 (0.14,12.04)	**Dapirolizumab pegol 24mg/kg**													
1.67 (0.50,5.51)	1.48 (0.58,3.78)	1.38 (0.42,4.52)	1.30 (0.41,4.06)	1.12 (0.41,3.02)	1.32 (0.14,12.60)	1.01 (0.28,3.63)	**PF0423692 150mg**												
1.82 (0.60,5.54)	1.62 (0.45,5.81)	1.51 (0.50,4.54)	1.42 (0.49,4.07)	1.22 (0.50,2.97)	1.45 (0.16,13.19)	1.10 (0.48,2.52)	1.09 (0.31,3.92)	**Dapirolizumab pegol 45mg/kg**											
2.07 (0.85,5.01)	1.84 (0.62,5.44)	1.71 (0.71,4.10)	1.61 (0.71,3.62)	1.39 (0.84,2.30)	1.64 (0.20,13.48)	1.25 (0.46,3.39)	1.24 (0.42,3.67)	1.13 (0.42,3.06)	**Anifrolumab1000mg**										
2.05 (0.78,5.38)	1.82 (0.58,5.77)	1.70 (0.66,4.41)	1.60 (0.88,2.90)	1.38 (0.69,2.76)	1.63 (0.19,13.84)	1.24 (0.42,3.61)	1.23 (0.39,3.89)	1.13 (0.39,3.26)	0.99 (0.44,2.26)	**Deucravacitinib12mg**									
2.08 (0.68,6.40)	1.85 (0.51,6.70)	1.73 (0.57,5.25)	1.62 (0.56,4.70)	1.40 (0.57,3.44)	1.66 (0.18,15.15)	1.26 (0.54,2.92)	1.25 (0.35,4.52)	1.14 (0.50,2.63)	1.01 (0.37,2.75)	1.02 (0.35,2.97)	**Dapirolizumab pegol 6mg/kg**								
2.09 (0.80,5.48)	1.86 (0.59,5.87)	1.73 (0.67,4.48)	1.63 (0.90,2.94)	1.40 (0.70,2.80)	1.66 (0.20,14.10)	1.26 (0.43,3.68)	1.26 (0.40,3.96)	1.15 (0.40,3.31)	1.01 (0.45,2.30)	1.02 (0.56,1.87)	1.01 (0.34,2.93)	**Deucravacitinib 6mg**							
2.79 (0.34,23.25)	2.49 (0.27,22.65)	2.32 (0.28,19.16)	2.17 (0.27,17.56)	1.88 (0.25,14.00)	2.22 (0.28,17.63)	1.69 (0.19,14.76)	1.68 (0.18,15.28)	1.53 (0.18,13.36)	1.35 (0.17,10.59)	1.36 (0.17,11.05)	1.34 (0.15,11.76)	1.34 (0.16,10.81)	**E6742 100mg**						
**2.78 (1.30,5.96)**	2.47 (0.92,6.63)	**2.30 (1.09,4.86)**	**2.16 (1.10,4.25)**	**1.87 (1.29,2.69)**	2.21 (0.28,17.26)	1.68 (0.69,4.10)	1.67 (0.62,4.48)	1.53 (0.63,3.69)	1.35 (0.76,2.38)	1.36 (0.68,2.70)	1.34 (0.55,3.27)	1.33 (0.67,2.63)	0.99 (0.13,7.41)	**Baricitinib 4mg**					
**2.93 (1.36,6.31)**	2.61 (0.97,7.02)	**2.43 (1.15,5.15)**	**2.28 (1.16,4.50)**	**1.97 (1.35,2.86)**	2.33 (0.30,18.23)	1.77 (0.72,4.33)	1.76 (0.65,4.73)	1.61 (0.66,3.90)	1.42 (0.80,2.53)	1.43 (0.72,2.86)	1.41 (0.57,3.46)	1.40 (0.71,2.78)	1.05 (0.14,7.83)	1.06 (0.74,1.51)	**Epratuzumab 1200mg**				
**3.11 (1.04,9.37)**	2.77 (0.78,9.83)	2.58 (0.87,7.68)	2.42 (0.85,6.87)	2.09 (0.87,5.01)	2.48 (0.27,22.42)	1.88 (0.57,6.21)	1.87 (0.53,6.63)	1.71 (0.52,5.60)	1.51 (0.57,4.01)	1.52 (0.53,4.34)	1.50 (0.45,4.95)	1.49 (0.52,4.24)	1.11 (0.13,9.66)	1.12 (0.47,2.67)	1.06 (0.44,2.54)	**Ustekinumab**			
**3.00 (1.40,6.45)**	2.67 (0.99,7.17)	**2.49 (1.17,5.26)**	**2.33 (1.18,4.60)**	**2.01 (1.39,2.92)**	2.39 (0.31,18.64)	1.81 (0.74,4.43)	1.80 (0.67,4.84)	1.65 (0.68,3.99)	1.45 (0.82,2.58)	1.46 (0.73,2.92)	1.44 (0.59,3.54)	1.43 (0.72,2.85)	1.07 (0.14,8.00)	1.08 (0.76,1.54)	1.02 (0.79,1.32)	0.96 (0.40,2.30)	**Epratuzumab 600mg**		
**3.12 (1.46,6.69)**	**2.78 (1.04,7.45)**	**2.59 (1.23,5.46)**	**2.43 (1.24,4.77)**	**2.09 (1.45,3.02)**	2.48 (0.32,19.38)	1.89 (0.77,4.60)	1.87 (0.70,5.02)	1.71 (0.71,4.14)	1.51 (0.85,2.68)	1.52 (0.77,3.03)	1.50 (0.61,3.67)	1.49 (0.75,2.95)	1.12 (0.15,8.32)	1.12 (0.88,1.44)	1.06 (0.74,1.52)	1.00 (0.42,2.39)	1.04 (0.73,1.49)	**Baricitinib 2mg**	
**3.35 (1.63,6.90)**	**2.98 (1.15,7.75)**	**2.78 (1.37,5.62)**	**2.61 (1.39,4.89)**	**2.25 (1.72,2.95)**	2.67 (0.35,20.51)	2.03 (0.86,4.77)	2.01 (0.77,5.23)	1.84 (0.79,4.29)	1.62 (0.97,2.72)	1.64 (0.86,3.11)	1.61 (0.68,3.81)	1.60 (0.85,3.03)	1.20 (0.16,8.80)	1.21 (0.94,1.54)	1.14 (0.88,1.48)	1.08 (0.47,2.48)	1.12 (0.86,1.45)	1.07 (0.84,1.38)	**Placebo**

Figure S17. Results of network meta - analysis of treatment measures grouped by dosing for BICLA response

**Deucravacitinib3mg**																
1.40 (0.44,4.47)	**Deucravacitinib12mg**															
1.80 (0.55,5.89)	1.29 (0.43,3.82)	**Deucravacitinib6mg**														
3.58 (0.34,38.04)	2.56 (0.25,25.95)	1.99 (0.19,20.45)	**Anifrolumab150mg**													
3.81 (0.39,37.13)	2.73 (0.29,25.28)	2.12 (0.23,19.93)	1.06 (0.08,14.70)	**Upadacitinib30mg**												
4.03 (0.70,23.14)	2.89 (0.54,15.51)	2.25 (0.41,12.28)	1.13 (0.13,9.99)	1.06 (0.13,8.53)	**Sifalimumab1200mg**											
4.27 (0.97,18.77)	3.06 (0.75,12.42)	2.38 (0.57,9.87)	1.19 (0.17,8.29)	1.12 (0.17,7.27)	1.06 (0.33,3.40)	**Anifrolumab300mg**										
4.35 (0.79,23.91)	3.12 (0.61,16.00)	2.42 (0.46,12.68)	1.22 (0.14,10.42)	1.14 (0.15,8.88)	1.08 (0.37,3.16)	1.02 (0.34,3.06)	**Sifalimumab200mg**									
4.39 (0.78,24.77)	3.14 (0.60,16.59)	2.44 (0.45,13.14)	1.23 (0.14,10.54)	1.15 (0.14,9.16)	1.09 (0.25,4.74)	1.03 (0.37,2.84)	1.01 (0.24,4.17)	**Anifrolumab1000mg**								
4.29 (0.49,37.13)	3.07 (0.37,25.21)	2.39 (0.29,19.89)	1.20 (0.10,14.95)	1.13 (0.10,12.94)	1.06 (0.15,7.52)	1.00 (0.18,5.62)	0.98 (0.14,6.71)	0.98 (0.14,6.81)	**Iberdomide 0.15mg**							
5.27 (0.74,37.41)	3.78 (0.56,25.25)	2.94 (0.43,19.96)	1.47 (0.14,15.54)	1.38 (0.14,13.36)	1.31 (0.23,7.40)	1.23 (0.29,5.34)	1.21 (0.22,6.57)	1.20 (0.22,6.69)	1.23 (0.24,6.36)	**Iberdomide 0.45mg**						
6.35 (0.78,51.91)	4.54 (0.59,35.19)	3.53 (0.45,27.78)	1.77 (0.15,21.07)	1.67 (0.27,10.33)	1.57 (0.24,10.45)	1.49 (0.29,7.73)	1.46 (0.23,9.30)	1.45 (0.22,9.46)	1.48 (0.15,14.47)	1.20 (0.15,9.74)	**ABBV-599 high-dose**					
**6.35 (1.17,34.53)**	4.55 (0.89,23.09)	3.54 (0.68,18.30)	1.78 (0.21,15.07)	1.67 (0.22,12.85)	1.57 (0.55,4.54)	1.49 (0.50,4.39)	1.46 (0.54,3.91)	1.45 (0.35,5.91)	1.48 (0.22,10.00)	1.20 (0.22,6.46)	1.00 (0.16,6.32)	**Sifalimumab600mg**				
**11.43 (2.84,46.03)**	**8.18 (2.21,30.31)**	**6.36 (1.68,24.12)**	3.20 (0.47,21.58)	3.00 (0.50,18.17)	2.83 (0.99,8.13)	**2.67 (1.63,4.40)**	2.63 (0.99,6.99)	2.60 (0.93,7.27)	2.67 (0.51,13.88)	2.17 (0.55,8.59)	1.80 (0.37,8.68)	1.80 (0.69,4.71)	**Placebo**			
**12.02 (2.67,54.07)**	**8.61 (2.07,35.83)**	**6.69 (1.57,28.47)**	3.36 (0.46,24.63)	3.16 (0.48,20.84)	2.98 (0.90,9.85)	**2.81 (1.32,5.97)**	2.76 (0.89,8.56)	2.74 (0.85,8.84)	2.81 (0.49,16.04)	2.28 (0.51,10.10)	1.89 (0.36,10.07)	1.89 (0.62,5.78)	1.05 (0.60,1.85)	**Baricitinib4mg**		
**12.64 (2.82,56.69)**	**9.05 (2.18,37.56)**	**7.04 (1.66,29.84)**	3.53 (0.48,25.85)	3.32 (0.50,21.86)	3.13 (0.95,10.32)	**2.96 (1.40,6.25)**	2.90 (0.94,8.97)	2.88 (0.90,9.26)	2.95 (0.52,16.82)	2.40 (0.54,10.59)	1.99 (0.38,10.56)	1.99 (0.65,6.06)	1.11 (0.63,1.93)	1.05 (0.59,1.87)	**Baricitinib 2mg**	
**14.29 (2.05,99.47)**	**10.23 (1.56,67.11)**	**7.95 (1.19,53.05)**	3.99 (0.38,41.44)	3.75 (0.39,35.63)	3.54 (0.64,19.64)	3.34 (0.79,14.10)	3.28 (0.62,17.41)	3.25 (0.60,17.75)	3.33 (0.66,16.85)	2.71 (0.71,10.36)	2.25 (0.28,17.89)	2.25 (0.43,11.82)	1.25 (0.32,4.83)	1.19 (0.27,5.14)	1.13 (0.26,4.87)	**Iberdomide 0.30mg**

Figure S18. Results of network meta - analysis of treatment measures grouped by dosing for CLASI-50

**Deucravacitinib3mg**							
1.17 (0.41,3.30)	**Upadacitinib30mg**						
1.63 (0.86,3.09)	1.40 (0.48,4.03)	**Deucravacitinib12mg**					
1.77 (0.63,4.99)	1.52 (0.76,3.04)	1.09 (0.38,3.12)	**ABBV-599high-dose**				
1.84 (0.97,3.49)	1.57 (0.54,4.54)	1.12 (0.57,2.21)	1.04 (0.36,2.98)	**Deucravacitinib6mg**			
**2.96 (1.35,6.48)**	**2.53 (1.17,5.47)**	1.81 (0.80,4.08)	1.67 (0.78,3.57)	1.61 (0.71,3.63)	**Baricitinib 4mg**		
**3.45 (1.57,7.57)**	**2.95 (1.37,6.39)**	2.11 (0.94,4.76)	1.95 (0.91,4.17)	1.88 (0.83,4.25)	1.17 (0.91,1.50)	**Baricitinib 2mg**	
**3.70 (1.76,7.77)**	**3.17 (1.53,6.55)**	**2.27 (1.05,4.90)**	**2.09 (1.02,4.28)**	2.01 (0.93,4.36)	1.25 (0.97,1.61)	1.07 (0.83,1.39)	**Placebo**

Figure S19. Results of network meta - analysis of treatment measures grouped by dosing for LLDAS

Epratuzumab100mg																														
0.25 (0.01.10.42)	Cenerimod4mg																													
0.22 (0.01.9.20)	0.87 (0.18.4.34)	Cenerimod0.5mg																												
0.22 (0.01.9.20)	0.88 (0.18.4.34)	1.00 (0.20.5.07)	Cenerimod1mg																											
0.43 (0.03.5.74)	1.69 (0.04.74.74)	1.93 (0.04.86.20)	1.93 (0.04.86.19)	Epratuzumab1200mg																										
0.19 (0.00.7.54)	0.73 (0.15.3.47)	0.83 (0.17.4.06)	0.83 (0.17.4.06)	0.43 (0.01.19.01)	Cenerimod2mg																									
0.14 (0.00.4.38)	0.56 (0.12.2.57)	0.64 (0.14.3.01)	0.64 (0.14.3.01)	0.33 (0.01.11.12)	0.77 (0.17.3.46)	Belimumab200mg																								
0.15 (0.00.4.99)	0.59 (0.11.3.22)	0.67 (0.12.3.76)	0.67 (0.12.3.76)	0.35 (0.01.12.65)	0.80 (0.15.4.35)	1.05 (0.41.2.67)	Deucravacitinib12mg																							
0.15 (0.00.5.70)	0.60 (0.09.4.07)	0.69 (0.10.4.73)	0.69 (0.10.4.73)	0.36 (0.01.14.41)	0.83 (0.12.5.49)	1.08 (0.30.3.83)	1.03 (0.24.4.51)	Belimumab4mg/kg																						
0.12 (0.00.3.59)	0.47 (0.11.2.06)	0.53 (0.12.2.41)	0.53 (0.12.2.41)	0.28 (0.01.9.12)	0.64 (0.15.2.77)	0.83 (0.55.1.26)	0.80 (0.34.1.89)	0.77 (0.23.2.58)	Belimumab10mg/kg																					
0.12 (0.00.3.99)	0.47 (0.08.2.59)	0.53 (0.09.3.02)	0.53 (0.09.3.02)	0.28 (0.01.10.10)	0.64 (0.12.3.49)	0.83 (0.32.2.16)	0.79 (0.35.1.82)	0.77 (0.17.3.42)	1.00 (0.41.2.42)	Deucravacitinib6mg																				
0.11 (0.00.3.35)	0.44 (0.10.1.92)	0.50 (0.11.2.24)	0.50 (0.11.2.24)	0.26 (0.01.8.52)	0.60 (0.14.2.58)	0.78 (0.53.1.15)	0.75 (0.32.1.75)	0.72 (0.22.2.42)	0.94 (0.81.1.09)	0.94 (0.39.2.25)	Placebo																			
0.11 (0.00.3.72)	0.44 (0.08.2.37)	0.51 (0.09.2.76)	0.51 (0.09.2.76)	0.26 (0.01.9.42)	0.61 (0.12.3.19)	0.79 (0.33.1.91)	0.75 (0.24.2.41)	0.73 (0.17.3.10)	0.95 (0.42.2.13)	0.95 (0.29.3.10)	1.01 (0.46.2.24)	Sifalimumab1200mg																		
0.11 (0.00.3.31)	0.42 (0.09.1.94)	0.48 (0.10.2.27)	0.48 (0.10.2.27)	0.25 (0.01.8.41)	0.58 (0.13.2.61)	0.76 (0.44.1.30)	0.72 (0.28.1.83)	0.70 (0.21.2.38)	0.91 (0.62.1.32)	0.91 (0.35.2.36)	0.97 (0.66.1.41)	0.96 (0.40.2.30)	Belimumab1mg/kg																	
0.11 (0.00.3.35)	0.44 (0.01.35.18)	0.50 (0.01.40.52)	0.50 (0.01.40.52)	0.26 (0.01.8.52)	0.60 (0.01.47.92)	0.78 (0.01.49.58)	0.75 (0.01.50.64)	0.72 (0.01.53.57)	0.94 (0.02.58.53)	0.94 (0.01.64.12)	1.00 (0.02.62.25)	0.99 (0.01.66.43)	1.03 (0.02.65.43)	Epratuzumab400mg																
0.11 (0.00.3.35)	0.44 (0.01.35.18)	0.50 (0.01.40.52)	0.50 (0.01.40.52)	0.26 (0.01.8.52)	0.60 (0.01.47.92)	0.78 (0.01.49.58)	0.75 (0.01.50.64)	0.72 (0.01.53.57)	0.94 (0.02.58.53)	0.94 (0.01.64.12)	1.00 (0.02.62.25)	0.99 (0.01.66.43)	1.03 (0.02.65.43)	1.00 (0.02.62.30)	Epratuzumab600mg															
0.08 (0.00.2.83)	0.33 (0.06.1.82)	0.38 (0.07.2.12)	0.38 (0.07.2.12)	0.20 (0.01.7.17)	0.46 (0.09.2.45)	0.60 (0.24.1.50)	0.57 (0.17.1.88)	0.55 (0.13.2.40)	0.71 (0.30.1.67)	0.72 (0.21.2.41)	0.76 (0.33.1.76)	0.75 (0.33.1.74)	0.79 (0.31.1.97)	0.76 (0.01.51.63)	0.76 (0.01.51.63)	Sifalimumab600mg														
0.08 (0.00.2.83)	0.33 (0.06.1.82)	0.38 (0.07.2.12)	0.38 (0.07.2.12)	0.20 (0.01.7.17)	0.46 (0.09.2.45)	0.60 (0.24.1.50)	0.57 (0.17.1.88)	0.55 (0.13.2.40)	0.71 (0.30.1.67)	0.72 (0.21.2.41)	0.76 (0.33.1.76)	0.75 (0.33.1.74)	0.79 (0.31.1.97)	0.76 (0.01.51.63)	0.76 (0.01.51.63)	1.00 (0.41.2.42)	Sifalimumab200mg													
0.08 (0.00.2.43)	0.30 (0.06.1.48)	0.34 (0.07.1.73)	0.34 (0.07.1.73)	0.18 (0.01.6.15)	0.41 (0.08.2.00)	0.54 (0.26.1.11)	0.51 (0.18.1.46)	0.50 (0.13.1.92)	0.64 (0.34.1.21)	0.64 (0.22.1.87)	0.68 (0.37.1.27)	0.68 (0.25.1.85)	0.71 (0.34.1.45)	0.68 (0.01.44.65)	0.68 (0.01.44.65)	0.90 (0.32.2.54)	0.90 (0.32.2.54)	Anifrolumab150mg												
0.07 (0.00.2.44)	0.29 (0.05.1.57)	0.33 (0.06.1.83)	0.33 (0.06.1.83)	0.17 (0.00.6.20)	0.40 (0.07.2.11)	0.52 (0.21.1.28)	0.49 (0.15.1.61)	0.48 (0.11.2.06)	0.62 (0.27.1.43)	0.62 (0.19.2.06)	0.66 (0.29.1.50)	0.65 (0.21.2.05)	0.68 (0.28.1.69)	0.66 (0.01.44.66)	0.66 (0.01.44.66)	0.87 (0.27.2.81)	0.87 (0.27.2.81)	0.97 (0.35.2.70)	Iberdomide0.15mg											
0.06 (0.00.2.08)	0.24 (0.04.1.36)	0.28 (0.05.1.58)	0.28 (0.05.1.58)	0.14 (0.00.5.27)	0.33 (0.06.1.83)	0.43 (0.16.1.14)	0.41 (0.12.1.41)	0.40 (0.09.1.79)	0.52 (0.21.1.28)	0.52 (0.15.1.81)	0.55 (0.23.1.34)	0.55 (0.17.1.80)	0.57 (0.22.1.50)	0.55 (0.01.37.89)	0.55 (0.01.37.89)	0.73 (0.21.2.47)	0.73 (0.21.2.47)	0.81 (0.27.2.38)	0.84 (0.25.2.81)	Ustekinumab										
0.06 (0.00.1.91)	0.24 (0.05.1.12)	0.28 (0.06.1.31)	0.28 (0.06.1.31)	0.15 (0.00.4.86)	0.34 (0.07.1.51)	0.44 (0.26.0.74)	0.42 (0.17.1.05)	0.41 (0.12.1.43)	0.52 (0.36.0.77)	0.53 (0.20.1.35)	**0.56 (0.39.0.80)**	0.55 (0.23.1.32)	0.58 (0.34.0.97)	0.56 (0.01.35.43)	0.56 (0.01.35.43)	0.74 (0.30.1.83)	0.74 (0.30.1.83)	0.82 (0.43.1.55)	0.85 (0.35.2.08)	1.01 (0.39.2.64)	Anifrolumab300mg									
0.06 (0.00.1.96)	0.24 (0.05.1.21)	0.27 (0.05.1.41)	0.27 (0.05.1.41)	0.14 (0.00.4.97)	0.33 (0.07.1.63)	0.43 (0.20.0.93)	0.41 (0.14.1.20)	0.40 (0.10.1.57)	0.51 (0.26.1.02)	0.51 (0.17.1.55)	0.55 (0.28.1.07)	0.54 (0.19.1.53)	0.57 (0.26.1.22)	0.55 (0.01.35.98)	0.55 (0.01.35.98)	0.72 (0.25.2.10)	0.72 (0.25.2.10)	0.80 (0.33.1.94)	0.83 (0.29.2.39)	0.99 (0.33.3.01)	0.98 (0.49.1.94)	Anifrolumab1000mg								
0.06 (0.00.2.03)	0.23 (0.04.1.39)	0.26 (0.04.1.62)	0.26 (0.04.1.62)	0.13 (0.00.5.14)	0.31 (0.05.1.87)	0.40 (0.13.1.25)	0.38 (0.10.1.50)	0.37 (0.07.1.86)	0.48 (0.16.1.41)	0.48 (0.12.1.92)	0.51 (0.18.1.49)	0.51 (0.13.1.92)	0.53 (0.17.1.64)	0.51 (0.01.36.68)	0.51 (0.01.36.68)	0.68 (0.17.2.62)	0.68 (0.17.2.62)	0.75 (0.22.2.57)	0.78 (0.20.2.99)	0.93 (0.23.3.72)	0.92 (0.30.2.83)	0.94 (0.27.3.31)	IL-2							
0.06 (0.00.1.96)	0.22 (0.04.1.34)	0.25 (0.04.1.56)	0.25 (0.04.1.56)	0.13 (0.00.4.96)	0.30 (0.05.1.81)	0.39 (0.13.1.21)	0.37 (0.09.1.45)	0.36 (0.07.1.80)	0.47 (0.16.1.36)	0.47 (0.12.1.85)	0.50 (0.17.1.44)	0.49 (0.13.1.86)	0.51 (0.17.1.59)	0.50 (0.01.35.43)	0.50 (0.01.35.43)	0.65 (0.17.2.53)	0.65 (0.17.2.53)	0.73 (0.21.2.48)	0.75 (0.20.2.89)	0.90 (0.22.3.59)	0.89 (0.29.2.73)	0.91 (0.26.3.19)	0.97 (0.21.4.35)	Telitacicept80mg						
0.06 (0.00.1.91)	0.23 (0.05.1.19)	0.27 (0.05.1.39)	0.27 (0.05.1.39)	0.14 (0.00.4.85)	0.32 (0.06.1.60)	0.42 (0.19.0.92)	0.40 (0.13.1.19)	0.39 (0.10.1.55)	0.50 (0.25.1.01)	0.50 (0.16.1.53)	0.53 (0.27.1.06)	0.53 (0.18.1.51)	0.55 (0.25.1.21)	0.53 (0.01.35.12)	0.53 (0.01.35.12)	0.70 (0.24.2.07)	0.70 (0.24.2.07)	0.78 (0.31.1.96)	0.81 (0.34.1.91)	0.96 (0.31.2.96)	0.95 (0.44.2.07)	0.97 (0.37.2.54)	1.03 (0.29.3.68)	1.07 (0.30.3.81)	Iberdomide0.45mg					
0.06 (0.00.1.99)	0.22 (0.04.1.35)	0.25 (0.04.1.57)	0.25 (0.04.1.57)	0.13 (0.00.5.03)	0.30 (0.05.1.82)	0.40 (0.13.1.20)	0.38 (0.14.1.03)	0.37 (0.07.1.81)	0.48 (0.17.1.36)	0.48 (0.17.1.33)	0.51 (0.18.1.44)	0.50 (0.14.1.86)	0.52 (0.17.1.58)	0.51 (0.01.35.95)	0.51 (0.01.35.95)	0.67 (0.17.2.53)	0.67 (0.17.2.53)	0.74 (0.22.2.48)	0.77 (0.20.2.89)	0.92 (0.23.3.60)	0.91 (0.30.2.72)	0.93 (0.27.3.19)	0.99 (0.22.4.37)	1.02 (0.23.4.52)	0.95 (0.27.3.32)	Deucravacitinib3mg				
0.06 (0.00.1.88)	0.23 (0.04.1.17)	0.26 (0.05.1.37)	0.26 (0.05.1.37)	0.14 (0.00.4.78)	0.31 (0.06.1.58)	0.41 (0.19.0.90)	0.39 (0.13.1.17)	0.38 (0.09.1.52)	0.49 (0.24.1.00)	0.49 (0.16.1.50)	0.52 (0.26.1.04)	0.52 (0.18.1.48)	0.54 (0.25.1.19)	0.52 (0.01.34.57)	0.52 (0.01.34.57)	0.69 (0.23.2.04)	0.69 (0.23.2.04)	0.77 (0.30.1.93)	0.79 (0.33.1.88)	0.95 (0.31.2.92)	0.94 (0.43.2.04)	0.96 (0.37.2.50)	1.02 (0.29.3.62)	1.05 (0.30.3.75)	0.98 (0.47.2.06)	1.03 (0.30.3.60)	Iberdomide0.30mg			
0.05 (0.00.1.59)	0.19 (0.03.1.03)	0.21 (0.04.1.20)	0.21 (0.04.1.20)	0.11 (0.00.4.02)	0.25 (0.05.1.39)	0.33 (0.13.0.86)	0.32 (0.09.1.07)	0.31 (0.07.1.36)	0.40 (0.16.0.96)	0.40 (0.12.1.37)	0.42 (0.18.1.02)	0.42 (0.13.1.37)	0.44 (0.17.1.13)	0.42 (0.01.28.91)	0.42 (0.01.28.91)	0.56 (0.17.1.87)	0.56 (0.17.1.87)	0.62 (0.21.1.80)	0.64 (0.19.2.13)	0.76 (0.22.2.66)	0.76 (0.29.1.95)	0.77 (0.26.2.33)	0.82 (0.21.3.27)	0.85 (0.21.3.38)	0.80 (0.26.2.43)	0.83 (0.21.3.25)	0.81 (0.26.2.47)	ILT-101		
0.04 (0.00.1.61)	0.17 (0.03.1.12)	0.20 (0.03.1.30)	0.20 (0.03.1.30)	0.10 (0.00.4.06)	0.24 (0.04.1.51)	0.31 (0.10.1.02)	0.30 (0.07.1.22)	0.29 (0.06.1.50)	0.37 (0.12.1.16)	0.38 (0.09.1.56)	0.40 (0.13.1.23)	0.40 (0.10.1.56)	0.41 (0.13.1.35)	0.40 (0.01.28.93)	0.40 (0.01.28.93)	0.52 (0.13.2.13)	0.52 (0.13.2.13)	0.58 (0.16.2.10)	0.60 (0.15.2.43)	0.72 (0.17.3.02)	0.71 (0.22.2.32)	0.73 (0.20.2.70)	0.78 (0.17.3.65)	0.80 (0.23.2.79)	0.75 (0.20.2.81)	0.79 (0.17.3.64)	0.76 (0.20.2.85)	0.94 (0.23.3.92)	Telitacicept160mg	
0.04 (0.00,1.32)	**0.14 (0.02,0.94)**	0.16 (0.02,1.09)	0.16 (0.02,1.09)	0.08 (0.00,3.33)	0.19 (0.03,1.27)	0.25 (0.07,0.89)	0.24 (0.05,1.04)	0.23 (0.04,1.27)	0.30 (0.09,1.01)	0.30 (0.07,1.33)	0.32 (0.10,1.07)	0.32 (0.07,1.34)	0.33 (0.09,1.17)	0.32 (0.00,23.67)	0.32 (0.00,23.67)	0.42 (0.10,1.82)	0.42 (0.10,1.82)	0.47 (0.12,1.81)	0.48 (0.11,2.08)	0.58 (0.13,2.58)	0.57 (0.16,2.01)	0.58 (0.15,2.32)	0.62 (0.12,3.10)	0.64 (0.17,2.40)	0.60 (0.15,2.41)	0.63 (0.13,3.10)	0.61 (0.15,2.45)	0.76 (0.17,3.35)	0.80 (0.20,3.13)	Telitacicept240mg

Figure S20. Results of network meta - analysis of treatment measures grouped by dosing for AE

**IL-2**																																				
0.25 (0.01,5.41)	**Belimumab 10mg/kgIV**																																			
0.24 (0.01,6.33)	0.95 (0.23,3.84)	**Iberdomide 0.30mg**																																		
0.22 (0.01,5.35)	0.88 (0.28,2.80)	0.93 (0.18,4.78)	**deucravacitinib3mg**																																	
0.21 (0.01,4.33)	0.81 (0.39,1.67)	0.86 (0.22,3.35)	0.92 (0.30,2.81)	**Belimumab 200mgSC**																																
0.22 (0.01,5.23)	0.86 (0.27,2.74)	0.91 (0.18,4.66)	0.98 (0.32,2.96)	1.06 (0.35,3.23)	**deucravacitinib12mg**																															
0.32 (0.00,28.10)	1.29 (0.05,36.12)	1.36 (0.04,46.32)	1.46 (0.05,45.58)	1.58 (0.06,43.79)	1.49 (0.05,46.70)	**Cenerimod 2mg**																														
0.20 (0.01,4.48)	0.81 (0.34,1.94)	0.85 (0.20,3.64)	0.92 (0.27,3.11)	0.99 (0.44,2.26)	0.94 (0.28,3.18)	0.63 (0.02,18.04)	**Belimumab 4.0mg/kg**																													
0.32 (0.00,28.10)	1.29 (0.05,36.12)	1.36 (0.04,46.32)	1.46 (0.05,45.58)	1.58 (0.06,43.79)	1.49 (0.05,46.70)	1.00 (0.02,54.56)	1.59 (0.06,45.81)	**Cenerimod 4mg**																												
0.20 (0.01,4.08)	0.77 (0.39,1.52)	0.82 (0.21,3.12)	0.88 (0.30,2.60)	0.95 (0.52,1.74)	0.90 (0.30,2.66)	0.60 (0.02,16.50)	0.96 (0.44,2.09)	0.60 (0.02,16.50)	**Anifrolumab300mg**																											
0.30 (0.00,26.00)	1.19 (0.04,33.45)	1.25 (0.04,42.89)	1.35 (0.04,42.20)	1.46 (0.05,40.55)	1.38 (0.04,43.24)	0.92 (0.02,50.50)	1.47 (0.05,42.42)	0.92 (0.02,50.50)	1.53 (0.06,42.21)	**Cenerimod 0.5mg**																										
0.30 (0.00,26.01)	1.19 (0.04,33.47)	1.25 (0.04,42.91)	1.35 (0.04,42.22)	1.46 (0.05,40.57)	1.38 (0.04,43.26)	0.92 (0.02,50.52)	1.47 (0.05,42.44)	0.92 (0.02,50.52)	1.53 (0.06,42.23)	1.00 (0.02,54.89)	**Cenerimod 1mg**																									
0.19 (0.01,4.28)	0.77 (0.31,1.88)	0.81 (0.19,3.50)	0.87 (0.25,2.99)	0.94 (0.41,2.19)	0.89 (0.26,3.07)	0.60 (0.02,17.24)	0.95 (0.36,2.53)	0.60 (0.02,17.24)	0.99 (0.49,2.00)	0.65 (0.02,18.74)	0.65 (0.02,18.75)	**Anifrolumab150mg**																								
0.20 (0.01,4.69)	0.78 (0.25,2.41)	0.82 (0.16,4.14)	0.89 (0.30,2.60)	0.96 (0.32,2.84)	0.91 (0.31,2.66)	0.61 (0.02,18.77)	0.97 (0.29,3.19)	0.61 (0.02,18.77)	1.01 (0.35,2.89)	0.66 (0.02,20.41)	0.66 (0.02,20.41)	1.02 (0.30,3.40)	**deucravacitinib6mg**																							
0.17 (0.01,4.17)	0.68 (0.21,2.19)	0.72 (0.14,3.73)	0.78 (0.18,3.28)	0.84 (0.27,2.59)	0.80 (0.19,3.36)	0.53 (0.02,16.68)	0.85 (0.25,2.90)	0.53 (0.02,16.68)	0.89 (0.30,2.64)	0.58 (0.02,18.13)	0.58 (0.02,18.14)	0.89 (0.26,3.09)	0.88 (0.21,3.62)	**Telitacicept 80mg**																						
0.17 (0.01,4.17)	0.68 (0.21,2.19)	0.72 (0.14,3.73)	0.78 (0.18,3.28)	0.84 (0.27,2.59)	0.80 (0.19,3.36)	0.53 (0.02,16.68)	0.85 (0.25,2.90)	0.53 (0.02,16.68)	0.89 (0.30,2.64)	0.58 (0.02,18.13)	0.58 (0.02,18.14)	0.89 (0.26,3.09)	0.88 (0.21,3.62)	1.00 (0.34,2.91)	**Telitacicept 240mg**																					
0.16 (0.01,3.36)	0.64 (0.34,1.22)	0.68 (0.18,2.54)	0.73 (0.25,2.11)	0.79 (0.45,1.38)	0.75 (0.26,2.16)	0.50 (0.02,13.60)	0.80 (0.40,1.58)	0.50 (0.02,13.60)	0.83 (0.51,1.37)	0.54 (0.02,14.79)	0.54 (0.02,14.79)	0.84 (0.39,1.81)	0.82 (0.29,2.30)	0.94 (0.32,2.74)	0.94 (0.32,2.74)	**Belimumab 10mg/kg**																				
0.16 (0.01,3.65)	0.65 (0.25,1.65)	0.68 (0.15,3.02)	0.73 (0.21,2.60)	0.80 (0.33,1.92)	0.75 (0.21,2.66)	0.50 (0.02,14.70)	0.80 (0.29,2.21)	0.50 (0.02,14.70)	0.84 (0.36,1.95)	0.55 (0.02,15.98)	0.55 (0.02,15.98)	0.84 (0.30,2.36)	0.83 (0.24,2.85)	0.95 (0.27,3.37)	0.95 (0.27,3.37)	1.01 (0.45,2.28)	**Sifalimumab200mg**																			
0.16 (0.01,3.59)	0.65 (0.27,1.55)	0.68 (0.16,2.91)	0.74 (0.22,2.48)	0.80 (0.35,1.80)	0.75 (0.22,2.55)	0.50 (0.02,14.47)	0.80 (0.31,2.09)	0.50 (0.02,14.47)	0.84 (0.43,1.65)	0.55 (0.02,15.73)	0.55 (0.02,15.73)	0.84 (0.33,2.15)	0.83 (0.25,2.73)	0.95 (0.28,3.22)	0.95 (0.28,3.22)	1.01 (0.48,2.12)	1.00 (0.37,2.74)	**Anifrolumab1,000mg**																		
0.16 (0.01,4.46)	0.63 (0.14,2.90)	0.67 (0.14,3.16)	0.72 (0.13,4.10)	0.78 (0.17,3.46)	0.73 (0.13,4.20)	0.49 (0.01,17.66)	0.78 (0.16,3.78)	0.49 (0.01,17.66)	0.82 (0.19,3.56)	0.53 (0.01,19.19)	0.53 (0.01,19.20)	0.82 (0.17,4.02)	0.81 (0.14,4.54)	0.92 (0.16,5.31)	0.92 (0.16,5.31)	0.98 (0.23,4.21)	0.98 (0.20,4.86)	0.97 (0.20,4.69)	**Iberdomide 0.15mg**																	
0.16 (0.01,3.33)	0.62 (0.30,1.30)	0.66 (0.17,2.59)	0.71 (0.23,2.17)	0.77 (0.40,1.49)	0.73 (0.24,2.23)	0.49 (0.02,13.47)	0.77 (0.34,1.77)	0.49 (0.02,13.47)	0.81 (0.44,1.49)	0.53 (0.02,14.64)	0.53 (0.02,14.65)	0.81 (0.35,1.90)	0.80 (0.27,2.38)	0.91 (0.30,2.82)	0.91 (0.30,2.82)	0.97 (0.55,1.72)	0.96 (0.40,2.35)	0.96 (0.42,2.19)	0.99 (0.22,4.42)	**Tabalumab 120Q2W**																
0.15 (0.01,4.26)	0.61 (0.14,2.73)	0.64 (0.10,4.28)	0.69 (0.12,3.88)	0.75 (0.17,3.25)	0.71 (0.13,3.98)	0.48 (0.01,16.89)	0.76 (0.16,3.56)	0.48 (0.01,16.89)	0.79 (0.19,3.35)	0.51 (0.01,18.36)	0.51 (0.01,18.36)	0.80 (0.17,3.79)	0.78 (0.14,4.29)	0.89 (0.16,5.02)	0.89 (0.16,5.02)	0.95 (0.23,3.96)	0.94 (0.19,4.59)	0.94 (0.20,4.42)	0.97 (0.13,7.06)	0.98 (0.22,4.26)	**Ustekinumab**															
0.15 (0.01,3.86)	0.61 (0.17,2.18)	0.64 (0.17,2.40)	0.69 (0.15,3.20)	0.75 (0.22,2.58)	0.71 (0.15,3.28)	0.47 (0.01,15.41)	0.75 (0.20,2.87)	0.47 (0.01,15.41)	0.79 (0.23,2.65)	0.51 (0.02,16.75)	0.51 (0.02,16.75)	0.79 (0.21,3.06)	0.78 (0.17,3.53)	0.89 (0.19,4.14)	0.89 (0.19,4.14)	0.95 (0.29,3.12)	0.94 (0.24,3.71)	0.94 (0.25,3.56)	0.96 (0.23,4.11)	0.97 (0.28,3.38)	0.99 (0.16,6.06)	**Iberdomide 0.45mg**														
0.13 (0.00,22.70)	0.53 (0.01,35.14)	0.56 (0.01,43.37)	0.60 (0.01,43.40)	0.65 (0.01,42.73)	0.61 (0.01,44.47)	0.41 (0.00,82.57)	0.65 (0.01,44.36)	0.41 (0.00,82.57)	0.68 (0.01,44.57)	0.44 (0.00,89.65)	0.44 (0.00,89.66)	0.69 (0.01,46.83)	0.68 (0.01,48.63)	0.77 (0.01,55.98)	0.77 (0.01,55.98)	0.82 (0.01,53.33)	0.81 (0.01,55.94)	0.81 (0.01,55.13)	0.84 (0.01,67.85)	0.84 (0.01,55.74)	0.86 (0.01,69.54)	0.87 (0.01,65.22)	**Epratuzumab400mg**													
0.13 (0.00,22.69)	0.53 (0.01,35.12)	0.56 (0.01,43.34)	0.60 (0.01,43.38)	0.65 (0.01,42.70)	0.61 (0.01,44.44)	0.41 (0.00,82.53)	0.65 (0.01,44.33)	0.41 (0.00,82.53)	0.68 (0.01,44.54)	0.44 (0.00,89.61)	0.44 (0.00,89.62)	0.69 (0.01,46.80)	0.68 (0.01,48.60)	0.77 (0.01,55.95)	0.77 (0.01,55.95)	0.82 (0.01,53.29)	0.81 (0.01,55.90)	0.81 (0.01,55.09)	0.84 (0.01,67.81)	0.85 (0.01,55.70)	0.86 (0.01,69.50)	0.87 (0.01,65.18)	1.00 (0.02,64.20)	**Epratuzumab100mg**												
0.14 (0.01,3.06)	0.56 (0.24,1.29)	0.59 (0.14,2.46)	0.63 (0.19,2.09)	0.69 (0.32,1.49)	0.65 (0.20,2.14)	0.43 (0.02,12.35)	0.69 (0.33,1.46)	0.43 (0.02,12.35)	0.72 (0.35,1.50)	0.47 (0.02,13.42)	0.47 (0.02,13.43)	0.73 (0.28,1.86)	0.72 (0.22,2.29)	0.82 (0.25,2.70)	0.82 (0.25,2.70)	0.87 (0.46,1.63)	0.86 (0.32,2.29)	0.86 (0.34,2.16)	0.88 (0.19,4.17)	0.89 (0.41,1.96)	0.91 (0.20,4.20)	0.92 (0.25,3.41)	1.06 (0.02,71.19)	1.06 (0.02,71.13)	**Belimumab 1.0mg/kg**											
0.14 (0.01,3.23)	0.54 (0.17,1.65)	0.57 (0.11,2.84)	0.61 (0.15,2.49)	0.66 (0.22,1.95)	0.62 (0.15,2.55)	0.42 (0.01,12.92)	0.67 (0.20,2.19)	0.42 (0.01,12.92)	0.70 (0.24,1.98)	0.45 (0.01,14.04)	0.45 (0.01,14.05)	0.70 (0.21,2.33)	0.69 (0.17,2.75)	0.79 (0.28,2.18)	0.79 (0.28,2.18)	0.84 (0.30,2.33)	0.83 (0.24,2.84)	0.83 (0.25,2.71)	0.85 (0.15,4.77)	0.86 (0.29,2.55)	0.88 (0.16,4.82)	0.89 (0.20,4.01)	1.02 (0.01,73.24)	1.02 (0.01,73.19)	0.96 (0.30,3.07)	**Telitacicept 160mg**										
0.13 (0.01,2.71)	**0.53 (0.30,0.92)**	0.56 (0.15,2.01)	0.60 (0.22,1.65)	0.65 (0.41,1.03)	0.61 (0.22,1.69)	0.41 (0.02,11.00)	0.65 (0.33,1.29)	0.41 (0.02,11.00)	0.68 (0.46,1.00)	0.44 (0.02,11.96)	0.44 (0.02,11.96)	0.69 (0.34,1.39)	0.68 (0.25,1.80)	0.77 (0.28,2.14)	0.77 (0.28,2.14)	0.82 (0.60,1.12)	0.81 (0.38,1.73)	0.81 (0.42,1.59)	0.84 (0.20,3.45)	0.84 (0.53,1.36)	0.86 (0.21,3.47)	0.87 (0.27,2.75)	1.00 (0.02,64.19)	1.00 (0.02,64.15)	0.94 (0.51,1.77)	0.98 (0.37,2.60)	**Placebo**									
0.12 (0.01,2.58)	0.46 (0.19,1.14)	0.49 (0.11,2.11)	0.52 (0.15,1.81)	0.57 (0.24,1.33)	0.54 (0.15,1.85)	0.36 (0.01,10.38)	0.57 (0.21,1.53)	0.36 (0.01,10.38)	0.60 (0.26,1.34)	0.39 (0.01,11.29)	0.39 (0.01,11.29)	0.60 (0.22,1.64)	0.59 (0.18,1.99)	0.67 (0.19,2.35)	0.67 (0.19,2.35)	0.72 (0.33,1.57)	0.71 (0.34,1.49)	0.71 (0.27,1.90)	0.73 (0.15,3.58)	0.74 (0.31,1.74)	0.76 (0.16,3.61)	0.76 (0.20,2.95)	0.87 (0.01,59.64)	0.87 (0.01,59.60)	0.83 (0.32,2.13)	0.86 (0.26,2.87)	0.87 (0.43,1.79)	**Sifalimumab1200mg**								
0.12 (0.01,2.48)	0.47 (0.23,0.95)	0.49 (0.13,1.92)	0.53 (0.17,1.60)	0.57 (0.30,1.09)	0.54 (0.18,1.64)	0.36 (0.01,10.01)	0.58 (0.25,1.31)	0.36 (0.01,10.01)	0.60 (0.33,1.09)	0.39 (0.01,10.88)	0.39 (0.01,10.89)	0.61 (0.26,1.40)	0.60 (0.20,1.76)	0.68 (0.22,2.08)	0.68 (0.22,2.08)	0.72 (0.42,1.26)	0.72 (0.30,1.73)	0.72 (0.32,1.61)	0.74 (0.17,3.27)	0.75 (0.47,1.19)	0.76 (0.18,3.29)	0.77 (0.22,2.64)	0.88 (0.01,58.05)	0.88 (0.01,58.01)	0.83 (0.39,1.80)	0.87 (0.30,2.54)	0.88 (0.56,1.39)	1.01 (0.43,2.35)	**Tabalumab 120Q4W**							
0.12 (0.01,2.46)	0.46 (0.22,0.95)	0.49 (0.12,1.91)	0.52 (0.17,1.60)	0.57 (0.30,1.09)	0.54 (0.18,1.64)	0.36 (0.01,9.96)	0.57 (0.26,1.26)	0.36 (0.01,9.96)	0.60 (0.33,1.09)	0.39 (0.01,10.82)	0.39 (0.01,10.83)	0.60 (0.26,1.40)	0.59 (0.20,1.75)	0.68 (0.22,2.07)	0.68 (0.22,2.07)	0.72 (0.46,1.13)	0.71 (0.30,1.72)	0.71 (0.32,1.61)	0.73 (0.16,3.26)	0.74 (0.38,1.43)	0.76 (0.17,3.28)	0.76 (0.22,2.63)	0.88 (0.01,57.72)	0.88 (0.01,57.68)	0.83 (0.39,1.74)	0.86 (0.29,2.53)	0.88 (0.55,1.39)	1.00 (0.43,2.34)	0.99 (0.52,1.89)	**Belimumab 1mg/kg**						
0.11 (0.01,2.46)	0.44 (0.18,1.08)	0.46 (0.11,2.01)	0.50 (0.14,1.72)	0.54 (0.23,1.26)	0.51 (0.15,1.76)	0.34 (0.01,9.90)	0.55 (0.20,1.46)	0.34 (0.01,9.90)	0.57 (0.25,1.27)	0.37 (0.01,10.76)	0.37 (0.01,10.76)	0.57 (0.21,1.56)	0.56 (0.17,1.89)	0.64 (0.19,2.23)	0.64 (0.19,2.23)	0.68 (0.32,1.49)	0.68 (0.33,1.42)	0.68 (0.26,1.80)	0.70 (0.14,3.41)	0.71 (0.30,1.66)	0.72 (0.15,3.44)	0.72 (0.19,2.80)	0.83 (0.01,56.88)	0.83 (0.01,56.84)	0.79 (0.31,2.03)	0.82 (0.25,2.73)	0.83 (0.41,1.69)	0.95 (0.48,1.91)	0.95 (0.41,2.19)	0.95 (0.41,2.21)	**Sifalimumab600mg**					
0.09 (0.00,2.98)	0.34 (0.05,2.36)	0.36 (0.04,3.44)	0.39 (0.05,3.21)	0.42 (0.06,2.83)	0.40 (0.05,3.28)	0.27 (0.01,11.62)	0.43 (0.06,3.05)	0.27 (0.01,11.62)	0.45 (0.07,2.93)	0.29 (0.01,12.63)	0.29 (0.01,12.63)	0.45 (0.06,3.23)	0.44 (0.05,3.56)	0.50 (0.06,4.14)	0.50 (0.06,4.14)	0.54 (0.08,3.48)	0.53 (0.07,3.89)	0.53 (0.07,3.78)	0.55 (0.05,5.59)	0.55 (0.08,3.70)	0.56 (0.06,5.68)	0.57 (0.06,4.99)	0.65 (0.01,61.91)	0.65 (0.01,61.86)	0.62 (0.09,4.32)	0.64 (0.08,5.16)	0.65 (0.10,4.13)	0.75 (0.10,5.39)	0.74 (0.11,4.94)	0.74 (0.11,4.98)	0.78 (0.11,5.64)	**ILT-101**				
0.03 (0.00,3.47)	0.13 (0.00,4.74)	0.14 (0.00,6.00)	0.15 (0.00,5.94)	0.16 (0.00,5.75)	0.15 (0.00,6.08)	0.10 (0.00,12.85)	0.16 (0.00,6.00)	0.10 (0.00,12.85)	0.17 (0.00,5.99)	0.11 (0.00,13.96)	0.11 (0.00,13.96)	0.17 (0.00,6.34)	0.17 (0.00,6.64)	0.19 (0.00,7.66)	0.19 (0.00,7.66)	0.21 (0.01,7.16)	0.20 (0.01,7.58)	0.20 (0.01,7.46)	0.21 (0.00,9.45)	0.21 (0.01,7.50)	0.22 (0.00,9.67)	0.22 (0.01,8.97)	0.25 (0.01,8.61)	0.25 (0.01,8.60)	0.24 (0.01,8.59)	0.25 (0.01,9.63)	0.25 (0.01,8.60)	0.29 (0.01,10.57)	0.28 (0.01,10.04)	0.29 (0.01,10.11)	0.30 (0.01,11.06)	0.38 (0.01,20.70)	**Epratuzumab1200mg**			
0.03 (0.00,3.47)	0.13 (0.00,4.74)	0.14 (0.00,6.00)	0.15 (0.00,5.94)	0.16 (0.00,5.75)	0.15 (0.00,6.08)	0.10 (0.00,12.85)	0.16 (0.00,6.00)	0.10 (0.00,12.85)	0.17 (0.00,5.99)	0.11 (0.00,13.96)	0.11 (0.00,13.96)	0.17 (0.00,6.34)	0.17 (0.00,6.64)	0.19 (0.00,7.66)	0.19 (0.00,7.66)	0.21 (0.01,7.16)	0.20 (0.01,7.58)	0.20 (0.01,7.46)	0.21 (0.00,9.45)	0.21 (0.01,7.50)	0.22 (0.00,9.67)	0.22 (0.01,8.97)	0.25 (0.01,8.61)	0.25 (0.01,8.60)	0.24 (0.01,8.59)	0.25 (0.01,9.63)	0.25 (0.01,8.60)	0.29 (0.01,10.57)	0.28 (0.01,10.04)	0.29 (0.01,10.11)	0.30 (0.01,11.06)	0.38 (0.01,20.70)	1.00 (0.06,16.10)	**Epratuzumab600mg**		
0.09 (0.00,1.87)	0.35 (0.18,0.71)	0.37 (0.10,1.43)	0.40 (0.13,1.20)	0.43 (0.23,0.81)	0.41 (0.14,1.23)	0.27 (0.01,7.55)	0.44 (0.20,0.97)	0.27 (0.01,7.55)	0.46 (0.26,0.80)	0.30 (0.01,8.21)	0.30 (0.01,8.21)	0.46 (0.20,1.04)	0.45 (0.16,1.31)	0.51 (0.17,1.55)	0.51 (0.17,1.55)	0.55 (0.33,0.92)	0.54 (0.23,1.29)	0.54 (0.25,1.20)	0.56 (0.13,2.45)	0.56 (0.30,1.06)	0.58 (0.14,2.47)	0.58 (0.17,1.98)	0.67 (0.01,43.82)	0.67 (0.01,43.78)	0.63 (0.30,1.34)	0.66 (0.23,1.90)	0.67 (0.44,1.01)	0.76 (0.33,1.75)	0.76 (0.41,1.40)	0.76 (0.41,1.42)	0.80 (0.35,1.82)	1.02 (0.15,6.78)	2.67 (0.08,94.29)	2.67 (0.08,94.29)	**Baricitinib4mg**	
0.08 (0.00,1.74)	0.33 (0.16,0.66)	0.35 (0.09,1.34)	0.37 (0.12,1.12)	0.40 (0.22,0.75)	0.38 (0.13,1.14)	0.26 (0.01,7.03)	0.41 (0.18,0.90)	0.26 (0.01,7.03)	0.42 (0.24,0.75)	0.28 (0.01,7.65)	0.28 (0.01,7.65)	0.43 (0.19,0.97)	0.42 (0.15,1.22)	0.48 (0.16,1.45)	0.48 (0.16,1.45)	0.51 (0.30,0.86)	0.51 (0.22,1.20)	0.51 (0.23,1.12)	0.52 (0.12,2.28)	0.53 (0.28,0.99)	0.54 (0.13,2.30)	0.54 (0.16,1.84)	0.62 (0.01,40.83)	0.62 (0.01,40.80)	0.59 (0.28,1.24)	0.61 (0.21,1.76)	0.62 (0.41,0.94)	0.71 (0.31,1.63)	0.71 (0.38,1.30)	0.71 (0.38,1.32)	0.75 (0.33,1.70)	0.95 (0.14,6.32)	2.49 (0.07,87.85)	2.49 (0.07,87.85)	0.93 (0.64,1.36)	**Baricitinib2mg**

Figure S21. Results of network meta - analysis of treatment measures grouped by dosing for SAE

**IL-2**																							
0.42 (0.07,2.59)	**Belimumab 200mg**																						
0.36 (0.06,2.05)	0.86 (0.43,1.71)	**Emab1200mg**																					
0.36 (0.05,2.41)	0.86 (0.31,2.41)	1.00 (0.42,2.39)	**Ustekinumab**																				
0.32 (0.06,1.81)	0.76 (0.39,1.50)	0.89 (0.60,1.33)	0.89 (0.37,2.11)	**Belimumab 10mg/kg**																			
0.32 (0.06,1.79)	0.75 (0.38,1.49)	0.87 (0.65,1.17)	0.87 (0.36,2.09)	0.98 (0.66,1.46)	**Emab600mg**																		
0.32 (0.05,1.91)	0.75 (0.33,1.74)	0.88 (0.46,1.66)	0.88 (0.32,2.37)	0.98 (0.53,1.85)	1.00 (0.53,1.90)	**Tabalumab 120mgQ2W**																	
0.30 (0.05,1.74)	0.71 (0.33,1.52)	0.83 (0.49,1.40)	0.82 (0.32,2.10)	0.93 (0.55,1.56)	0.95 (0.56,1.60)	0.94 (0.46,1.93)	**Blisibimod200mgQW**																
0.30 (0.05,1.63)	0.70 (0.38,1.31)	0.82 (0.61,1.10)	0.82 (0.36,1.86)	0.92 (0.70,1.21)	0.94 (0.70,1.26)	0.93 (0.53,1.65)	0.99 (0.64,1.54)	**Placebo**															
0.30 (0.01,7.93)	0.71 (0.04,12.47)	0.83 (0.05,13.77)	0.83 (0.04,15.26)	0.93 (0.06,15.45)	0.95 (0.06,15.77)	0.95 (0.05,16.39)	1.00 (0.06,17.01)	1.01 (0.06,16.57)	**deucravacitinib3mg**														
0.29 (0.05,1.79)	0.69 (0.28,1.67)	0.80 (0.40,1.61)	0.80 (0.28,2.26)	0.90 (0.45,1.79)	0.92 (0.46,1.84)	0.92 (0.39,2.14)	0.97 (0.45,2.10)	0.98 (0.52,1.84)	0.97 (0.06,17.03)	**Belimumab 10.0mg/kg**													
0.30 (0.02,4.03)	0.70 (0.09,5.56)	0.82 (0.11,6.02)	0.82 (0.10,6.93)	0.92 (0.13,6.74)	0.94 (0.13,6.89)	0.93 (0.12,7.28)	0.99 (0.13,7.49)	1.00 (0.14,7.19)	0.99 (0.03,30.30)	1.02 (0.13,8.10)	**E6742200mg**												
0.29 (0.01,7.76)	0.70 (0.04,12.20)	0.81 (0.05,13.47)	0.81 (0.04,14.92)	0.91 (0.05,15.11)	0.93 (0.06,15.42)	0.92 (0.05,16.03)	0.98 (0.06,16.63)	0.99 (0.06,16.20)	0.98 (0.06,16.02)	1.01 (0.06,17.74)	0.99 (0.03,30.30)	**deucravacitinib12mg**											
0.29 (0.05,1.73)	0.68 (0.30,1.57)	0.80 (0.42,1.49)	0.80 (0.30,2.15)	0.90 (0.48,1.66)	0.91 (0.49,1.71)	0.91 (0.52,1.60)	0.96 (0.47,1.96)	0.97 (0.56,1.70)	0.96 (0.06,16.64)	0.99 (0.43,2.30)	0.97 (0.13,7.55)	0.98 (0.06,17.02)	**Tabalumab 120mgQ4W**										
0.28 (0.05,1.57)	0.66 (0.34,1.30)	0.77 (0.52,1.14)	0.77 (0.32,1.82)	0.87 (0.59,1.26)	0.88 (0.59,1.31)	0.88 (0.47,1.64)	0.93 (0.56,1.56)	0.94 (0.72,1.22)	0.93 (0.06,15.42)	0.96 (0.48,1.90)	0.94 (0.13,6.88)	0.95 (0.06,15.77)	0.97 (0.52,1.79)	**Baricitinib2mg**									
0.27 (0.04,1.65)	0.63 (0.26,1.54)	0.74 (0.37,1.48)	0.74 (0.26,2.08)	0.83 (0.42,1.65)	0.85 (0.42,1.70)	0.84 (0.36,1.97)	0.90 (0.41,1.93)	0.90 (0.48,1.70)	0.89 (0.05,15.69)	0.92 (0.49,1.74)	0.90 (0.11,7.16)	0.91 (0.05,16.05)	0.93 (0.40,2.15)	0.96 (0.48,1.90)	**Belimumab 1.0mg/kg**								
0.28 (0.05,1.55)	0.65 (0.33,1.28)	0.76 (0.51,1.13)	0.76 (0.32,1.80)	0.86 (0.59,1.25)	0.87 (0.59,1.29)	0.87 (0.47,1.62)	0.92 (0.55,1.54)	0.93 (0.71,1.21)	0.92 (0.06,15.26)	0.95 (0.48,1.88)	0.93 (0.13,6.81)	0.94 (0.06,15.61)	0.96 (0.52,1.77)	0.99 (0.76,1.29)	1.03 (0.52,2.04)	**Baricitinib4mg**							
0.25 (0.02,3.44)	0.59 (0.07,4.78)	0.68 (0.09,5.18)	0.68 (0.08,5.95)	0.77 (0.10,5.80)	0.78 (0.10,5.93)	0.78 (0.10,6.26)	0.83 (0.11,6.44)	0.83 (0.11,6.19)	0.82 (0.03,25.72)	0.85 (0.10,6.96)	0.83 (0.11,6.19)	0.84 (0.03,26.31)	0.86 (0.11,6.86)	0.89 (0.12,6.70)	0.92 (0.11,7.56)	0.90 (0.12,6.77)	**E6742100mg**						
0.27 (0.05,1.55)	0.64 (0.31,1.32)	0.74 (0.46,1.20)	0.74 (0.30,1.84)	0.83 (0.57,1.23)	0.85 (0.52,1.38)	0.85 (0.43,1.68)	0.90 (0.50,1.61)	0.91 (0.62,1.33)	0.90 (0.05,15.08)	0.93 (0.44,1.94)	0.91 (0.12,6.77)	0.92 (0.05,15.43)	0.93 (0.47,1.83)	0.96 (0.61,1.54)	1.00 (0.48,2.10)	0.97 (0.61,1.55)	1.09 (0.14,8.38)	**Belimumab 1mg/kg**					
0.24 (0.04,1.56)	0.57 (0.21,1.53)	0.66 (0.29,1.52)	0.66 (0.21,2.05)	0.74 (0.32,1.69)	0.75 (0.33,1.74)	0.75 (0.29,1.97)	0.80 (0.32,1.96)	0.80 (0.37,1.76)	0.79 (0.04,14.50)	0.82 (0.30,2.24)	0.80 (0.10,6.71)	0.81 (0.04,14.83)	0.83 (0.32,2.16)	0.85 (0.37,1.95)	0.89 (0.33,2.43)	0.86 (0.38,1.97)	0.96 (0.11,8.30)	0.89 (0.37,2.12)	**Telitacicept 80mg**				
0.15 (0.01,2.95)	0.36 (0.03,4.41)	0.42 (0.04,4.83)	0.42 (0.03,5.43)	0.47 (0.04,5.42)	0.48 (0.04,5.53)	0.48 (0.04,5.79)	0.51 (0.04,5.98)	0.51 (0.05,5.80)	0.51 (0.04,5.73)	0.52 (0.04,6.42)	0.51 (0.02,11.68)	0.52 (0.05,5.87)	0.53 (0.04,6.35)	0.54 (0.05,6.26)	0.57 (0.05,6.97)	0.55 (0.05,6.32)	0.61 (0.03,14.31)	0.56 (0.05,6.59)	0.64 (0.05,8.16)	**deucravacitinib6mg**			
0.20 (0.03,1.31)	0.47 (0.17,1.29)	0.55 (0.24,1.28)	0.55 (0.18,1.72)	0.62 (0.27,1.43)	0.63 (0.27,1.47)	0.63 (0.24,1.67)	0.67 (0.27,1.65)	0.67 (0.30,1.49)	0.66 (0.04,12.16)	0.69 (0.25,1.89)	0.67 (0.08,5.64)	0.68 (0.04,12.44)	0.69 (0.26,1.82)	0.71 (0.31,1.65)	0.74 (0.27,2.05)	0.72 (0.31,1.67)	0.81 (0.09,6.97)	0.74 (0.31,1.79)	0.84 (0.37,1.88)	1.31 (0.10,16.92)	**Telitacicept 160mg**		
0.20 (0.03,1.28)	0.49 (0.20,1.20)	0.57 (0.28,1.16)	0.57 (0.20,1.62)	0.64 (0.31,1.30)	0.65 (0.32,1.33)	0.65 (0.27,1.54)	0.69 (0.31,1.51)	0.69 (0.36,1.33)	0.68 (0.04,12.09)	0.71 (0.36,1.37)	0.69 (0.09,5.53)	0.70 (0.04,12.37)	0.71 (0.30,1.68)	0.74 (0.36,1.49)	0.77 (0.39,1.49)	0.74 (0.37,1.51)	0.83 (0.10,6.84)	0.76 (0.36,1.63)	0.86 (0.31,2.39)	1.35 (0.11,16.74)	1.03 (0.37,2.89)	**Belimumab 4.0mg/kg**	
0.17 (0.03,1.14)	0.41 (0.15,1.13)	0.48 (0.20,1.13)	0.47 (0.15,1.51)	0.53 (0.23,1.26)	0.54 (0.23,1.29)	0.54 (0.20,1.46)	0.58 (0.23,1.45)	0.58 (0.26,1.31)	0.57 (0.03,10.55)	0.59 (0.21,1.66)	0.58 (0.07,4.90)	0.59 (0.03,10.79)	0.60 (0.22,1.60)	0.62 (0.26,1.45)	0.64 (0.23,1.80)	0.62 (0.27,1.47)	0.70 (0.08,6.06)	0.64 (0.26,1.57)	0.72 (0.32,1.65)	1.14 (0.09,14.69)	0.86 (0.37,1.99)	0.84 (0.30,2.39)	**Telitacicept 240mg**

Figure S22. Results of network meta-analysis of treatment measures grouped by dosing for infection related AEs.

Notes: #: Composite responder index based on improvement in disease activity (at least 4 point improvement in SELENA-SLEDAI) without worsening of the overall condition (no worsening in PGA) or the development of significant disease activity in new organ systems (no new BILAG A or >1 new BILAG B). ##: Composite responder index based on improvement in disease activity (at least 4 point improvement in SLEDAI-2K score) without worsening of the overall condition (no worsening in PGA) or the development of significant disease activity in new organ systems (no new BILAG A or >1 new BILAG B). Estimates are presented as relative risk (RR) and 95% confidence intervals (CI; in parentheses). Comparisons should be read from left to right and the estimate is in the cell in common between the column-defining treatment and the row-defining treatment. If the 95% CI of the RR did not contain 1, the differences were considered statistically significant. Significant results are presented in bold.

Appendix 8 funnel plot

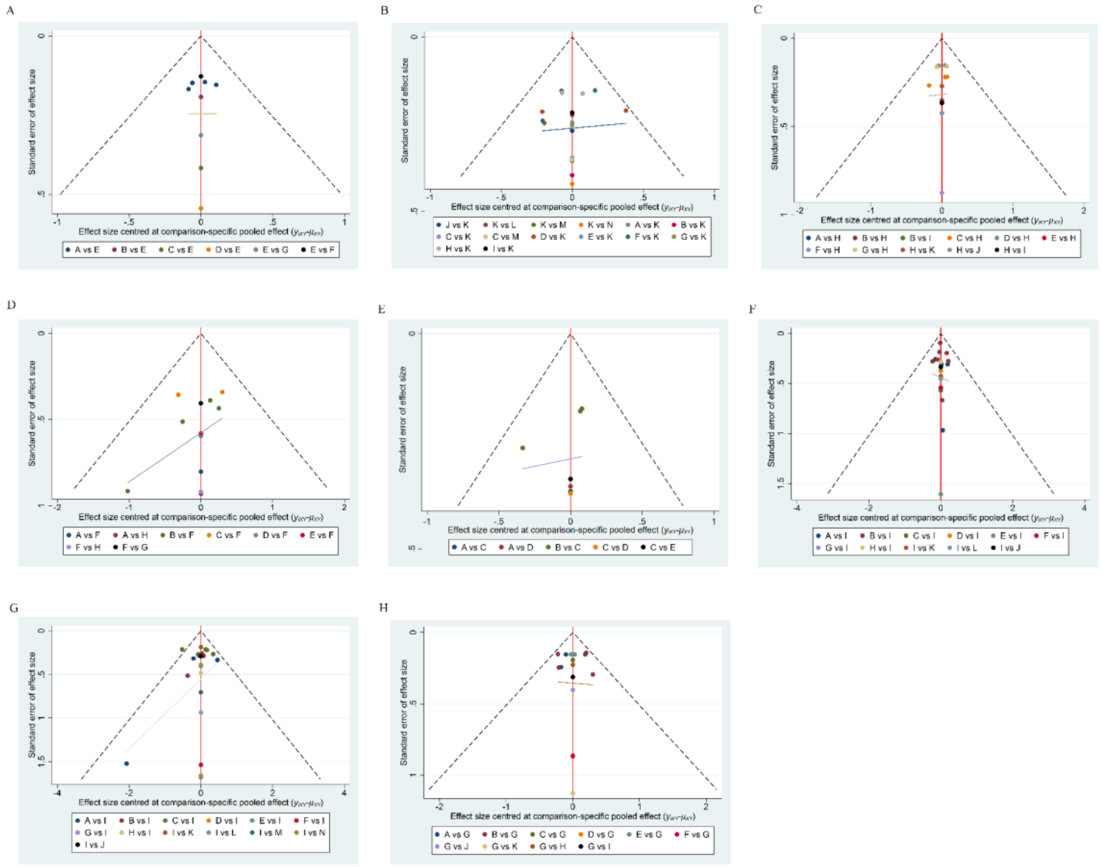

Figure S23 funnel plot for (**A**) SRI-4 response #, (**B**) SRI-4 response ##, (**C**) BICLA response, (**D**) CLASI-50, (**E**) LLDAS, (F) AE, (G) SAEs, and (H) Infection related AEs.

Notes: #: Composite responder index based on improvement in disease activity (at least 4 point improvement in SELENA-SLEDAI) without worsening of the overall condition (no worsening in PGA) or the development of significant disease activity in new organ systems (no new BILAG A or >1 new BILAG B). ##: Composite responder index based on improvement in disease activity (at least 4 point improvement in SLEDAI-2K score) without worsening of the overall condition (no worsening in PGA) or the development of significant disease activity in new organ systems (no new BILAG A or >1 new BILAG B).

Table S22 Egger's test

outcome	study	Egger's test *p*值
SRI-4#	9	/
SRI-4##	17	0.6612
BICLA	13	0.6910
CLASI-50	10	0.2443
LLDAS	5	/
AE	20	0.3614
SAE	22	0.6143
Infection related AEs	16	0.2440

Table S23 SUCRA rankings for SRI-4#: leave-one-out sensitivity analysis

Treatment	SUCRA（%）	SUCRA Rank	Exclude study
Telitacicept	95.17	1	1
IL2	82.17	2	1
Belimumab	61.83	3	1
Tabalumab	44.33	4	1
ILT101	34.83	5	1
Blisibimod	24.67	6	1
Placebo	7.00	7	1
Telitacicept	94.17	1	2
IL2	84.83	2	2
Belimumab	59.17	3	2
Tabalumab	42.67	4	2
ILT101	35.17	5	2
Blisibimod	29.17	6	2
Placebo	4.83	7	2
Telitacicept	95.83	1	3
IL2	80.17	2	3
Belimumab	61.17	3	3
Tabalumab	46.17	4	3
ILT101	34.67	5	3
Blisibimod	26.50	6	3
Placebo	5.50	7	3
Telitacicept	95.00	1	4
IL2	81.67	2	4
Belimumab	59.33	3	4
Tabalumab	44.83	4	4
ILT101	36.33	5	4
Blisibimod	27.00	6	4
Placebo	5.83	7	4
Telitacicept	100.00	1	5
Belimumab	68.20	2	5
ILT101	48.60	3	5
Tabalumab	48.60	3	5
Blisibimod	29.00	5	5
Placebo	5.60	6	5
Telitacicept	94.20	1	6
IL2	80.20	2	6
Belimumab	58.20	3	6
Tabalumab	38.80	4	6
Blisibimod	24.80	5	6
Placebo	3.80	6	6
Telitacicept	94.80	1	7
IL2	78.20	2	7
Belimumab	53.60	3	7

Tabalumab	35.60	4	7
ILT101	34.40	5	7
Placebo	3.40	6	7
Telitacicept	93.60	1	8
IL2	80.40	2	8
Belimumab	54.40	3	8
ILT101	40.40	4	8
Blisibimod	27.00	5	8
Placebo	4.20	6	8
IL2	91.80	1	9
Belimumab	71.20	2	9
Tabalumab	51.80	3	9
ILT101	48.80	4	9
Blisibimod	30.00	5	9
Placebo	6.40	6	9

Table S24 SUCRA rankings for LLDAS: leave-one-out sensitivity analysis.

Treatment	SUCRA（%）	SUCRA Rank	Exclude study
Upadacitinib	94.67	1	1
ABBV599highdose	69.00	2	1
Baricitinib	27.67	3	1
Placebo	8.67	4	1
Upadacitinib	83.25	1	2
Deucravacitinib	76.00	2	2
ABBV599highdose	59.75	3	2
Baricitinib	24.75	4	2
Placebo	6.25	5	2
Upadacitinib	81.50	1	3
Deucravacitinib	79.75	2	3
ABBV599highdose	57.00	3	3
Baricitinib	27.50	4	3
Placebo	4.25	5	3
Upadacitinib	84.75	1	4
Deucravacitinib	81.00	2	4
ABBV599highdose	58.25	3	4
Baricitinib	16.75	4	4
Placebo	9.25	5	4
Deucravacitinib	100.00	1	5
Baricitinib	47.00	2	5
Placebo	3.00	3	5

Table S25 Heterogeneity test results

outcome	τ^2	τ	I^2（95% CI）	Cochran’s Q（df）	*P*-value

SRI-4#	0.0000	0.0000	0% (0–84.7%)	0.40 (3)	0.941
SRI-4##	0.0031	0.0560	22.2% (0–66.5%)	6.43 (5)	0.267
BICLA	0.0000	0.0000	0% (0–79.2%)	1.20 (4)	0.878
CLASI-50	0.0000	0.0000	0% (0–79.2%)	2.65 (4)	0.618
LLDAS	0.0000	0.0000	0% (0–89.6%)	1.74 (2)	0.42
AE	0.0000	0.0000	0% (0–60.2%)	5.10 (10)	0.884
SAE	0.0289	0.1699	32.8% (0-68%)	13.4 (9)	0.145
Infection related AEs	0.0028	0.0530	28.9% (0-69.4%)	8.44 (6)	0.208

Appendix 9 Risk of bias (RoB) assessment

Table S26 Risk of bias (RoB) assessment

First author, year ofpublication	Random sequence generation	Allocation concealment	Blinding of participants and personnel	Blinding of outcome assessment	Incomplete outcome data	Selective reporting	Other bias
Eric Morand,2023	unclear risk	unclear risk	low risk	low risk	low risk	low risk	low risk
Eric F Morand, 2023	low risk	low risk	low risk	low risk	low risk	low risk	low risk
Michelle Petri, 2023	low risk	low risk	low risk	low risk	low risk	low risk	low risk
Daniel J Wallace, 2018	low risk	low risk	low risk	low risk	low risk	low risk	low risk
Joan T Merrill, 2024	low risk	low risk	low risk	low risk	low risk	low risk	low risk
Richard A Furie, 2021	low risk	unclear risk	unclear risk	unclear risk	low risk	low risk	low risk
Daniel J Wallace, 2009	unclear risk	unclear risk	unclear risk	unclear risk	low risk	low risk	low risk
Richard A Furie, 2019	low risk	low risk	low risk	low risk	low risk	low risk	low risk
Eric F Morand, 2020	unclear risk	unclear risk	unclear risk	unclear risk	high risk	low risk	low risk
Sandra V Navarra, 2011	low risk	low risk	low risk	low risk	low risk	low risk	low risk
Richard Furie, 2011	low risk	low risk	low risk	low risk	low risk	low risk	low risk
William Stohl, 2017	unclear risk	high risk	unclear risk	unclear risk	low risk	low risk	low risk
Fengchun Zhang, 2018	low risk	low risk	low risk	low risk	low risk	low risk	low risk
Joan T Merrill, 2018a	low risk	unclear risk	unclear risk	unclear risk	low risk	low risk	low risk
Ronald F van Vollenhoven, 2018	low risk	low risk	low risk	low risk	low risk	low risk	low risk
Richard Furie, 2017	unclear risk	unclear risk	unclear risk	unclear risk	low risk	low risk	low risk
Ellen Ginzler, 2022	low risk	low risk	low risk	low risk	low risk	low risk	low risk
Munther Khamashta, 2016	low risk	low risk	low risk	low risk	low risk	low risk	low risk
Ian N Bruce, 2021	low risk	low risk	low risk	low risk	low risk	low risk	low risk
Di Wu, 2024	unclear risk	low risk	low risk	low risk	low risk	low risk	low risk
Viktoria Hermann, 2019	low risk	low risk	low risk	low risk	low risk	low risk	low risk
Daniel J. Wallace, 2023	low risk	low risk	low risk	low risk	low risk	low risk	low risk
Joan T. Merrill, 2022	unclear risk	unclear risk	unclear risk	unclear risk	low risk	low risk	low risk
Tomomi Tsuru, 2016	unclear risk	low risk	low risk	low risk	low risk	low risk	low risk
Megan E B Clowse, 2017	low risk	unclear risk	unclear risk	unclear risk	low risk	low risk	low risk
Saira Z Sheikh, 2021	low risk	low risk	low risk	low risk	low risk	low risk	low risk
Jing He, 2020	unclear risk	low risk	low risk	low risk	low risk	low risk	low risk

Jens Y Humrich, 2022	low risk	low risk	low risk	low risk	low risk	low risk	low risk
Joan T Merrill, 2018b	low risk	low risk	low risk	low risk	low risk	low risk	low risk
D A Isenberg, 2015	unclear risk	unclear risk	low risk	low risk	low risk	low risk	low risk
Yoshiya Tanaka, 2024	low risk	low risk	low risk	low risk	low risk	low risk	low risk
Daniel J Wallace, 2017	low risk	unclear risk	unclear risk	unclear risk	low risk	low risk	low risk

	Random sequence generation (selection bias)	Allocation concealment (selection bias)	Blinding of participants and personnel (performance bias)	Blinding of outcome assessment (detection bias)	Incomplete outcome data (attrition bias)	Selective reporting (reporting bias)	Other bias
D A Isenberg,2015	?	?	+	+	+	+	+
Daniel J. Wallace,2023	+	+	+	+	+	+	+
Daniel J Wallace,2009	?	?	?	?	+	+	+
Daniel J Wallace,2017	+	?	?	?	+	+	+
Daniel J Wallace,2018	+	+	+	+	+	+	+
Di Wu ,2024	?	+	+	+	+	+	+
Ellen Ginzler ,2022	+	+	+	+	+	+	+
Eric F Morand,2020	?	?	?	?	−	+	+
Eric F Morand,2023	+	+	+	+	+	+	+
Eric Morand,2023	?	?	+	+	+	+	+
Fengchun Zhang,2018	+	+	+	+	+	+	+
Ian N Bruce,2021	+	+	+	+	+	+	+
Jens Y Humrich,2022	+	+	+	+	+	+	+
Jing He,2020	?	+	+	+	+	+	+
Joan T. Merrill,2022	?	?	?	?	+	+	+
Joan T Merrill,2018a	+	?	?	?	+	+	+
Joan T Merrill ,2018b	+	+	+	+	+	+	+
Joan T Merrill,2024	+	+	+	+	+	+	+
Megan E B Clowse,2017	+	?	?	?	+	+	+
Michelle Petri,2023	+	+	+	+	+	+	+
Munther Khamashta,2016	+	+	+	+	+	+	+
Richard A Furie,2019	+	+	+	+	+	+	+
Richard A Furie,2021	+	?	?	?	+	+	+
Richard Furie,2017	?	?	?	?	+	+	+
Richard Furie 2011	+	+	+	+	+	+	+
Ronald F van Vollenhoven,2018	+	+	+	+	+	+	+
Saira Z Sheikh,2021	+	+	+	+	+	+	+
Sandra V Navarra,2011	+	+	+	+	+	+	+
Tomomi Tsuru,2016	?	+	+	+	+	+	+
Viktoria Hermann,2019	+	+	+	+	+	+	+
William Stohl ,2017	?	−	?	?	+	+	+
Yoshiya Tanaka,2024	+	+	+	+	+	+	+

Figure S24 Risk of bias summary of all included studies

Appendix 10 GRADE Assessment

Table S27 GRADE Assessment for SRI-4#.

Treatment regimen	Nature of evidence	Quality grade	Downgrading factor
Telitacicept vs IL-2	Indirect	low	Indirectness[1];inaccuracy[2]
Telitacicept vs Belimumab	Indirect	Medium	Indirectness[1]
Telitacicept vs ILT-101	Indirect	Medium	Indirectness[1]
Telitacicept vs Tabalumab	Indirect	Medium	Indirectness[1]
Telitacicept vs Blisibimod	Indirect	Medium	Indirectness[1]
Telitacicept vs Placebo	Direct	high	none
IL-2 vs Belimumab	Indirect	low	Indirectness[1];inaccuracy[2]
IL-2 vs ILT-101	Indirect	low	Indirectness[1];inaccuracy[2]
IL-2 vs Tabalumab	Indirect	low	Indirectness[1];inaccuracy[2]
IL-2 vs Blisibimod	Indirect	low	Indirectness[1];inaccuracy[2]
IL-2 vs Placebo	Direct	high	none
Belimumab vs ILT-101	Indirect	low	Indirectness[1];inaccuracy[2]
Belimumab vs Tabalumab	Indirect	low	Indirectness[1];inaccuracy[2]
Belimumab vs Blisibimod	Indirect	low	Indirectness[1];inaccuracy[2]
Belimumab vs Placebo	Direct	high	none
ILT-101 vs Tabalumab	Indirect	low	Indirectness[1];inaccuracy[2]
ILT-101 vs Blisibimod	Indirect	low	Indirectness[1];inaccuracy[2]
ILT-101 vs Placebo	Direct	Medium	inaccuracy[2]
Tabalumab vs Blisibimod	Indirect	low	Indirectness[1];inaccuracy[2]
Tabalumab vs Placebo	Direct	high	none
Blisibimod vs Placebo	Direct	Medium	inaccuracy[2]

notes:1: No head-to-head RCTs, evidence derived from indirect comparisons via placebo; 2: 95% CI crossing the null line.

Table S28 GRADE Assessment for SRI-4##.

Treatment regimen	Nature of evidence	Quality grade	Downgrading factor
Ustekinumab vs ABBV-599highdose	Indirect	low	Indirectness[1]; inaccuracy[2]
Ustekinumab vs Deucravacitinib	Indirect	low	Indirectness[1]; inaccuracy[2]
Ustekinumab vs Anifrolumab	Indirect	low	Indirectness[1]; inaccuracy[2]
Ustekinumab vs Upadacitinib	Indirect	low	Indirectness[1]; inaccuracy[2]
Ustekinumab vs Sifalimumab	Indirect	low	Indirectness[1]; inaccuracy[2]
Ustekinumab vs Iberdomide	Indirect	low	Indirectness[1]; inaccuracy[2]
Ustekinumab vs Atacicept	Indirect	low	Indirectness[1]; inaccuracy[2]
Ustekinumab vs PF04236921	Indirect	low	Indirectness[1]; inaccuracy[2]
Ustekinumab vs Dapirolizumabpegol	Indirect	low	Indirectness[1]; inaccuracy[2]
Ustekinumab vs Evobrutinib	Indirect	low	Indirectness[1]; inaccuracy[2]
Ustekinumab vs Baricitinib	Indirect	Medium	Indirectness[1]
Ustekinumab vs Epratuzumab	Indirect	Medium	Indirectness[1]
Ustekinumab vs Placebo	Direct	high	none

ABBV-599highdose vs Deucravacitinib	Indirect	low	Indirectness[1]; inaccuracy[2]
ABBV-599highdose vs Anifrolumab	Indirect	low	Indirectness[1]; inaccuracy[2]
ABBV-599highdose vs Upadacitinib	Indirect	low	Indirectness[1]; inaccuracy[2]
ABBV-599highdose vs Sifalimumab	Indirect	low	Indirectness[1]; inaccuracy[2]
ABBV-599highdose vs Iberdomide	Indirect	low	Indirectness[1]; inaccuracy[2]
ABBV-599highdose vs Atacicept	Indirect	low	Indirectness[1]; inaccuracy[2]
ABBV-599highdose vs PF04236921	Indirect	low	Indirectness[1]; inaccuracy[2]
ABBV-599highdose vs Dapirolizumabpegol	Indirect	low	Indirectness[1]; inaccuracy[2]
ABBV-599highdose vs Evobrutinib	Indirect	low	Indirectness[1]; inaccuracy[2]
ABBV-599highdose vs Baricitinib	Indirect	low	Indirectness[1]; inaccuracy[2]
ABBV-599highdose vs Epratuzumab	Indirect	low	Indirectness[1]; inaccuracy[2]
ABBV-599highdose vs Placebo	Direct	high	none
Deucravacitinib vs Anifrolumab	Indirect	low	Indirectness[1]; inaccuracy[2]
Deucravacitinib vs Upadacitinib	Indirect	low	Indirectness[1]; inaccuracy[2]
Deucravacitinib vs Sifalimumab	Indirect	low	Indirectness[1]; inaccuracy[2]
Deucravacitinib vs Iberdomide	Indirect	low	Indirectness[1]; inaccuracy[2]
Deucravacitinib vs Atacicept	Indirect	low	Indirectness[1]; inaccuracy[2]
Deucravacitinib vs PF04236921	Indirect	low	Indirectness[1]; inaccuracy[2]
Deucravacitinib vs Dapirolizumabpegol	Indirect	low	Indirectness[1]; inaccuracy[2]
Deucravacitinib vs Evobrutinib	Indirect	low	Indirectness[1]; inaccuracy[2]
Deucravacitinib vs Baricitinib	Indirect	low	Indirectness[1]; inaccuracy[2]
Deucravacitinib vs Epratuzumab	Indirect	low	Indirectness[1]; inaccuracy[2]
Deucravacitinib vs Placebo	Direct	high	none
Anifrolumab vs Upadacitinib	Indirect	low	Indirectness[1]; inaccuracy[2]
Anifrolumab vs Sifalimumab	Indirect	low	Indirectness[1]; inaccuracy[2]
Anifrolumab vs Iberdomide	Indirect	low	Indirectness[1]; inaccuracy[2]
Anifrolumab vs Atacicept	Indirect	low	Indirectness[1]; inaccuracy[2]
Anifrolumab vs PF04236921	Indirect	low	Indirectness[1]; inaccuracy[2]
Anifrolumab vs Dapirolizumabpegol	Indirect	low	Indirectness[1]; inaccuracy[2]
Anifrolumab vs Evobrutinib	Indirect	low	Indirectness[1]; inaccuracy[2]
Anifrolumab vs Baricitinib	Indirect	low	Indirectness[1]; inaccuracy[2]
Anifrolumab vs Epratuzumab	Indirect	Medium	Indirectness[1]
Anifrolumab vs Placebo	Direct	high	none
Upadacitinib vs Sifalimumab	Indirect	low	Indirectness[1]; inaccuracy[2]
Upadacitinib vs Iberdomide	Indirect	low	Indirectness[1]; inaccuracy[2]
Upadacitinib vs Atacicept	Indirect	low	Indirectness[1]; inaccuracy[2]
Upadacitinib vs PF04236921	Indirect	low	Indirectness[1]; inaccuracy[2]
Upadacitinib vs Dapirolizumabpegol	Indirect	low	Indirectness[1]; inaccuracy[2]
Upadacitinib vs Evobrutinib	Indirect	low	Indirectness[1]; inaccuracy[2]
Upadacitinib vs Baricitinib	Indirect	low	Indirectness[1]; inaccuracy[2]
Upadacitinib vs Epratuzumab	Indirect	low	Indirectness[1]; inaccuracy[2]
Upadacitinib vs Placebo	Direct	Medium	inaccuracy[2]
Sifalimumab vs Iberdomide	Indirect	low	Indirectness[1]; inaccuracy[2]

Sifalimumab vs Atacicept	Indirect	low	Indirectness[1]; inaccuracy[2]
Sifalimumab vs PF04236921	Indirect	low	Indirectness[1]; inaccuracy[2]
Sifalimumab vs Dapirolizumabpegol	Indirect	low	Indirectness[1]; inaccuracy[2]
Sifalimumab vs Evobrutinib	Indirect	low	Indirectness[1]; inaccuracy[2]
Sifalimumab vs Baricitinib	Indirect	low	Indirectness[1]; inaccuracy[2]
Sifalimumab vs Epratuzumab	Indirect	low	Indirectness[1]; inaccuracy[2]
Sifalimumab vs Placebo	Direct	high	none
Iberdomide vs Atacicept	Indirect	low	Indirectness[1]; inaccuracy[2]
Iberdomide vs PF04236921	Indirect	low	Indirectness[1]; inaccuracy[2]
Iberdomide vs Dapirolizumabpegol	Indirect	low	Indirectness[1]; inaccuracy[2]
Iberdomide vs Evobrutinib	Indirect	low	Indirectness[1]; inaccuracy[2]
Iberdomide vs Baricitinib	Indirect	low	Indirectness[1]; inaccuracy[2]
Iberdomide vs Epratuzumab	Indirect	low	Indirectness[1]; inaccuracy[2]
Iberdomide vs Placebo	Direct	Medium	inaccuracy[2]
Atacicept vs PF04236921	Indirect	low	Indirectness[1]; inaccuracy[2]
Atacicept vs Dapirolizumabpegol	Indirect	low	Indirectness[1]; inaccuracy[2]
Atacicept vs Evobrutinib	Indirect	low	Indirectness[1]; inaccuracy[2]
Atacicept vs Baricitinib	Indirect	low	Indirectness[1]; inaccuracy[2]
Atacicept vs Epratuzumab	Indirect	low	Indirectness[1]; inaccuracy[2]
Atacicept vs Placebo	Direct	Medium	inaccuracy[2]
Baricitinib vs Placebo	Direct	Medium	inaccuracy[2]
Epratuzumab vs Placebo	Direct	Medium	inaccuracy[2]
PF04236921 vs Dapirolizumabpegol	Indirect	low	Indirectness[1]; inaccuracy[2]
PF04236921 vs Evobrutinib	Indirect	low	Indirectness[1]; inaccuracy[2]
PF04236921 vs Baricitinib	Indirect	low	Indirectness[1]; inaccuracy[2]
PF04236921 vs Epratuzumab	Indirect	low	Indirectness[1]; inaccuracy[2]
PF04236921 vs Placebo	Direct	Medium	inaccuracy[2]
Dapirolizumabpegol vs Evobrutinib	Indirect	low	Indirectness[1]; inaccuracy[2]
Dapirolizumabpegol vs Baricitinib	Indirect	low	Indirectness[1]; inaccuracy[2]
Dapirolizumabpegol vs Epratuzumab	Indirect	low	Indirectness[1]; inaccuracy[2]
Dapirolizumabpegol vs Placebo	Direct	Medium	inaccuracy[2]
Evobrutinib vs Baricitinib	Indirect	low	Indirectness[1]; inaccuracy[2]
Evobrutinib vs Epratuzumab	Indirect	low	Indirectness[1]; inaccuracy[2]
Evobrutinib vs Placebo	Direct	Medium	inaccuracy[2]
Baricitinib vs Epratuzumab	Indirect	low	Indirectness[1]; inaccuracy[2]

notes:1: No head-to-head RCTs, evidence derived from indirect comparisons via placebo; 2: 95% CI crossing the null line.

Table S29 GRADE Assessment for BICLA.

Treatment regimen	Nature of evidence	Quality grade	Downgrading factor
Upadacitinib vs ABBV-599high-dose	Indirect	low	Indirectness[1]; inaccuracy[2]
Upadacitinib vs PF04236921	Indirect	low	Indirectness[1]; inaccuracy[2]
Upadacitinib vs Anifrolumab	Indirect	low	Indirectness[1]; inaccuracy[2]

Upadacitinib vs Deucravacitinib	Indirect	low	Indirectness[1]; inaccuracy[2]
Upadacitinib vs Dapirolizumabpegol	Indirect	low	Indirectness[1]; inaccuracy[2]
Upadacitinib vs E6742	Indirect	low	Indirectness[1]; inaccuracy[2]
Upadacitinib vs Baricitinib	Indirect	Medium	Indirectness[1]
Upadacitinib vs Epratuzumab	Indirect	Medium	Indirectness[1]
Upadacitinib vs Ustekinumab	Indirect	Medium	Indirectness[1]
Upadacitinib vs Placebo	Direct	high	none
ABBV-599high-dose vs PF04236921	Indirect	low	Indirectness[1]; inaccuracy[2]
ABBV-599high-dose vs Anifrolumab	Indirect	low	Indirectness[1]; inaccuracy[2]
ABBV-599high-dose vs Deucravacitinib	Indirect	low	Indirectness[1]; inaccuracy[2]
ABBV-599high-dose vs Dapirolizumabpegol	Indirect	low	Indirectness[1]; inaccuracy[2]
ABBV-599high-dose vs E6742	Indirect	low	Indirectness[1]; inaccuracy[2]
ABBV-599high-dose vs Baricitinib	Indirect	Medium	Indirectness[1]
ABBV-599high-dose vs Epratuzumab	Indirect	Medium	Indirectness[1]
ABBV-599high-dose vs Ustekinumab	Indirect	low	Indirectness[1]; inaccuracy[2]
ABBV-599high-dose vs Placebo	Direct	high	none
PF04236921 vs Anifrolumab	Indirect	low	Indirectness[1]; inaccuracy[2]
PF04236921 vs Deucravacitinib	Indirect	low	Indirectness[1]; inaccuracy[2]
PF04236921 vs Dapirolizumabpegol	Indirect	low	Indirectness[1]; inaccuracy[2]
PF04236921 vs E6742	Indirect	low	Indirectness[1]; inaccuracy[2]
PF04236921 vs Baricitinib	Indirect	low	Indirectness[1]; inaccuracy[2]
PF04236921 vs Epratuzumab	Indirect	low	Indirectness[1]; inaccuracy[2]
PF04236921 vs Ustekinumab	Indirect	low	Indirectness[1]; inaccuracy[2]
PF04236921 vs Placebo	Direct	high	none
Anifrolumab vs Deucravacitinib	Indirect	low	Indirectness[1]; inaccuracy[2]
Anifrolumab vs Dapirolizumabpegol	Indirect	low	Indirectness[1]; inaccuracy[2]
Anifrolumab vs E6742	Indirect	low	Indirectness[1]; inaccuracy[2]
Anifrolumab vs Baricitinib	Indirect	Medium	Indirectness[1]
Anifrolumab vs Epratuzumab	Indirect	Medium	Indirectness[1]
Anifrolumab vs Ustekinumab	Indirect	low	Indirectness[1];inaccuracy2
Anifrolumab vs Placebo	Direct	high	none
Deucravacitinib vs Dapirolizumabpegol	Indirect	low	Indirectness[1]; inaccuracy[2]
Deucravacitinib vs E6742	Indirect	low	Indirectness[1]; inaccuracy[2]
Deucravacitinib vs Baricitinib	Indirect	low	Indirectness[1]; inaccuracy[2]
Deucravacitinib vs Epratuzumab	Indirect	low	Indirectness[1]; inaccuracy[2]
Deucravacitinib vs Ustekinumab	Indirect	low	Indirectness[1]; inaccuracy[2]
Deucravacitinib vs Placebo	Direct	high	none
Dapirolizumabpegol vs E6742	Indirect	low	Indirectness[1]; inaccuracy[2]
Dapirolizumabpegol vs Baricitinib	Indirect	low	Indirectness[1]; inaccuracy[2]
Dapirolizumabpegol vs Epratuzumab	Indirect	low	Indirectness[1]; inaccuracy[2]
Dapirolizumabpegol vs Ustekinumab	Indirect	low	Indirectness[1]; inaccuracy[2]
Dapirolizumabpegol vs Placebo	Direct	Medium	inaccuracy[2]
E6742 vs Baricitinib	Indirect	low	Indirectness[1]; inaccuracy[2]
E6742 vs Epratuzumab	Indirect	low	Indirectness[1]; inaccuracy[2]

E6742 vs Ustekinumab	Indirect	low	Indirectness[1]; inaccuracy[2]
E6742 vs Placebo	Direct	Medium	inaccuracy[2]
Baricitinib vs Epratuzumab	Indirect	low	Indirectness[1]; inaccuracy[2]
Baricitinib vs Ustekinumab	Indirect	low	Indirectness[1]; inaccuracy[2]
Baricitinib vs Placebo	Direct	Medium	inaccuracy[2]
Epratuzumab vs Ustekinumab	Indirect	low	Indirectness[1]; inaccuracy[2]
Epratuzumab vs Placebo	Direct	Medium	inaccuracy[2]
Ustekinumab vs Placebo	Direct	Medium	inaccuracy[2]

notes:[1]: No head-to-head RCTs, evidence derived from indirect comparisons via placebo; 2: 95% CI crossing the null line.

Table S30 GRADE Assessment for CLASI-50.

Treatment regimen	Nature of evidence	Quality grade	Downgrading factor
Deucravacitinib vs Anifrolumab	Indirect	low	Indirectness[1]; inaccuracy[2]
Deucravacitinib vs Upadacitinib	Indirect	low	Indirectness[1]; inaccuracy[2]
Deucravacitinib vs Sifalimumab	Indirect	low	Indirectness[1]; inaccuracy[2]
Deucravacitinib vs ABBV-599high-dose	Indirect	low	Indirectness[1]; inaccuracy[2]
Deucravacitinib vs Iberdomide	Indirect	Medium	Indirectness[1]
Deucravacitinib vs Placebo	Direct	high	none
Deucravacitinib vs Baricitinib	Indirect	Medium	Indirectness[1]
Anifrolumab vs Upadacitinib	Indirect	low	Indirectness[1]; inaccuracy[2]
Anifrolumab vs Sifalimumab	Indirect	low	Indirectness[1]; inaccuracy[2]
Anifrolumab vs ABBV-599high-dose	Indirect	low	Indirectness[1]; inaccuracy[2]
Anifrolumab vs Iberdomide	Indirect	low	Indirectness[1]; inaccuracy[2]
Anifrolumab vs Placebo	Direct	high	none
Anifrolumab vs Baricitinib	Indirect	Medium	Indirectness[1]
Upadacitinib vs Sifalimumab	Indirect	low	Indirectness[1]; inaccuracy[2]
Upadacitinib vs ABBV-599high-dose	Indirect	low	Indirectness[1]; inaccuracy[2]
Upadacitinib vs Iberdomide	Indirect	low	Indirectness[1]; inaccuracy[2]
Upadacitinib vs Placebo	Direct	Medium	inaccuracy[2]
Upadacitinib vs Baricitinib	Indirect	low	Indirectness[1]; inaccuracy[2]
Sifalimumab vs ABBV-599high-dose	Indirect	low	Indirectness[1]; inaccuracy[2]
Sifalimumab vs Iberdomide	Indirect	low	Indirectness[1]; inaccuracy[2]
Sifalimumab vs Placebo	Direct	high	none
Sifalimumab vs Baricitinib	Indirect	low	Indirectness[1]; inaccuracy[2]
ABBV-599high-dose vs Iberdomide	Indirect	low	Indirectness[1]; inaccuracy[2]
ABBV-599high-dose vs Placebo	Direct	Medium	inaccuracy[2]
ABBV-599high-dose vs Baricitinib	Indirect	low	Indirectness[1]; inaccuracy[2]
Iberdomide vs Placebo	Direct	Medium	inaccuracy2
Iberdomide vs Baricitinib	Indirect	low	Indirectness[1]; inaccuracy[2]
Placebo vs Baricitinib	Direct	Medium	inaccuracy[2]

notes:1: No head-to-head RCTs, evidence derived from indirect comparisons via placebo; 2: 95% CI crossing the null line.

Table S31 GRADE Assessment for LLDAS.

Treatment regimen	Nature of evidence	Quality grade	Downgrading factor
Upadacitinib vs Deucravacitinib	Indirect	low	Indirectness[1]; inaccuracy[2]
Upadacitinib vs ABBV-599high-dose	Indirect	low	Indirectness[1]; inaccuracy[2]
Upadacitinib vs Baricitinib	Indirect	Medium	Indirectness[1]
Upadacitinib vs Placebo	Direct	high	none
Deucravacitinib vs ABBV-599high-dose	Indirect	low	Indirectness[1]; inaccuracy[2]
Deucravacitinib vs Baricitinib	Indirect	Medium	Indirectness[1]
Deucravacitinib vs Placebo	Direct	high	none
ABBV-599high-dose vs Baricitinib	Indirect	low	Indirectness[1]; inaccuracy[2]
ABBV-599high-dose vs Placebo	Direct	high	none
Baricitinib vs Placebo	Direct	Medium	inaccuracy[2]

notes:1: No head-to-head RCTs, evidence derived from indirect comparisons via placebo; 2: 95% CI crossing the null line.

Table S32 GRADE Assessment for AE.

Treatment regimen	Nature of evidence	Quality grade	Downgrading factor
Cenerimod vs Belimumab	Indirect	very low	Indirectness[1];inaccuracy[2];risk of bias[3]
Cenerimod vs Epratuzumab	Indirect	very low	Indirectness[1];inaccuracy[2];risk of bias[3]
Belimumab vs Anifrolumab	Indirect	low	Indirectness[1];risk of bias[3]
Belimumab vs Ustekinumab	Indirect	very low	Indirectness[1];inaccuracy[2];risk of bias[3]
Belimumab vs Iberdomide	Indirect	low	Indirectness[1];risk of bias[3]
Belimumab vs IL-2	Indirect	very low	Indirectness[1];inaccuracy[2];risk of bias[3]
Belimumab vs ILT-101	Indirect	low	Indirectness[1];risk of bias[3]
Belimumab vs Telitacicept	Indirect	low	Indirectness[1];risk of bias[3]
Epratuzumab vs Placebo	Direct	low	Indirectness[1];risk of bias[3]
Epratuzumab vs Deucravacitinib	Indirect	very low	Indirectness[1];inaccuracy[2];risk of bias[3]
Epratuzumab vs Sifalimumab	Indirect	very low	Indirectness[1];inaccuracy[2];risk of bias[3]
Epratuzumab vs Anifrolumab	Indirect	very low	Indirectness[1];inaccuracy[2];risk of bias[3]
Epratuzumab vs Ustekinumab	Indirect	very low	Indirectness[1];inaccuracy[2];risk of bias[3]
Epratuzumab vs Iberdomide	Indirect	very low	Indirectness[1];inaccuracy[2];risk of bias[3]
Epratuzumab vs IL-2	Indirect	very low	Indirectness[1];inaccuracy[2];risk of bias[3]
Epratuzumab vs ILT-101	Indirect	very low	Indirectness[1];inaccuracy[2];risk of bias[3]
Epratuzumab vs Telitacicept	Indirect	very low	Indirectness[1];inaccuracy[2];risk of bias[3]
Placebo vs Deucravacitinib	Direct	low	Indirectness[1];risk of bias[3]
Placebo vs Sifalimumab	Direct	low	Indirectness[1];risk of bias[3]
Placebo vs Anifrolumab	Direct	Medium	risk of bias3
Placebo vs Ustekinumab	Direct	low	inaccuracy[2];risk of bias[3]
Placebo vs Iberdomide	Direct	Medium	inaccuracy[2];risk of bias[3]
Placebo vs IL-2	Direct	low	inaccuracy[2];risk of bias[3]
Placebo vs ILT-101	Direct	low	inaccuracy[2];risk of bias[3]
Placebo vs Telitacicept	Direct	Medium	risk of bias[3]
Deucravacitinib vs Sifalimumab	Indirect	very low	Indirectness[1];inaccuracy[2];risk of bias[3]

Deucravacitinib vs Anifrolumab	Indirect	very low	Indirectness[1];inaccuracy[2];risk of bias[3]
Deucravacitinib vs Ustekinumab	Indirect	very low	Indirectness[1];inaccuracy[2];risk of bias[3]
Deucravacitinib vs Iberdomide	Indirect	very low	Indirectness[1];inaccuracy[2];risk of bias[3]
Deucravacitinib vs IL-2	Indirect	very low	Indirectness[1];inaccuracy[2];risk of bias[3]
Deucravacitinib vs ILT-101	Indirect	very low	Indirectness[1];inaccuracy[2];risk of bias[3]
Deucravacitinib vs Telitacicept	Indirect	very low	Indirectness[1];inaccuracy[2];risk of bias[3]
Sifalimumab vs Anifrolumab	Indirect	very low	Indirectness[1];inaccuracy[2];risk of bias[3]
Sifalimumab vs Ustekinumab	Indirect	very low	Indirectness[1];inaccuracy[2];risk of bias[3]
Sifalimumab vs Iberdomide	Indirect	very low	Indirectness[1];inaccuracy[2];risk of bias[3]
Sifalimumab vs IL-2	Indirect	very low	Indirectness[1];inaccuracy[2];risk of bias[3]
Sifalimumab vs ILT-101	Indirect	very low	Indirectness[1];inaccuracy[2];risk of bias[3]
Sifalimumab vs Telitacicept	Indirect	very low	Indirectness[1];inaccuracy[2];risk of bias[3]
Anifrolumab vs Ustekinumab	Indirect	very low	Indirectness[1];inaccuracy[2];risk of bias[3]
Anifrolumab vs Iberdomide	Indirect	very low	Indirectness[1];inaccuracy[2];risk of bias[3]
Anifrolumab vs IL-2	Indirect	very low	Indirectness[1];inaccuracy[2];risk of bias[3]
Anifrolumab vs ILT-101	Indirect	very low	Indirectness[1];inaccuracy[2];risk of bias[3]
Anifrolumab vs Telitacicept	Indirect	very low	Indirectness[1];inaccuracy[2];risk of bias[3]
Ustekinumab vs Iberdomide	Indirect	very low	Indirectness[1];inaccuracy[2];risk of bias[3]
Ustekinumab vs IL-2	Indirect	very low	Indirectness[1];inaccuracy[2];risk of bias[3]
Ustekinumab vs ILT-101	Indirect	very low	Indirectness[1];inaccuracy[2];risk of bias[3]
Ustekinumab vs Telitacicept	Indirect	very low	Indirectness[1];inaccuracy[2];risk of bias[3]
Iberdomide vs IL-2	Indirect	very low	Indirectness[1];inaccuracy[2];risk of bias[3]
Iberdomide vs ILT-101	Indirect	very low	Indirectness[1];inaccuracy[2];risk of bias[3]
Iberdomide vs Telitacicept	Indirect	very low	Indirectness[1];inaccuracy[2];risk of bias[3]
IL-2 vs ILT-101	Indirect	very low	Indirectness[1];inaccuracy[2];risk of bias[3]
IL-2 vs Telitacicept	Indirect	very low	Indirectness[1];inaccuracy[2];risk of bias[3]
ILT-101 vs Telitacicept	Indirect	very low	Indirectness[1];inaccuracy[2];risk of bias[3]
Cenerimod vs Placebo	Direct	low	inaccuracy[2];risk of bias[3]
Cenerimod vs Deucravacitinib	Indirect	very low	Indirectness[1];inaccuracy[2];risk of bias[3]
Cenerimod vs Sifalimumab	Indirect	very low	Indirectness[1];inaccuracy[2];risk of bias[3]
Cenerimod vs Anifrolumab	Indirect	low	Indirectness[1];risk of bias[3]
Cenerimod vs Ustekinumab	Indirect	very low	Indirectness[1];inaccuracy[2];risk of bias[3]
Cenerimod vs Iberdomide	Indirect	low	Indirectness[1];risk of bias3
Cenerimod vs IL-2	Indirect	very low	Indirectness[1];inaccuracy[2];risk of bias[3]
Cenerimod vs ILT-101	Indirect	low	Indirectness[1];risk of bias[3]
Cenerimod vs Telitacicept	Indirect	low	Indirectness[1];risk of bias[3]
Belimumab vs Epratuzumab	Indirect	very low	Indirectness[1];inaccuracy[2];risk of bias[3]
Belimumab vs Placebo	Direct	low	inaccuracy[2];risk of bias[3]
Belimumab vs Deucravacitinib	Indirect	very low	Indirectness[1];inaccuracy[2];risk of bias[3]
Belimumab vs Sifalimumab	Indirect	very low	Indirectness[1];inaccuracy[2];risk of bias[3]

notes:1: No head-to-head RCTs, evidence derived from indirect comparisons via placebo; 2: 95% CI crossing the null line; 3: risk of bias.

Table S33 GRADE Assessment for SAE.

Treatment regimen	Nature of	Quality	Downgrading factor

	evidence	grade	
Cenerimod vs IL-2	Indirect	very low	Indirectness[1];inaccuracy[2];risk of bias[3]
Cenerimod vs deucravacitinib	Indirect	very low	Indirectness[1];inaccuracy[2];risk of bias[3]
Cenerimod vs Anifrolumab	Indirect	very low	Indirectness[1];inaccuracy[2];risk of bias[3]
Cenerimod vs Belimumab	Indirect	very low	Indirectness[1];inaccuracy[2];risk of bias[3]
Cenerimod vs Iberdomide	Indirect	very low	Indirectness[1];inaccuracy[2];risk of bias[3]
Cenerimod vs Telitacicept	Indirect	very low	Indirectness[1];inaccuracy[2];risk of bias[3]
Cenerimod vs Ustekinumab	Indirect	very low	Indirectness[1];inaccuracy[2];risk of bias[3]
Cenerimod vs Epratuzumab	Indirect	very low	Indirectness[1];inaccuracy[2];risk of bias[3]
Cenerimod vs Tabalumab	Indirect	very low	Indirectness[1];inaccuracy[2];risk of bias[3]
Cenerimod vs Placebo	Direct	low	inaccuracy[2];risk of bias[3]
Cenerimod vs Sifalimumab	Indirect	very low	Indirectness[1];inaccuracy[2];risk of bias[3]
Cenerimod vs ILT-101	Indirect	very low	Indirectness[1];inaccuracy[2];risk of bias[3]
Cenerimod vs Baricitinib	Indirect	very low	Indirectness[1];inaccuracy[2];risk of bias[3]
IL-2 vs deucravacitinib	Indirect	very low	Indirectness[1];inaccuracy[2];risk of bias[3]
IL-2 vs Anifrolumab	Indirect	very low	Indirectness[1];inaccuracy[2];risk of bias[3]
IL-2 vs Belimumab	Indirect	very low	Indirectness[1];inaccuracy[2];risk of bias[3]
IL-2 vs Iberdomide	Indirect	very low	Indirectness[1];inaccuracy[2];risk of bias[3]
IL-2 vs Telitacicept	Indirect	very low	Indirectness[1];inaccuracy[2];risk of bias[3]
IL-2 vs Ustekinumab	Indirect	very low	Indirectness[1];inaccuracy[2];risk of bias[3]
IL-2 vs Epratuzumab	Indirect	very low	Indirectness[1];inaccuracy[2];risk of bias[3]
IL-2 vs Tabalumab	Indirect	very low	Indirectness[1];inaccuracy[2];risk of bias[3]
IL-2 vs Placebo	Direct	low	inaccuracy[2];risk of bias[3]
IL-2 vs Sifalimumab	Indirect	very low	Indirectness[1];inaccuracy[2];risk of bias[3]
IL-2 vs ILT-101	Indirect	very low	Indirectness[1];inaccuracy[2];risk of bias[3]
IL-2 vs Baricitinib	Indirect	very low	Indirectness[1];inaccuracy[2];risk of bias[3]
deucravacitinib vs Anifrolumab	Indirect	very low	Indirectness[1];inaccuracy[2];risk of bias[3]
deucravacitinib vs Belimumab	Indirect	very low	Indirectness[1];inaccuracy[2];risk of bias[3]
deucravacitinib vs Iberdomide	Indirect	very low	Indirectness[1];inaccuracy[2];risk of bias[3]
deucravacitinib vs Telitacicept	Indirect	very low	Indirectness[1];inaccuracy[2];risk of bias[3]
deucravacitinib vs Ustekinumab	Indirect	very low	Indirectness[1];inaccuracy[2];risk of bias[3]
deucravacitinib vs Epratuzumab	Indirect	very low	Indirectness[1];inaccuracy[2];risk of bias[3]
deucravacitinib vs Tabalumab	Indirect	very low	Indirectness[1];inaccuracy[2];risk of bias[3]
deucravacitinib vs Placebo	Direct	low	inaccuracy[2];risk of bias[3]
deucravacitinib vs Sifalimumab	Indirect	very low	Indirectness[1];inaccuracy[2];risk of bias[3]
deucravacitinib vs ILT-101	Indirect	very low	Indirectness[1];inaccuracy[2];risk of bias[3]
deucravacitinib vs Baricitinib	Indirect	very low	Indirectness[1];inaccuracy[2];risk of bias[3]
Anifrolumab vs Belimumab	Indirect	very low	Indirectness[1];inaccuracy[2];risk of bias[3]
Anifrolumab vs Iberdomide	Indirect	very low	Indirectness[1];inaccuracy[2];risk of bias[3]
Anifrolumab vs Telitacicept	Indirect	very low	Indirectness[1];inaccuracy[2];risk of bias[3]
Anifrolumab vs Ustekinumab	Indirect	very low	Indirectness[1];inaccuracy[2];risk of bias[3]
Anifrolumab vs Epratuzumab	Indirect	very low	Indirectness[1];inaccuracy[2];risk of bias[3]
Anifrolumab vs Tabalumab	Indirect	very low	Indirectness[1];inaccuracy[2];risk of bias[3]
Anifrolumab vs Placebo	Direct	low	inaccuracy[2];risk of bias[3]

Anifrolumab vs Sifalimumab	Indirect	very low	Indirectness[1];inaccuracy[2];risk of bias[3]
Anifrolumab vs ILT-101	Indirect	very low	Indirectness[1];inaccuracy[2];risk of bias[3]
Anifrolumab vs Baricitinib	Indirect	low	inaccuracy[2];risk of bias[3]
Belimumab vs Iberdomide	Indirect	very low	Indirectness[1];inaccuracy[2];risk of bias[3]
Belimumab vs Telitacicept	Indirect	very low	Indirectness[1];inaccuracy[2];risk of bias[3]
Belimumab vs Ustekinumab	Indirect	very low	Indirectness[1];inaccuracy[2];risk of bias[3]
Belimumab vs Epratuzumab	Indirect	very low	Indirectness[1];inaccuracy[2];risk of bias[3]
Belimumab vs Tabalumab	Indirect	very low	Indirectness[1];inaccuracy[2];risk of bias[3]
Belimumab vs Placebo	Direct	Medium	risk of bias[3]
Belimumab vs Sifalimumab	Indirect	very low	Indirectness[1];inaccuracy[2];risk of bias[3]
Belimumab vs ILT-101	Indirect	very low	Indirectness[1];inaccuracy[2];risk of bias[3]
Belimumab vs Baricitinib	Indirect	low	inaccuracy[2];risk of bias[3]
Iberdomide vs Telitacicept	Indirect	very low	Indirectness[1];inaccuracy[2];risk of bias[3]
Iberdomide vs Ustekinumab	Indirect	very low	Indirectness[1];inaccuracy[2];risk of bias[3]
Iberdomide vs Epratuzumab	Indirect	very low	Indirectness[1];inaccuracy[2];risk of bias[3]
Iberdomide vs Tabalumab	Indirect	very low	Indirectness[1];inaccuracy[2];risk of bias[3]
Iberdomide vs Placebo	Direct	low	inaccuracy[2];risk of bias[3]
Iberdomide vs Sifalimumab	Indirect	very low	Indirectness[1];inaccuracy[2];risk of bias[3]
Iberdomide vs ILT-101	Indirect	very low	Indirectness[1];inaccuracy[2];risk of bias[3]
Iberdomide vs Baricitinib	Indirect	very low	Indirectness[1];inaccuracy[2];risk of bias[3]
Telitacicept vs Ustekinumab	Indirect	very low	Indirectness[1];inaccuracy[2];risk of bias[3]
Telitacicept vs Epratuzumab	Indirect	very low	Indirectness[1];inaccuracy[2];risk of bias[3]
Telitacicept vs Tabalumab	Indirect	very low	Indirectness[1];inaccuracy[2];risk of bias[3]
Telitacicept vs Placebo	Direct	low	inaccuracy[2];risk of bias[3]
Telitacicept vs Sifalimumab	Indirect	very low	Indirectness[1];inaccuracy[2];risk of bias[3]
Telitacicept vs ILT-101	Indirect	very low	Indirectness[1];inaccuracy[2];risk of bias[3]
Telitacicept vs Baricitinib	Indirect	very low	Indirectness[1];inaccuracy[2];risk of bias[3]
Ustekinumab vs Epratuzumab	Indirect	very low	Indirectness[1];inaccuracy[2];risk of bias[3]
Ustekinumab vs Tabalumab	Indirect	very low	Indirectness[1];inaccuracy[2];risk of bias[3]
Ustekinumab vs Placebo	Direct	low	inaccuracy[2];risk of bias[3]
Ustekinumab vs Sifalimumab	Indirect	very low	Indirectness[1];inaccuracy[2];risk of bias[3]
Ustekinumab vs ILT-101	Indirect	very low	Indirectness[1];inaccuracy[2];risk of bias[3]
Ustekinumab vs Baricitinib	Indirect	very low	Indirectness[1];inaccuracy[2];risk of bias[3]
Epratuzumab vs Tabalumab	Indirect	very low	Indirectness[1];inaccuracy[2];risk of bias[3]
Epratuzumab vs Placebo	Direct	low	inaccuracy[2];risk of bias[3]
Epratuzumab vs Sifalimumab	Indirect	very low	Indirectness[1];inaccuracy[2];risk of bias[3]
Epratuzumab vs ILT-101	Indirect	very low	Indirectness[1];inaccuracy[2];risk of bias[3]
Epratuzumab vs Baricitinib	Indirect	very low	Indirectness[1];inaccuracy[2];risk of bias[3]
Tabalumab vs Placebo	Direct	low	inaccuracy[2];risk of bias[3]
Tabalumab vs Sifalimumab	Indirect	very low	Indirectness[1];inaccuracy[2];risk of bias[3]
Tabalumab vs ILT-101	Indirect	very low	Indirectness[1];inaccuracy[2];risk of bias[3]
Tabalumab vs Baricitinib	Indirect	very low	Indirectness[1];inaccuracy[2];risk of bias[3]
Placebo vs Sifalimumab	Direct	low	inaccuracy[2];risk of bias[3]
Placebo vs ILT-101	Direct	low	inaccuracy[2];risk of bias[3]

Placebo vs Baricitinib	Direct	low	inaccuracy[2];risk of bias[3]
Sifalimumab vs ILT-101	Indirect	very low	Indirectness[1];inaccuracy[2];risk of bias[3]
Sifalimumab vs Baricitinib	Indirect	very low	Indirectness[1];inaccuracy[2];risk of bias[3]
ILT-101 vs Baricitinib	Indirect	very low	Indirectness[1];inaccuracy[2];risk of bias[3]

notes:1: No head-to-head RCTs, evidence derived from indirect comparisons via placebo; 2: 95% CI crossing the null line; 3: risk of bias.

Table S34 GRADE Assessment for Infection related AEs.

Treatment regimen	Nature of evidence	Quality grade	Downgrading factor
IL-2 vs Epratuzumab	Indirect	low	Indirectness[1];inaccuracy[2]
IL-2 vs Ustekinumab	Indirect	low	Indirectness[1];inaccuracy[2]
IL-2 vs Belimumab	Indirect	low	Indirectness[1];inaccuracy[2]
IL-2 vs Tabalumab	Indirect	low	Indirectness[1];inaccuracy[2]
IL-2 vs Blisibimod	Indirect	low	Indirectness[1];inaccuracy[2]
IL-2 vs Placebo	Direct	Medium	inaccuracy[2]
IL-2 vs E6742	Indirect	low	Indirectness[1];inaccuracy[2]
IL-2 vs deucravacitinib	Indirect	low	Indirectness[1];inaccuracy[2]
IL-2 vs Baricitinib	Indirect	low	Indirectness[1];inaccuracy[2]
IL-2 vs Telitacicept	Indirect	low	Indirectness[1];inaccuracy[2]
Epratuzumab vs Ustekinumab	Indirect	low	Indirectness[1];inaccuracy[2]
Epratuzumab vs Belimumab	Indirect	low	Indirectness[1];inaccuracy[2]
Epratuzumab vs Tabalumab	Indirect	low	Indirectness[1];inaccuracy[2]
Epratuzumab vs Blisibimod	Indirect	low	Indirectness[1];inaccuracy[2]
Epratuzumab vs Placebo	Direct	Medium	inaccuracy[2]
Epratuzumab vs E6742	Indirect	low	Indirectness[1];inaccuracy[2]
Epratuzumab vs deucravacitinib	Indirect	low	Indirectness[1];inaccuracy[2]
Epratuzumab vs Baricitinib	Indirect	low	Indirectness[1];inaccuracy[2]
Epratuzumab vs Telitacicept	Indirect	low	Indirectness[1];inaccuracy[2]
Ustekinumab vs Belimumab	Indirect	low	Indirectness[1];inaccuracy[2]
Ustekinumab vs Tabalumab	Indirect	low	Indirectness[1];inaccuracy[2]
Ustekinumab vs Blisibimod	Indirect	low	Indirectness[1];inaccuracy[2]
Ustekinumab vs Placebo	Direct	Medium	inaccuracy[2]
Ustekinumab vs E6742	Indirect	low	Indirectness[1];inaccuracy[2]
Ustekinumab vs deucravacitinib	Indirect	low	Indirectness[1];inaccuracy[2]
Ustekinumab vs Baricitinib	Indirect	low	Indirectness[1];inaccuracy[2]
Ustekinumab vs Telitacicept	Indirect	low	Indirectness[1];inaccuracy[2]
Belimumab vs Tabalumab	Indirect	low	Indirectness[1];inaccuracy[2]
Belimumab vs Blisibimod	Indirect	low	Indirectness[1];inaccuracy[2]
Belimumab vs Placebo	Direct	Medium	inaccuracy[2]
Belimumab vs E6742	Indirect	low	Indirectness[1];inaccuracy[2]
Belimumab vs deucravacitinib	Indirect	low	Indirectness[1];inaccuracy[2]
Belimumab vs Baricitinib	Indirect	low	Indirectness[1];inaccuracy[2]
Belimumab vs Telitacicept	Indirect	low	Indirectness[1];inaccuracy[2]
Tabalumab vs Blisibimod	Indirect	low	Indirectness[1];inaccuracy[2]
Tabalumab vs Placebo	Direct	Medium	inaccuracy[2]

Tabalumab vs E6742	Indirect	low	Indirectness[1];inaccuracy[2]
Tabalumab vs deucravacitinib	Indirect	low	Indirectness[1];inaccuracy[2]
Tabalumab vs Baricitinib	Indirect	low	Indirectness[1];inaccuracy[2]
Tabalumab vs Telitacicept	Indirect	low	Indirectness[1];inaccuracy[2]
Blisibimod vs Placebo	Direct	Medium	inaccuracy[2]
Blisibimod vs E6742	Indirect	low	Indirectness[1];inaccuracy[2]
Blisibimod vs deucravacitinib	Indirect	low	Indirectness[1];inaccuracy[2]
Blisibimod vs Baricitinib	Indirect	low	Indirectness[1];inaccuracy[2]
Blisibimod vs Telitacicept	Indirect	low	Indirectness[1];inaccuracy[2]
Placebo vs E6742	Direct	Medium	inaccuracy[2]
Placebo vs deucravacitinib	Direct	Medium	inaccuracy[2]
Placebo vs Baricitinib	Direct	Medium	inaccuracy[2]
Placebo vs Telitacicept	Direct	Medium	inaccuracy[2]
E6742 vs deucravacitinib	Indirect	low	Indirectness[1];inaccuracy[2]
E6742 vs Baricitinib	Indirect	low	Indirectness[1];inaccuracy[2]
E6742 vs Telitacicept	Indirect	low	Indirectness[1];inaccuracy[2]
deucravacitinib vs Baricitinib	Indirect	low	Indirectness[1];inaccuracy[2]
deucravacitinib vs Telitacicept	Indirect	low	Indirectness[1];inaccuracy[2]
Baricitinib vs Telitacicept	Indirect	low	Indirectness[1];inaccuracy[2]

notes:1: No head-to-head RCTs, evidence derived from indirect comparisons via placebo; 2: 95% CI crossing the null line.

Appendix 11 Sensitivity analysis of SRI-4 merger (SELENA-SLEDAI vs SLEDAI-2K)

Table S35 : SUCRA Rankings of sensitivity analysis for SRI-4 merger.

Treatment	SUCRA	PrBest	MeanRank
Telitacicept	97	59.8	1.6
Ustekinumab	86.4	15.5	3.6
IL-2	83.5	21.4	4.1
ABBV-599high-dose	73.3	1.9	6.1
Deucravacitinib	66.4	0.1	7.4
Anifrolumab	56.7	0	9.2
Upadacitinib	55.7	0.3	9.4
Sifalimumab	55.3	0	9.5
Iberdomide	53.9	0	9.8
Belimumab	52.3	0	10.1
Atacicept	51.4	0	10.2
ILT-101	45.7	0.4	11.3
F04236921	44.3	0.4	11.6
Dapirolizumabpegol	44.2	0.1	11.6
Tabalumab	35	0	13.3
Evobrutinib	30.2	0	14.3
Baricitinib	24.8	0	15.3
Blisibimod	24.3	0	15.4
Epratuzumab	13.4	0	17.5
Placebo	6.2	0	18.8

**Telitacicept**																			
1.53 (0.55,4.30)	**Ustekinumab**																		
1.50 (0.44,5.15)	0.98 (0.25,3.78)	**IL-2**																	
2.19 (0.88,5.47)	1.43 (0.49,4.16)	1.46 (0.41,5.16)	**ABBV-599 high dose**																
**2.54 (1.15,5.59)**	1.65 (0.63,4.33)	1.69 (0.52,5.47)	1.16 (0.50,2.69)	**Deucravacitinib**															
**2.94 (1.51,5.72)**	1.92 (0.81,4.55)	1.96 (0.65,5.85)	1.34 (0.65,2.77)	1.16 (0.67,2.02)	**Anifrolumab**														
**2.82 (1.11,7.14)**	1.84 (0.62,5.42)	1.88 (0.52,6.70)	1.29 (0.65,2.57)	1.11 (0.47,2.61)	0.96 (0.46,2.01)	**Upadacitinib**													
**2.94 (1.38,6.26)**	1.92 (0.75,4.89)	1.96 (0.62,6.20)	1.34 (0.60,3.02)	1.16 (0.60,2.25)	1.00 (0.60,1.66)	1.04 (0.46,2.38)	**Sifalimumab**												
**2.95 (1.31,6.64)**	1.92 (0.72,5.13)	1.96 (0.60,6.45)	1.35 (0.57,3.19)	1.16 (0.56,2.40)	1.00 (0.56,1.80)	1.05 (0.44,2.51)	1.00 (0.50,1.99)	**Iberdomide**											
**3.09 (1.64,5.82)**	2.02 (0.87,4.67)	2.06 (0.70,6.04)	1.41 (0.70,2.83)	1.22 (0.73,2.04)	1.05 (0.78,1.41)	1.10 (0.54,2.24)	1.05 (0.66,1.67)	1.05 (0.61,1.81)	**Belimumab**										
**3.07 (1.41,6.70)**	2.00 (0.77,5.20)	2.04 (0.63,6.58)	1.40 (0.61,3.22)	1.21 (0.61,2.41)	1.04 (0.61,1.80)	1.09 (0.47,2.54)	1.04 (0.54,2.00)	1.04 (0.51,2.12)	0.99 (0.60,1.64)	**Atacicept**									
**3.21 (1.15,8.92)**	2.09 (0.65,6.68)	2.13 (0.56,8.18)	1.46 (0.51,4.24)	1.26 (0.49,3.29)	1.09 (0.46,2.57)	1.14 (0.39,3.33)	1.09 (0.43,2.76)	1.09 (0.41,2.88)	1.04 (0.45,2.38)	1.05 (0.40,2.70)	**ILT-101**								
**3.31 (1.23,8.91)**	2.15 (0.69,6.70)	2.20 (0.59,8.23)	1.51 (0.54,4.24)	1.30 (0.52,3.28)	1.12 (0.50,2.54)	1.17 (0.41,3.33)	1.12 (0.46,2.74)	1.12 (0.44,2.87)	1.07 (0.48,2.36)	1.08 (0.43,2.69)	1.03 (0.33,3.18)	**F04236921**							
**3.32 (1.32,8.38)**	2.16 (0.74,6.36)	2.21 (0.62,7.87)	1.52 (0.57,4.00)	1.31 (0.56,3.06)	1.13 (0.54,2.36)	1.18 (0.44,3.14)	1.13 (0.50,2.56)	1.12 (0.47,2.69)	1.07 (0.53,2.18)	1.08 (0.47,2.51)	1.03 (0.35,3.02)	1.00 (0.35,2.84)	**Dapirolizum abpegol**						
**3.64 (1.88,7.07)**	2.37 (1.00,5.63)	2.42 (0.81,7.24)	1.66 (0.81,3.43)	1.44 (0.82,2.50)	1.24 (0.87,1.77)	1.29 (0.62,2.71)	1.24 (0.75,2.05)	1.23 (0.69,2.21)	1.18 (0.88,1.58)	1.19 (0.69,2.04)	1.14 (0.48,2.67)	1.10 (0.49,2.49)	1.10 (0.53,2.29)	**Tabalumab**					
**3.84 (1.82,8.10)**	2.50 (0.99,6.34)	2.55 (0.81,8.05)	1.75 (0.79,3.91)	1.51 (0.79,2.91)	1.31 (0.80,2.14)	1.36 (0.60,3.08)	1.30 (0.71,2.40)	1.30 (0.66,2.56)	1.24 (0.79,1.95)	1.25 (0.66,2.38)	1.20 (0.48,3.01)	1.16 (0.48,2.82)	1.16 (0.51,2.60)	1.05 (0.64,1.73)	**Evobrutinib**				
**4.02 (2.11,7.66)**	**2.62 (1.12,6.12)**	2.67 (0.90,7.91)	1.84 (0.91,3.72)	1.58 (0.93,2.70)	1.37 (0.99,1.88)	1.43 (0.69,2.94)	1.37 (0.85,2.21)	1.36 (0.78,2.39)	**1.30 (1.02,1.66)**	1.31 (0.78,2.20)	1.25 (0.54,2.90)	1.22 (0.55,2.71)	1.21 (0.59,2.49)	1.10 (0.80,1.52)	1.05 (0.66,1.67)	**Baricitinib**			
**4.08 (1.99,8.40)**	**2.66 (1.07,6.60)**	2.72 (0.88,8.42)	1.86 (0.86,4.06)	1.61 (0.86,3.00)	1.39 (0.88,2.19)	1.45 (0.66,3.20)	1.39 (0.78,2.47)	1.38 (0.72,2.65)	1.32 (0.88,1.98)	1.33 (0.72,2.45)	1.27 (0.52,3.13)	1.24 (0.52,2.93)	1.23 (0.56,2.71)	1.12 (0.71,1.76)	1.06 (0.60,1.88)	1.02 (0.66,1.55)	**Blisibimod**		
**4.58 (2.38,8.81)**	**2.98 (1.27,7.02)**	**3.04 (1.02,9.05)**	**2.09 (1.02,4.27)**	**1.80 (1.05,3.11)**	**1.56 (1.11,2.18)**	1.62 (0.78,3.37)	1.56 (0.95,2.54)	1.55 (0.87,2.75)	**1.48 (1.13,1.94)**	1.49 (0.88,2.54)	1.43 (0.61,3.33)	1.39 (0.62,3.11)	1.38 (0.67,2.85)	1.26 (0.90,1.76)	1.19 (0.74,1.93)	1.14 (0.85,1.53)	1.12 (0.72,1.74)	**Epratuzumab**	
**4.94 (2.67,9.13)**	**3.22 (1.41,7.35)**	**3.28 (1.13,9.54)**	**2.25 (1.14,4.45)**	**1.95 (1.19,3.19)**	**1.68 (1.30,2.16)**	1.75 (0.87,3.51)	**1.68 (1.08,2.60)**	1.67 (0.99,2.84)	**1.60 (1.37,1.86)**	1.61 (0.99,2.60)	1.54 (0.68,3.49)	1.49 (0.69,3.25)	1.49 (0.74,2.97)	**1.36 (1.06,1.74)**	1.29 (0.84,1.97)	**1.23 (1.01,1.49)**	1.21 (0.83,1.76)	1.08 (0.86,1.35)	**Placebo**

Figure S25 Network meta-analysis results of sensitivity analysis for SRI-4 merger.
